# Ki-67 regulates heterochromatin organization in neurons and forms condensates with heterochromatic components

**DOI:** 10.1038/s41467-026-77430-2

**Published:** 2026-09-08

**Authors:** Lannah S. Abasi, Challana Tea, Rushabh Bhakta, Donald Le, Tien M. Phan, Jeetain Mittal, Cole J. Ferguson, Galia T. Debelouchina

**Affiliations:** 1 Department of Chemistry and Biochemistry, University of California San Diego, La Jolla, CA USA; 2 Department of Pathology, University of California San Diego, La Jolla, CA USA; 3 Artie McFerrin Department of Chemical Engineering, Texas A&M University, College Station, TX, USA; 4 Department of Chemistry, Texas A&M University, College Station, TX, USA; 5 Interdisciplinary Graduate Program in Genetics and Genomics, Texas A&M University, College Station, TX, USA

**Keywords:** Nuclear organization, Biopolymers in vivo, Chromatin, Developmental disorders, Gene regulation

## Abstract

The dysregulation of chromatin organization has been associated with numerous forms of neurological disease. In the *ANAPC7* neurodevelopmental syndrome, a disease mechanistically linked to defective proteasomal degradation, the mitotic protein Ki-67 accumulates abnormally in heterochromatin compartments. Here, we investigate Ki-67’s role in heterochromatin through in vivo examination of neurons from two relevant genetic mouse models, in vitro assays and computational studies. We find that Ki-67 modulates the size and number of heterochromatin compartments in neurons. Furthermore, we demonstrate that an engineered Ki-67 construct enhances the propensity of HP1α and nucleosome arrays to form condensates in vitro, a process that is mediated through a complex network of electrostatic interactions and is robust to relevant histone post-translational modifications. These observations suggest that Ki-67 may regulate heterochromatin through a phase separation mechanism involving additional heterochromatin components, thus providing insights into the roles of Ki-67 during both typical neurodevelopment and in disease.

## Abstract (web summary)

Chromatin organisation is critical for neuronal function, and its disruption is linked to neurodevelopmental disease. Here, the authors show that Ki‑67 regulates heterochromatin compartment organisation and promotes HP1α-chromatin condensation, implicating phase separation in both normal neurodevelopment and ANAPC7 syndrome.

## Introduction

Chromatin, the complex of DNA and associated proteins, forms the essential structure of chromosomes in all eukaryotic cells. Cells must establish and maintain exquisite chromatin organization across several orders of magnitude to accomplish the remarkable feat of compacting two meters of linear genome into a nucleus whose volume measures just a few hundred cubic microns. The maintenance of a functional genome requires a complex array of molecular interactions and compartment-defining biophysical properties, which emerge from the biochemical composition at smaller length scales. Unsurprisingly, our mechanistic understanding of how these astonishingly complex yet fundamental processes underlie health and contribute to disease remains incomplete.

In the brain, dysregulation of chromatin state is a pathogenic mechanism that contributes to multiple forms of neurologic disease^1–3^. The developing brain is especially susceptible to perturbations in chromatin regulation, and mutations in chromatin regulatory pathways are a major contributor to inherited disorders of neurodevelopment^4–6^. For instance, the *ANAPC7* neurodevelopmental syndrome (Ferguson-Bonni syndrome, OMIM 619699) is an inherited recessive disorder of cognition, mechanistically linked to dysfunction of the heterochromatin compartment^7^. In this syndrome, homozygous loss of the APC7 subunit of the ubiquitin ligase Anaphase-Promoting Complex impairs proteasomal degradation of specific substrates. Mice homozygous for null mutations in *Anapc7* exhibit developmental delay with persistent behavioral deficits. Proteomic evaluation of the developing brain of mutant mice showed Ki-67 to be the most overabundant protein, with post-mitotic neurons exhibiting dramatic accumulation of Ki-67. Ki-67 is a nuclear protein with multifaceted roles in chromosome organization adapted to different stages of the cell cycle^8–10^. For instance, during mitosis, Ki-67 organizes the surfactant-like chromatin periphery compartment, preventing chromosome entanglement^11^. During mitotic exit, Ki-67 promotes chromosome clustering in daughter cells as the nuclear envelope reforms^11–14^. In interphase, Ki-67 associates with the nucleolus and constitutive heterochromatin, a gene-poor compartment that harbors mainly pericentromeric satellite sequences, transposable elements and some protein-coding genes^15^. In mice with loss of APC7, inadequately degraded Ki-67 accumulates within constitutive heterochromatin of neurons that have recently exited the cell cycle^7^. Compared to its role in mitosis, which is well documented, how Ki-67 contributes to the formation and function of heterochromatin, especially in the disease-relevant context of differentiating neurons, remains poorly understood.

Ki-67 has the capability to influence chromatin via multiple mechanisms. Ki-67 is thought to directly associate with DNA and chromatin through its forkhead-associated (FHA) domain and leucine-arginine rich (LR) domain^16–18^. Ki-67 also binds heterochromatin protein 1 alpha (HP1α)^19,20^, a component of constitutive heterochromatin that recruits repressive factors and plays a critical role in nucleosome compaction^21–24^. HP1α is believed to function through the process of liquid-liquid phase separation (LLPS), a physicochemical mechanism which results in the formation of biomolecular condensates, and which is thought to play a role in chromatin organization across scales^23,25–28^. Through this process, constitutive heterochromatin can form spatially bounded structures with distinct material properties and composition^25–29^.

Intrigued by the potential interplay between Ki-67 and HP1α, we sought to test the hypothesis that Ki-67 influences the ability of HP1α to drive phase separation of constitutive heterochromatin environments. We investigate the influence of Ki-67 on heterochromatin through an in vivo examination of mouse neurons, discovering that Ki-67 modulates the size and number of heterochromatin compartments without substantial changes in epigenetic state or transcriptome. Using in vitro reconstituted assays, we show that an engineered minimal construct of Ki-67 modulates the propensity of HP1α to form condensates with nucleosome arrays in vitro, a process that is robust to the presence of relevant histone post-translational modifications but dependent on the phosphorylation state of Ki-67 and HP1α. Finally, we use nuclear magnetic resonance (NMR) spectroscopy and molecular dynamics simulations to characterize the interactions between Ki-67 and HP1α and uncover extensive electrostatic networks that drive the co-partitioning of the two proteins. These experiments provide fundamental insight into how the interplay between Ki-67 and HP1α can contribute to the regulation of constitutive heterochromatin domains, with relevance to both typical neurodevelopment and neurodevelopmental disorders.

## Results

### Perturbing Ki-67 in differentiating neurons disrupts heterochromatin organization without affecting epigenetic state or the transcriptome

To investigate how Ki-67 influences heterochromatin organization in neurons, we turned to genetic models with either Ki-67 overabundance or depletion. As we previously reported^7,30^, loss of APC7 in mice homozygous for the mutant *Anapc7* allele that recapitulates the patient mutation, causes accumulation of Ki-67 in constitutive heterochromatin of post-mitotic neurons. We generated a complementary Ki-67 mutant by crossing mice harboring floxed exon 4 of *Mki67* (the gene encoding Ki-67) with mice expressing cre recombinase under the influence of the Sox2 promoter, which is expressed in oocytes and fertilized eggs. Whereas previously published mutants harbored hypomorphic *Mki67* alleles that depleted Ki-67 via mutation of exon 1^31^, deletion of exon 4 should completely eliminate the production of all Ki-67 isoforms.

When assessing the general health of *Mki67*^-/-^ mice, we noticed that both sexes were born at lower than Mendelian ratios, suggesting that Ki-67 contributes to embryonic viability (Supplementary Fig. 1a, b). Their body weight was persistently reduced from a young age into adulthood, indicating developmental delay, a phenotype that is often associated with abnormal neurodevelopment (Supplementary Fig. 1c, d). The cerebellum, a brain region whose cellularity and volume rely upon massive expansion of granule neurons during the postnatal period from P4-P16, exhibited hypoplasia (Supplementary Fig. 1e). Microscopic evaluation of the mutant cerebellum by Calbindin immunofluorescence demonstrated heterotopias of Purkinje neurons, the main synaptic target of granule neuron innervation (Supplementary Fig. 1f). This dysplastic phenotype commonly results from inadequate production of granule neurons. These observations motivated our examination of cellular deficits in cerebellar granule neurons.

**Fig. 1:**
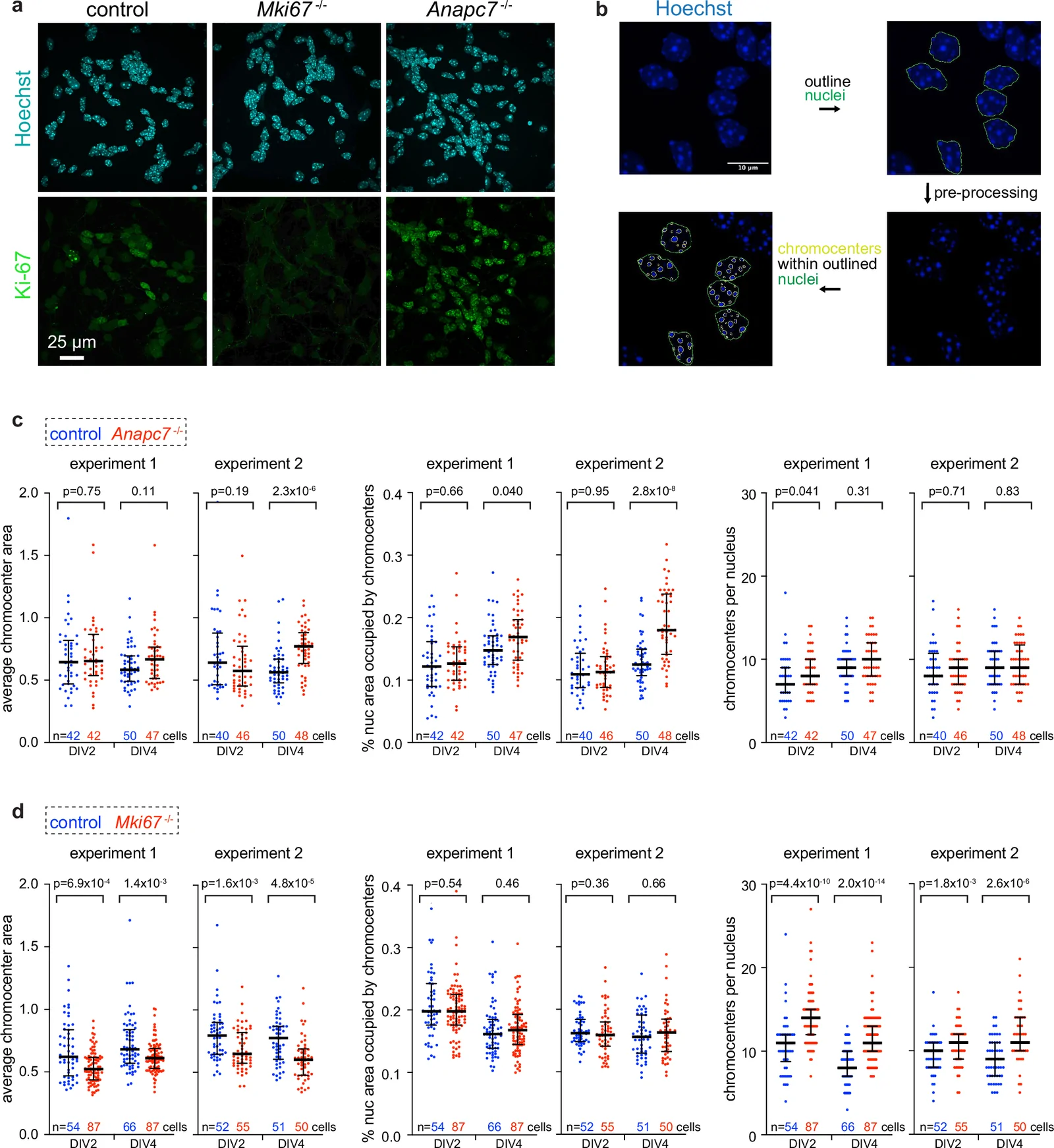
Dysregulation of Ki-67 affects the size and distribution of chromocenters in neurons. **a** Immunofluorescence (IF) detection of Ki-67 by confocal microscopy of cultured primary cerebellar granule neurons (CGN) at day 2 in vitro (DIV2). **b** Procedure for isolation of Hoechst-labeled chromocenters (constitutive heterochromatin) in confocal micrographs. **c** Quantitation of chromocenter parameters in *Anapc7* ^-/-^ CGNs on DIV2 and DIV4. Each data point represents a nucleus. Error bars show median with interquartile range. *P*-values by Mann-Whitney U test. Independent experiments are shown from two biological replicates from separate litters. Source data are provided as a source data file. **d** Quantitation of chromocenter parameters in *Mki67*^*-/-*^ CGNs on DIV2 and DIV4. Each data point represents a nucleus from two biological replicates. Error bars show median with interquartile range. *P*-values by Mann-Whitney U test. Independent experiments are shown from two biological replicates from separate litters. Source data are provided as a source data file.

To examine the effects of dysregulating Ki-67 on heterochromatin, we cultured neurons from the cerebellum of control, *Anapc7* ^-/-^ and *Mki67* ^-/-^ mice. When isolated from P7 mice and subjected to primary culture ex vivo, cerebellar granule neurons (CGN) semi-synchronously undergo similar developmental and morphogenic processes as those that occur in vivo, permitting the examination of the immature neurons at day 2 in vitro (DIV2) and a more mature stage (DIV4-5)^32^. Immunofluorescence detection of Ki-67 at DIV2 demonstrated the predicted patterns: increased abundance in *Anapc7*^-/-^ neurons and absence in *Mki67* ^-/-^ neurons (Fig. 1a). We visualized constitutive heterochromatin by detecting chromocenters—brightly stained nuclear Ki-67–positive structures containing highly condensed chromatin, which are labeled by the DNA dye Hoechst (bisbenzimide). Quantitating the number and size of chromocenters at DIV4 demonstrated that loss of APC7 led to an increase in chromocenter area and the portion of the nucleus occupied by chromocenters, without affecting chromocenter number (Fig. 1b, c). In contrast, loss of Ki-67 resulted in a decrease in chromocenter size that was accompanied by an increase in the number of chromocenters within each nucleus (Fig. 1d). These results suggest that Ki-67 impacts the organization of heterochromatic foci in neuronal nuclei.

After observing the effects of Ki-67 loss on constitutive heterochromatin, we wondered whether mutant neurons would demonstrate alterations in the abundance and/or distribution of heterochromatin-associated histone modifications. Therefore, we mapped these modifications in the immature cerebellum of control and *Mki67* ^-/-^ mice at age P13 using CUT&RUN (Cleavage Under Targets and Release Using Nuclease). This timepoint represents a tradeoff between including neurons undergoing post-mitotic differentiation, a period when Ki-67 participates in the formation of constitutive heterochromatin, while minimizing the number of mitotic progenitors^33^. Constitutive heterochromatin contains the modification H3K9me3 (histone 3 trimethylated at Lys9) that, in general, has a similar distribution across different tissues and developmental timepoints^34,35^. In contrast, H3K27me3 marks facultative heterochromatin, which is associated with regions that are silenced with tissue or developmental stage specificity^36^. We also examined the euchromatin-associated modifications H3K4me3 (active promoters) and H3K27ac (active enhancers). These modifications exhibited the predicted patterns: overlapping H3K4me3 and H3K27ac peaks and H3K9me3 occupying its own compartment-specific distribution (Supplementary Fig. 2a). Surprisingly, CUT&RUN analysis of the cerebellum of Mki67 ^-/-^ mice failed to demonstrate significant differences in these histone modifications, either at the level of individual loci, or in genome-wide aggregate analyses in which each modification was plotted across all protein-coding genes (Supplementary Fig. 2b). These results indicate that Ki-67 expression in neural progenitors is unnecessary for epigenetic regulation of heterochromatin domains.

Previous reports indicate that Ki-67 could regulate transcription^37^; therefore, we examined transcript abundance in the developing cerebellum of *Mki67* ^-/-^ mice using RNAseq (Supplementary Fig. 3). To more directly assess transcription and diminish the influence of post-transcriptional regulation, we input RNA derived from nuclei isolated from the P10-11 cerebellum after depleting ribosomal RNAs. We failed to observe large differences in the protein-coding transcriptome when genome-wide data was visualized in aggregate heatmaps (Supplementary Fig. 3a), and differential analysis of transcript abundance in biological replicates identified only a handful of significantly up or downregulated loci (Supplementary Fig. 3b). Importantly, *Mki67* was among the significantly downregulated loci (Supplementary Fig. 3c, d). Interestingly, the abundance of non-coding RNAs derived from the pericentromeric major satellite repeats, which were reduced in the cerebellum of *Anapc7* ^-/-^ mutant mice in a Ki-67–dependent manner^7^, was unaffected in the cerebellum of *Mki67* ^-/-^ mice (Supplementary Fig. 3e). Therefore, Ki-67 is unnecessary for the generation of the developing cerebellum’s transcriptome.

In summary, our analysis of the *Anapc7*^-/-^ and *Mki67*^-/-^ genetic mouse models suggests that the overabundance or depletion of Ki-67 primarily impacts the spatial organization of neuronal heterochromatin. While the depletion of Ki-67 leads to neurodevelopmental phenotypes in *Mki67* ^-/-^, we do not detect substantial changes in the transcriptome or epigenome of cerebellar granule mouse neurons. This prompted us to investigate other possible mechanisms that may regulate chromocenter formation, including the potential ability of Ki-67 to influence the phase separation of components of heterochromatin.

### Ki-67–positive nuclear bodies exhibit condensate-like properties

A growing body of literature has suggested that constitutive heterochromatin may be a distinct nuclear compartment that forms through the process of phase separation^23,25–28^. After observing the effects of perturbing Ki-67 on the number and size of chromocenters, we considered the possibility that the association of Ki-67 with constitutive heterochromatin and its activity within this compartment could depend upon the process of phase separation. Ki-67 is a massive ~ 360 kDa protein with sequence features that are common in phase separating proteins, including charge patterning and lengthy disordered repeat regions^38^. Furthermore, previous studies have suggested that constructs containing portions of the N-terminal region of the protein or the R12 repeat and the C-terminal LR domain can form condensates in cells and in vitro, suggesting that Ki-67 phase separation is a plausible mechanism in the heterochromatin context^39,40^.

**Fig. 2:**
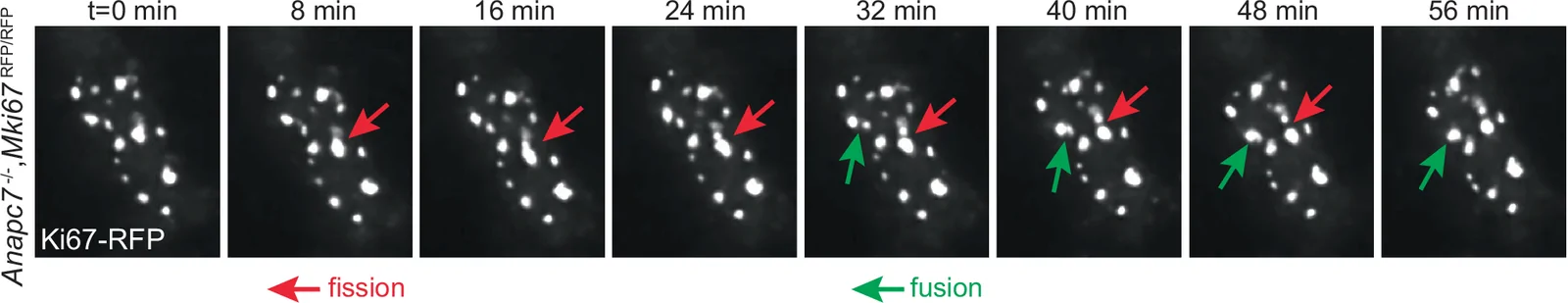
Liquid-like behavior of Ki-67 in neurons. Live-cell imaging of endogenous Ki-67 in neurons demonstrates fission and fusion of constitutive heterochromatin. Spinning disc and confocal microscopy detection of Ki-67 with TagRFP knocked-in-frame into the C-terminus of the *Mki67* locus (*Mki67* ^RFP^). Live cerebellar neurons in primary culture from *Anapc7* ^-/-^,*Mki67* ^RFP/RFP^ were imaged using spinning disc and confocal microscopy, followed by artificial intelligence reconstruction, an approach that permitted imaging of the nuclear bodies with high spatial and temporal resolution.

To determine if Ki-67-positive particles exhibited condensate-like behavior in neurons, we visualized Ki-67 using an RFP reporter knocked-in at the C-terminus of the endogenous *Mki67* locus (*Mki67* ^RFP^)^41^. To enhance fluorescence during live-cell imaging, we bred both this allele and the *Anapc7* mutant allele to homozygosity before examining cultured cerebellar neurons from these *Anapc7* ^-/-^,*Mki67* ^RFP/RFP^ mice. We observed Ki-67-positive particles undergo homotypic fusion indicative of liquid-like condensate behavior (Fig. 2). As previous experiments established that the large majority of RFP-tagged Ki-67 overlaps with chromocenters^7^, our observations indicate that the behavior of Ki-67–positive structures likely reflects the behavior of constitutive heterochromatin compartments.

Proteins that undergo condensate formation are often redistributed in cells when exposed to 1,6-hexanediol^42^. Treating cultured neurons at DIV2 with 1,6-hexanediol at a concentration of 7% for 10 min led to rapid relocalization of Ki-67 away from constitutive heterochromatin and into nucleoli (Supplementary Fig. 4a–c). Quantitating the intensity of the Ki-67 immunofluorescence signal within nucleoli (as detected by anti-nucleolin immunofluorescence) demonstrated an increase in Ki-67 upon treatment with 1,6-hexanediol (Supplementary Fig. 4d). Subsequent analysis of the degree to which the Ki-67 and nucleolin signals were correlated within individual nuclei revealed increases in the Pearson R values following treatment (Supplementary Fig. 4e). Correspondingly, hexanediol treatment reduced the correlation between Ki-67 and Hoechst-labeled chromocenters (Supplementary Fig. 4f). We also observed Ki-67–RFP redistribution upon hexanediol treatment in cultured neurons from both *Anapc7* ^+/+^,*Mki67* ^RFP/RFP^ and *Anapc7* ^-/-^,*Mki67* ^RFP/RFP^ mice (Supplementary Fig. 5a, b). While we acknowledge the well-documented limitations of 1,6-hexanediol, including its toxicity and effects on nuclear pores, kinases and phosphatases^43,44^, taken together, our findings in neurons are compatible with a model in which Ki-67 is involved in a process consistent with phase separation. These observations prompted us to investigate this process further by employing reconstituted in vitro systems to determine how Ki-67 influences the properties of heterochromatin condensates, as described below.

### A minimal Ki-67 construct co-localizes with heterochromatin environments in cells

**Fig. 3:**
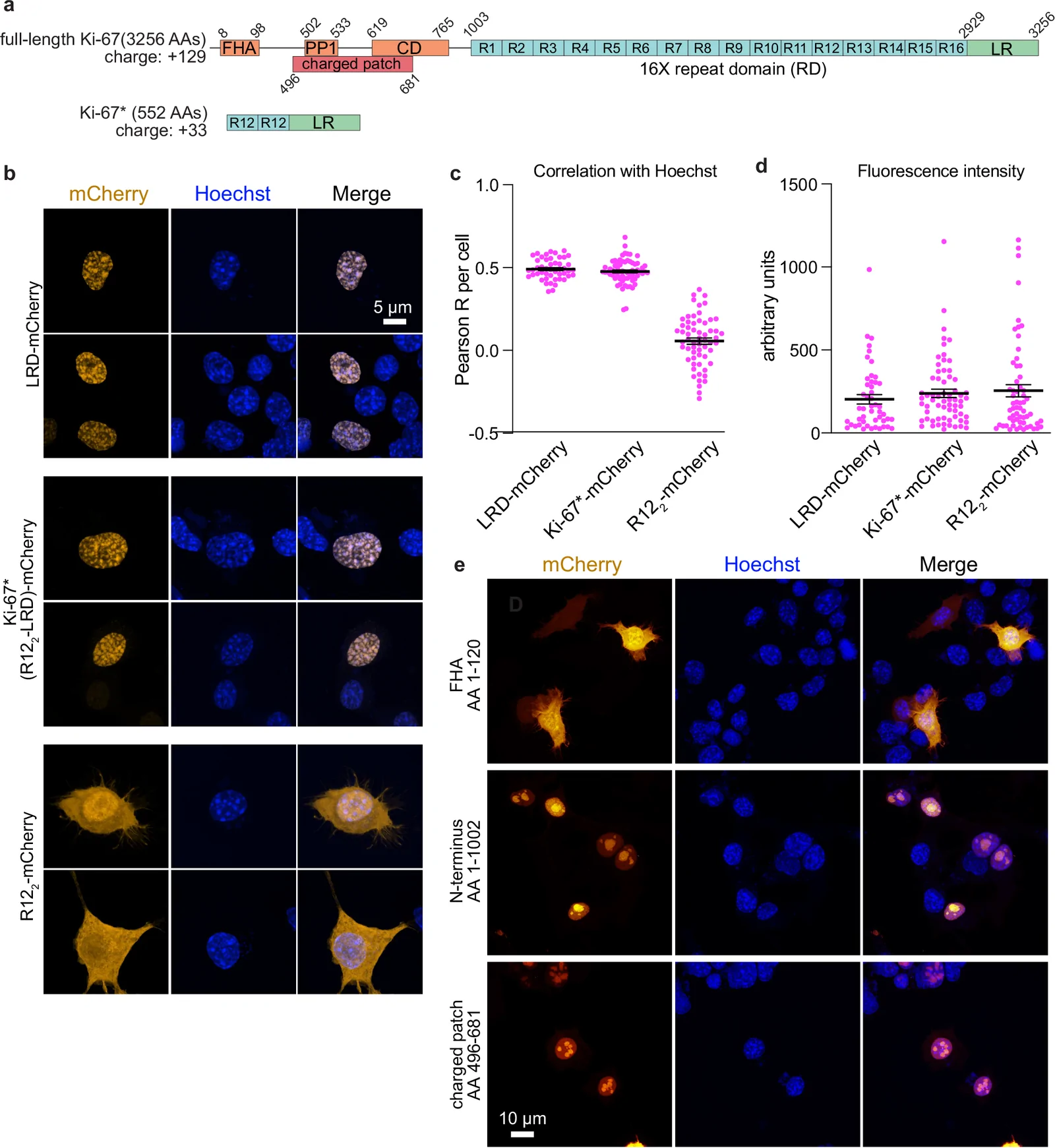
Subcellular localization of Ki-67* and related constructs in transiently transfected N2a cells. **a** Domain architecture of full-length Ki-67 and Ki-67*. Amino acid numbers shown above Ki-67. Domains are not drawn to scale. FHA – Forkhead-associated domain; CP – charged patch; PP1 – protein phosphatase 1 binding domain; CD – conserved domain. **b** Representative images of N2a neuroblastoma cell line transfected with plasmids expressing mCherry-tagged LRD, R12_2_-LRD (i.e., Ki-67*) or R12_2_ and stained with Hoechst. **c** Subcellular correlation (Pearson R) of each of these first three constructs with Hoechst. Each dot represents the nucleus of a single transfected cell. Quantitation collected from two independent transfection experiments. Error bars represent the standard error of the mean.* p*-values by Kruskal-Wallis test followed by Dunn’s test. Source data are provided as a source data file. **d** Quantitation of fluorescence intensities of cells included in localization measurements. Quantitation collected from two independent transfection experiments. Error bars represent the standard error of the mean. Source data are provided as a source data file.** e** Localization of additional domains of Ki-67 in transfected N2a cells.

Given our evidence that the presence or absence of Ki-67 could reorganize chromocenters in neurons, we shifted our focus to biophysical and biochemical studies aimed at elucidating how Ki-67 could influence the properties of heterochromatin components. Since the full-length Ki-67 construct is too large to be expressed and purified by standard methods, we sought to generate a truncated version that embodies its essential molecular attributes. Ki-67 consists of an N-terminal forkhead-associated (FHA) domain, a protein phosphatase 1 (PP1) binding domain, a conserved domain (CD) of uncertain function, a repeat domain consisting of 16 disordered Ki-67 repeats arranged in tandem, and a C-terminal LR (leucine/arginine-rich) chromatin-binding domain (Fig. 3a)^10^. The LR domain (hereafter LRD) drives the association of Ki-67 with chromatin through its ability to bind DNA^18^, chromatin^16,17^, and HP1 proteins^19,20^, while the disordered Ki-67 repeat domains are thought to mediate phase separation^40^. To investigate Ki-67’s phase separation-promoting activity within chromatin, we settled on a construct engineered by Yamazaki et al. for their Ki-67 phase separation studies in the presence of crowding agents^40^. This 552 amino acid construct, which we termed Ki-67*, consists of two copies of the 12th repeat (R12) coupled to the LRD (Fig. 3a) and offers several important advantages. First, it includes an amino acid content that is comparable to full-length Ki-67, including a high degree of disorder (99% disordered as predicted by IUPred^45^) and a predicted charge of +33 at pH 7.2, resulting from the abundance of basic amino acids (Supplementary Fig. 6). Second, this construct can be prepared recombinantly in *E. coli* in high yields and purity as required for biophysical and biochemical studies (Supplementary Fig. 7). Finally, the R12 domain harbors many phosphorylation sites, permitting an assessment of the effects of Ki-67 phosphorylation on its behavior^40^.

To determine whether this minimal construct recapitulates the observed association of Ki-67 with constitutive heterochromatin environments, we expressed mCherry-tagged Ki-67* in N2a cells, a mouse neuroblastoma cell line. Ki-67* localized to the nucleus in strong correlation with Hoechst-labeled chromocenters, as did mCherry-tagged LRD (Fig. 3b). In contrast, the R12_2_ fragment lacking the LRD was dispersed throughout the cytoplasm and nucleus. Accordingly, the correlation between mCherry and Hoechst-labeled chromocenters was lower for R12_2_-mCherry than the LRD-containing constructs (Fig. 3c). Fluorescence intensity was comparable for all three constructs (Fig. 3d). As controls, we also expressed constructs representing the other segments of the Ki-67 sequence. Notably, mCherry-tagged FHA exhibited nuclear and cytoplasmic localization without enrichment within heterochromatin, whereas the Ki-67 N-terminus and charged patch did not overlap with constitutive heterochromatin but instead localized to nucleoli (Fig. 3e). Hence, heterochromatin localization appears to be mainly dictated by the LRD, which also has the ability to interact with HP1 proteins^19,20^. The association between Ki-67 and HP1 proteins was further suggested by analysis of our cultured wild-type *Anapc7* ^-/-^ cerebellar granule neurons, which showed the colocalization of endogenous Ki-67 with HP1α in constitutive heterochromatin (Supplementary Fig. 8a, b). Taken together, these observations indicated that Ki-67* is suitable and tractable for in vitro investigation of interactions with heterochromatin components such as nucleic acids, chromatin and HP1α in the context of phase separation.

### Ki-67* forms condensates with heterochromatin components in vitro

**Fig. 4:**
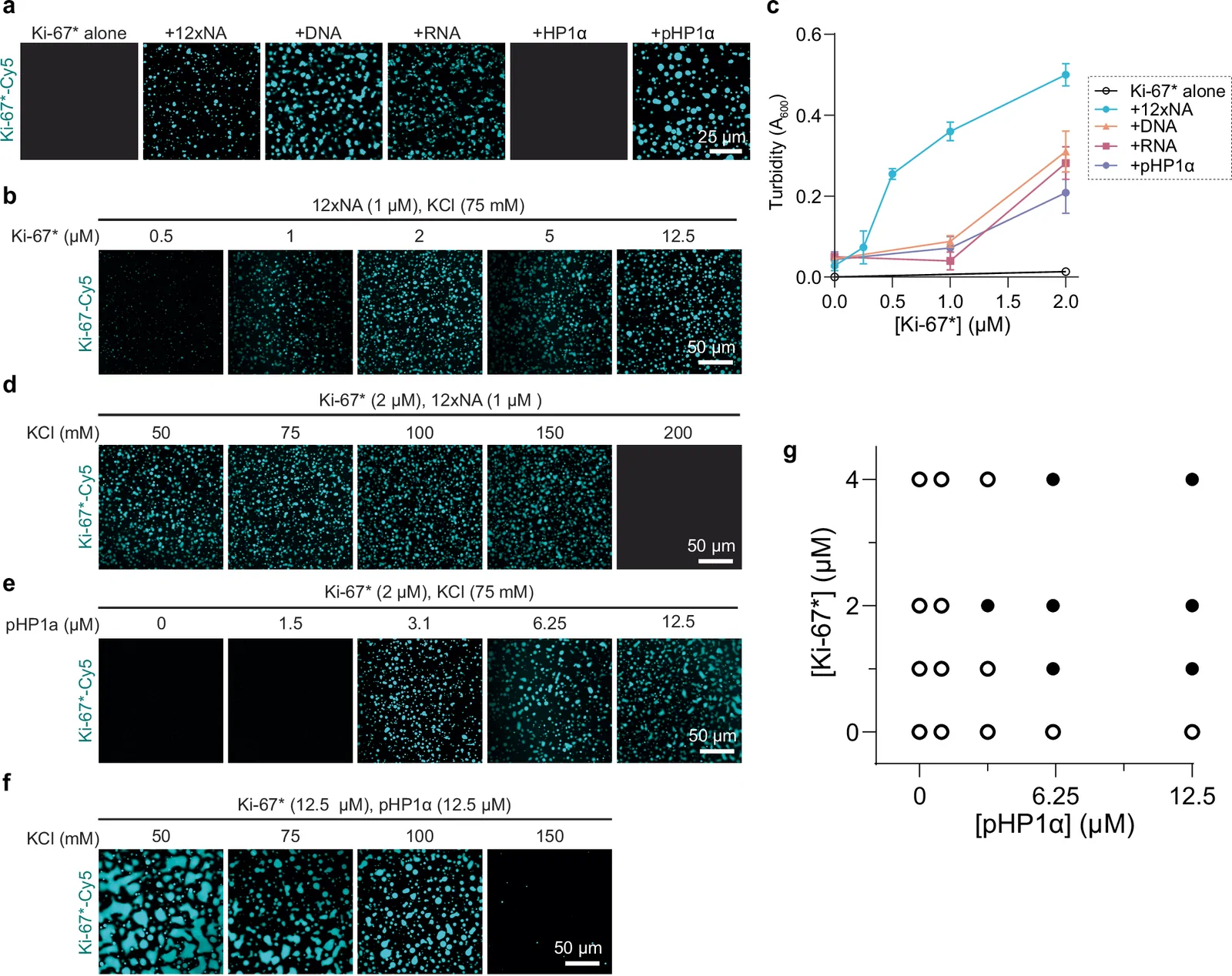
Ki-67* forms condensates with its interactors under physiological conditions. **a** Confocal micrographs of droplets of 2 μM Ki-67* alone or with 40 nM 12mer nucleosome arrays (12xNA; 0.5 μM equivalent of mononucleosomes), 75 ng/μL 2.1 kbp DNA (equivalent DNA concentration to 12xNA), 75 ng/μL RNA (extracted from nuclei isolated from P11 mouse cerebellum), 3 μM HP1α, and 3 μM pHP1α, visualized with 5% Ki-67*-Cy5 (scale bar = 25 μm). Studies were conducted in 20 mM Tris buffer, 75 mM KCl, 0.5 mM TCEP, pH 7.2 at 25 °C. Colocalization images are found in Supplementary Fig. 9. **b** Confocal microscopy images of droplets of 80 nM 12xNA (1 μM equivalent of mononucleosomes) with variable Ki-67* concentrations, visualized with 5% Ki-67*-Cy5. **c** Turbidity at A600 of Ki-67* with different nuclear components with variable Ki-67* concentrations. The average of three biological replicates is shown (*n* = 3). Error bars represent standard deviation. Source data are provided as a source data file. **d** Confocal microscopy images of droplets of 80 nM 12xNA and 2 μM Ki-67* with variable salt concentrations, visualized with 5% Ki-67*-Cy5. **e** Confocal microscopy images of 2 μM Ki-67* with varying concentrations of pHP1α. Images are visualized with 5% Ki-67*-Cy5.** f** Confocal microscopy images of 12.5 μM Ki-67* and 12.5 μM pHP1α, at varying salt concentrations. Images are visualized with 5% Ki-67*-Cy5. **g** Phase diagram of Ki-67* liquid droplet formation at varying concentrations of pHP1α. Open dots indicate no phase separation, filled dots indicate phase separation. Each condition was assessed at least twice by microscopy.

While Ki-67* has previously been shown to undergo phase separation on its own in the presence of crowding agents^40^, here we focused on its ability to form condensates with heterochromatin components without the addition of molecular crowders. To this end, we combined Ki-67* with either chromatin polymers containing 12 nucleosomes^46^ (12mer nucleosome arrays, hereafter 12xNA), DNA, RNA, HP1α or phosphorylated HP1α (pHP1α). 12xNAs contain 12 histone octamers positioned along 12 repeats of the nucleosome positioning Widom 601 DNA sequence, altogether reaching a length of 2.1 kb^46^. We studied both N-terminally phosphorylated and non-phosphorylated forms of HP1α to evaluate the effect of the post-translational modification, which is known to promote phase separation^27,47–51^ and is constitutively present in cells^49^. Similar to previous observations^40^, in the absence of other components or crowding agents, Ki-67* did not phase separate on its own even at concentrations as high as 400 μM and low to moderate ionic strength. However, physiological concentrations of Cy5-labeled Ki-67* (2 µM) and KCl (75 mM) in combination with 12xNA (40 nM, equivalent to 75 ng/µL DNA), naked DNA (75 ng/μL), RNA (75 ng/uL), or pHP1α (3 μM) readily formed condensates (Fig. 4a). Monitoring the colocalization of Ki-67*-Cy5 with either Cy3-labeled pHP1α or 12xNA visualized via the DNA-intercalating fluorophore YOYO-1 showed that these components colocalized within condensates (Supplementary Fig. 9a–d). Meanwhile, Ki-67 did not phase separate with non-phosphorylated HP1α (Fig. 4a), even at high concentrations of HP1α and Ki-67 (Supplementary Fig. 9e, f).

To compare phase separation of Ki-67* between conditions and determine the concentration dependence of this phenomenon, we turned to turbidity assays, which quantitate condensate formation by measuring the degree to which suspensions scatter 600 nm light. Remarkably, phase separation of 12xNAs initiated with as little Ki-67* as 0.5 μM (Fig. 4b), increased with escalating concentrations of Ki-67* (Fig. 4c), and persisted in the presence of high salt (150 mM KCl) (Fig. 4d). Meanwhile, condensation of 2 μM Ki-67* occurred in the presence of DNA or RNA, resulting in similar turbidity profiles between both nucleic acids (Fig. 4a, c). Phase separation of pHP1α and Ki-67* required as little as 3 µM and 2 μM of each protein, respectively (Fig. 4e–g and Supplementary Fig. 9d), and persisted up to 100 mM KCl (Fig. 4f). This is noteworthy as pHP1α on its own only phase separates at concentrations between 50 – 100 μM^51,52^. Dependence upon ionic strength suggests that electrostatic interactions drive condensate formation, which is expected given the high charge density of Ki-67*. Despite the capacity for condensate formation to occur at higher ionic strength, we performed the remainder of our in vitro assays in the presence of 75 mM KCl for comparability with the prior HP1α phase separation literature, which shows weakened phase separation of pHP1α at KCl concentrations exceeding 100 mM KCl^27^. Taken together, our data demonstrate that Ki-67* readily forms condensates with heterochromatin components and significantly enhances the ability of pHP1α but not HP1α to undergo phase separation in vitro.

### Phosphorylation modestly decreases Ki-67* condensation with heterochromatin components

**Fig. 5:**
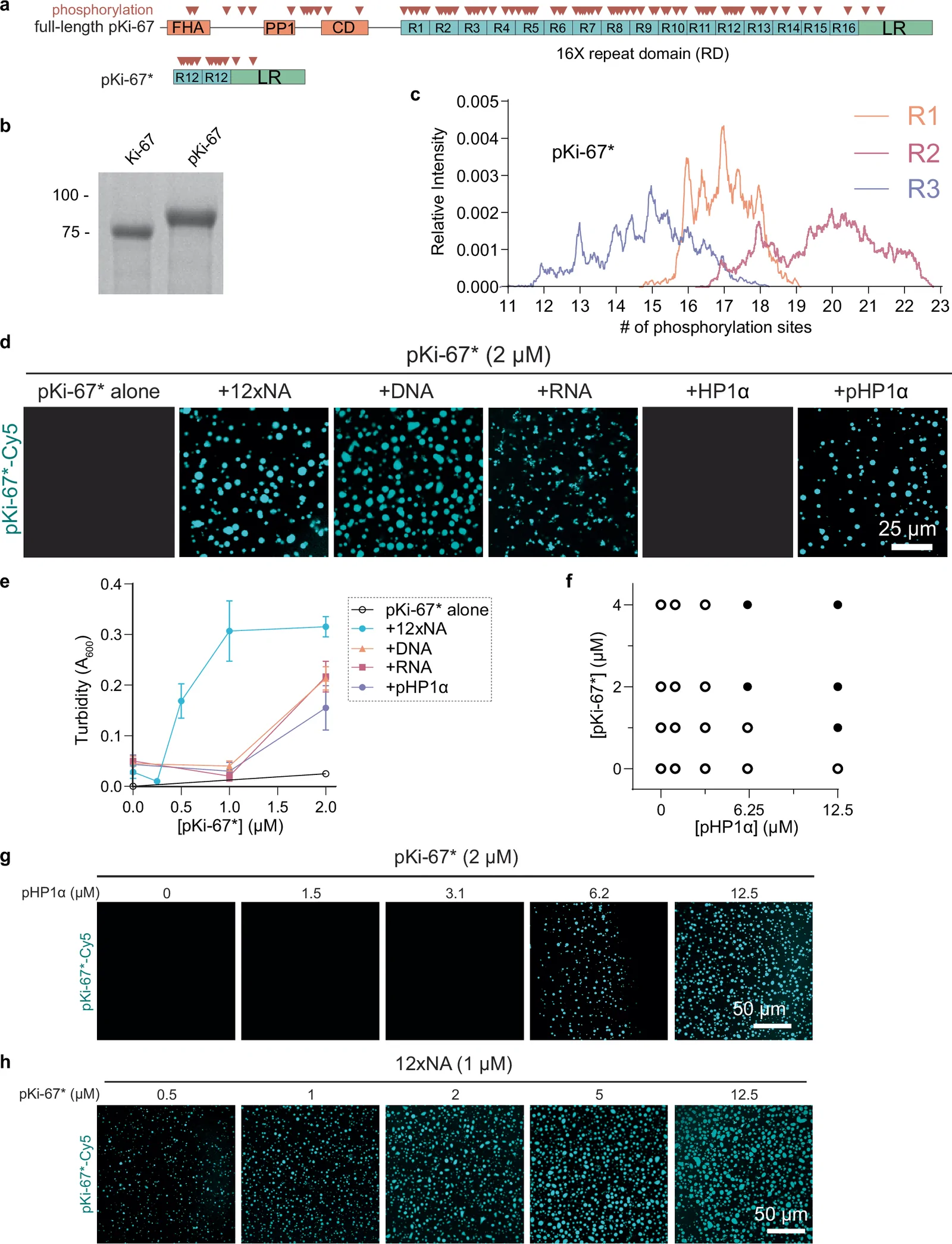
Phosphorylated Ki-67* forms condensates with its nuclear interactors. **a** Domain architectures of full-length Ki-67 and Ki-67* constructs with mitotic phosphorylation sites indicated. **b** SDS-PAGE analysis of Ki-67* phosphorylation (full gel image presented in Supplementary Fig. 10).** c** Relative abundance of the number of phosphates detected on phosphorylated Ki-67*. Individual mass spectrometry replicates are shown for independent phosphorylation reactions (R1-3 denote replicates). Relative intensity is calculated by dividing the raw intensity by the area under the mass spectrum curve as approximated by the trapezoidal rule, to correct for differences in sample amount. **d** Confocal microscopy images of droplets of 2 μM pKi-67* alone or with 40 nM 12xNA (0.5 μM equivalent of mononucleosomes), 75 ng/μL 2.1 kbp DNA (equivalent DNA concentration to 12xNA), 75 ng/μL RNA (extracted from nuclei isolated from P11 mouse cerebellum), 6.25 μM HP1α, and 6.25 μM pHP1α, visualized with 5% pKi-67*-Cy5 (scale bar = 50 μm). Studies were conducted in 20 mM Tris buffer, 75 mM KCl, 0.5 mM TCEP, pH 7.2 at 25 °C. Colocalization images are found in Supplementary Fig. 11. **e** Turbidity at A600 of pKi-67* with different nuclear components with variable pKi-67* concentration. The average of three biological replicates is shown (*n* = 3). Error bars represent standard deviation. Source data are provided as a source data file. **f** Phase diagram of pKi-67* liquid droplet formation at varying concentrations of pHP1α. Open dots indicate no phase separation, while filled dots indicate phase separation. Each condition was assessed at least twice by microscopy. **g** Confocal microscopy images of 2 μM pKi-67* with varying concentrations of pHP1α. Images are visualized with 5% pKi-67*-Cy5. **h** Confocal microscopy images of droplets of 80 nM 12xNA (1 μM equivalent of mononucleosomes) with variable pKi-67* concentrations, visualized with 5% pKi-67*-Cy5.

Previous phosphoproteomic analysis of the APC7-deficient brain established that Ki-67 accumulates in a phosphorylated state^30^. As Ki-67 is known to be phosphorylated during mitosis^53^, this observation, in combination with the fact that the Ki-67 mRNA was not overexpressed, is most compatible with a model in which Ki-67 fails to be degraded after mitotic exit in terminally differentiating neurons. Therefore, after observing phase separation of Ki-67* in vitro, we became interested in how phosphorylation of Ki-67* would change this behavior. On one hand, phosphorylation of Ki-67 has been suggested to promote phase separation after mitosis^39^ and to increase the phase separation propensity of the repeat regions in the presence of crowding agents^40^. On the other hand, phosphorylation can weaken the LRD’s interactions with DNA^16–18^. This makes predictions regarding its effects on Ki-67* phase separation with heterochromatin components somewhat challenging. More than 67 phosphorylation sites have been detected in mitotic Ki-67, nine of which reside in the R12 domain (Fig. 5a)^40,54,55^. After incubating Ki-67* with its kinase Cdk1, we confirmed phosphorylation of Ki-67* (pKi-67*) by an SDS-PAGE gel shift assay and intact mass spectrometry, with the latter assay demonstrating production of pKi-67* harboring between 12–22 phosphorylated sites (Fig. 5b, c and Supplementary Fig. 10).

Similarly to Ki-67*, pKi-67* could not form condensates by itself, but combining it with 12xNAs, DNA, RNA or pHP1α led to phase separation (Fig. 5d). pKi-67*-Cy5 colocalized with pHP1α-Cy3 and 12xNA-YOYO-1 within condensates (Supplementary Fig. 11a, b). Compared to Ki-67*, the same concentration of pKi-67* resulted in lower turbidity values when combined with 12xNAs, nucleic acids or pHP1α (Figs. 5e, 4c). Relative to Ki-67*, pKi-67* required an increase in the concentration of pHP1α (from 3 μM to 6 μM) necessary to trigger condensate formation (Figs. 5f, g, 4e, g). The behavior of Ki-67* and pKi-67* with respect to 12xNAs was similar (Figs. 5h, 4b), a finding that is likely explained by the fact that fewer phosphorylation sites reside within the chromatin-associating LRD (Fig. 5a), and the ability of the construct to form multivalent interactions both with the negatively charged DNA and the positively charged histone tails. These observations reveal that, despite phosphorylation significantly reducing the positive charge of Ki-67*, pKi-67* exhibits comparatively modest reduction in propensity to drive phase separation.

### Phosphorylation increases the internal mobility of Ki-67* within condensates

**Fig. 6:**
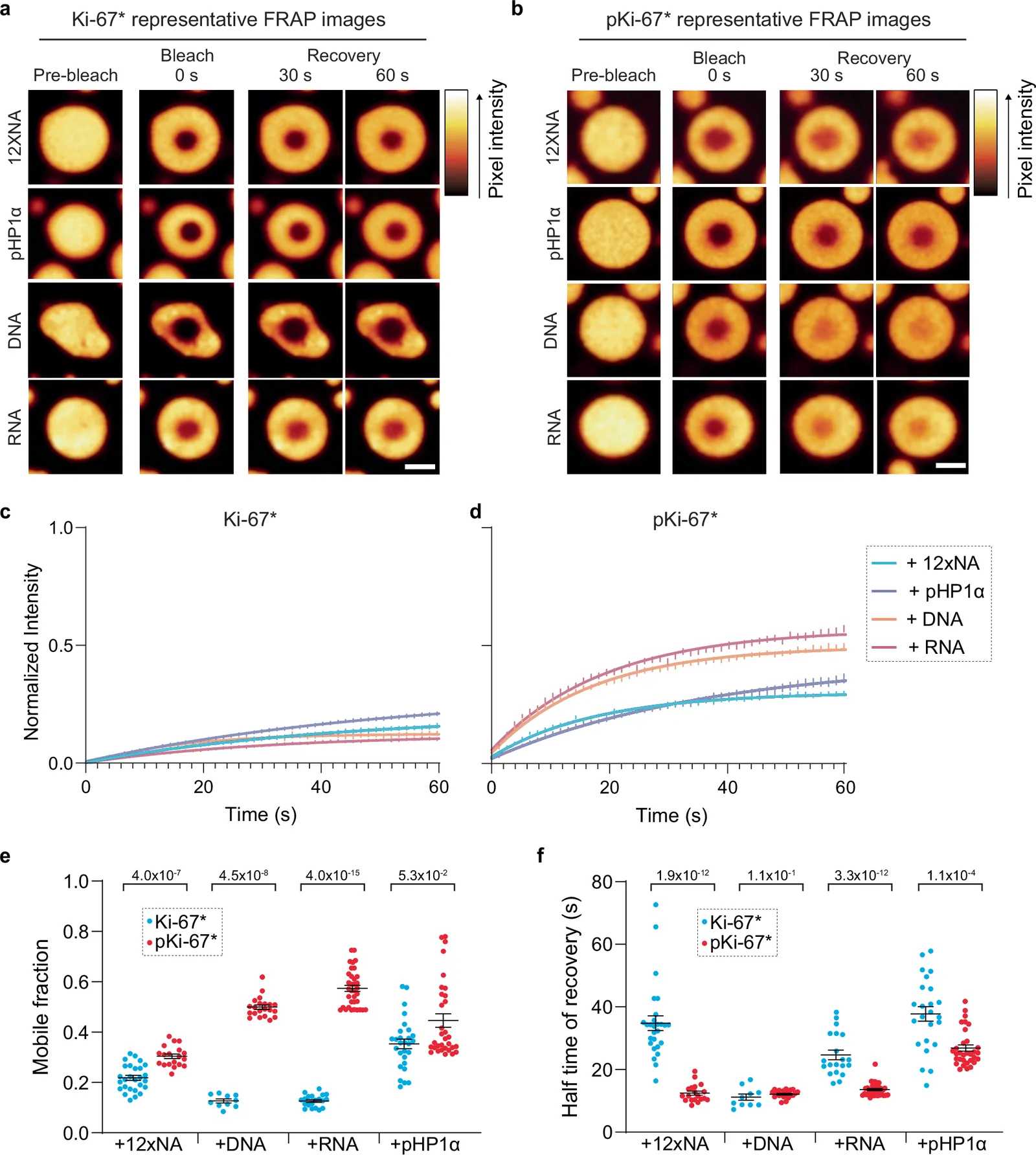
Ki-67* phosphorylation enhances its liquid-like condensate dynamics. **a** Representative pre-bleach, bleach and post-bleach fluorescence images for various Ki-67* droplet conditions, colored by pixel intensity. Scale bars, 5 μm.** b** Representative pre-bleach, bleach and post-bleach fluorescence images for various pKi-67* droplet conditions, colored by pixel intensity. Scale bars, 5 μm.** c** FRAP analysis of 12.5 μM Ki-67* droplets with different nuclear components. The best monoexponential fit curve and standard error of the mean are plotted for each data set (*n* = 27 droplets for Ki-67* with 12xNA, 10 with DNA, 20 with RNA and 29 with pHP1α). Quantification of maximum recovery (%) and half-life (s) is tabulated in Supplementary Table 1. Source data are provided as a source data file. **d** FRAP analysis of 12.5 μM pKi-67* droplets with different nuclear components. The best monoexponential fit curve and standard error of the mean are plotted for each data set (*n* = 19 droplets for pKi-67* with 12xNA, 21 with DNA, 35 with RNA and 32 with pHP1α). Quantification of maximum recovery (%) and half-life (s) is tabulated in Supplementary Table 1. Source data are provided as a source data file.** e** Comparison of the mobile fraction of Ki-67*/pKi-67* droplets with various nuclear interactors. Mobile fraction values were obtained from exponential fits to the traces. Each point represents the mobile fraction value for an individual droplet (*n* = 27 droplets for Ki-67* with 12xNA, 10 with DNA, 20 with RNA and 29 with pHP1α; *n* = 19 droplets for pKi-67* with 12xNA, 21 with DNA, 35 with RNA and 32 with pHP1α). Bars represent the mean and standard error of the point distribution. P-values by two-tailed Mann-Whitney U-test. Source data are provided as a source data file. **f** Comparison of half-time values (t_1/2_) of the fluorescence recovery of Ki-67*/pKi-67* droplets with various nuclear interactors. Half-time values were obtained from exponential fits to the traces. Each point represents the half-time value for an individual droplet (*n* = 27 droplets for Ki-67* with 12 x NA, 10 with DNA, 20 with RNA and 29 with pHP1α; *n* = 19 droplets for pKi-67* with 12xNA, 21 with DNA, 35 with RNA and 32 with pHP1α). Bars represent the mean and standard error of the point distribution. P-values by two-tailed Mann-Whitney U-test. Source data are provided as a source data file.

After the somewhat surprising observation that phosphorylation of Ki-67* has only a modest effect on its tendency to promote phase separation, we hypothesized that this post-translational modification could influence the biophysical properties of condensates through a distinct mechanism, for instance by altering its mobility and/or dynamics within condensates. Therefore, we performed fluorescence recovery after photobleaching (FRAP) experiments to compare the liquid-like behavior of condensates containing Ki-67* to those containing pKi-67* (Fig. 6a, b). We found that condensates bearing Ki-67* exhibited low mobility across condensates of distinct composition, with mobile fractions of 21.1% with 12xNAs, 12.4% with DNA, 12.3% with RNA, and 31.1% with pHP1α (Fig. 6c and Supplementary Table 1). Slow recovery could be explained by strong interactions between Ki-67* and these nuclear components, as previously reported for other DNA-binding proteins^56,57^.

FRAP studies of condensates containing pKi-67*, in contrast, demonstrated much greater internal mobility compared to Ki-67* (Fig. 6d). The mobile fraction of pKi-67* condensates increased to 30.2% when combined with 12xNAs, 49.8% with DNA, 57.0% with RNA and 43.6% with pHP1α (Fig. 6d, e and Supplementary Table 1). The largest differences between Ki-67* and pKi-67* occur when combined with DNA and RNA, suggesting that phosphorylation impacts Ki-67’s interaction with free nucleic acids more so than 12xNAs or pHP1α. In general, the half-time to recover fluorescence (t_½_) for pKi-67* compared with Ki-67* decreased accordingly, from 30.7 s to 12.3 s when combined with 12xNAs, 22.8 s to 13.3 s with RNA, and 37.8 s to 26.1 s with pHP1α (Fig. 6f and Supplementary Table 1). Collectively, these results demonstrate that phosphorylation enhances the mobility of Ki-67* in heterotypic condensates composed of relevant heterochromatin components.

### Phase separation is driven by electrostatic interactions between Ki-67* and pHP1α

As previously noted, Ki-67* and the repeat regions of Ki-67 in general contain alternating patterns of positively and negatively charged patches, which are modulated in the presence of phosphorylation^40^. It has been shown that such patterns are important for the formation of multivalent electrostatic interactions that drive phase separation^58,59^. On the other hand, previous studies have shown that the LRD can interact with the chromoshadow (CSD) domain of HP1α^19,20^. HP1α functions as a homodimer that is mediated through CSD-CSD interactions, which in turn leads to the formation of a new binding surface where HP1α can interact with a multitude of binding partners^21^. These contacts are typically mediated by specific binding motifs on the binding partner, the so-called PXVXL-like^60^ or HP1 access code (HAC) motifs^61^. The LRD of Ki-67 contains several possible such motifs, including the relatively hydrophobic PVVIM and AKVLM sequences. Therefore, we wondered how Ki-67* and HP1α might balance specific HAC contacts with multivalent electrostatic interactions in the context of phase separation. To investigate this question, we used nuclear magnetic resonance (NMR) spectroscopy, which provided information on the interactions of Ki-67* with the CSD-CSD HP1α homodimer, along with coarse-grained molecular dynamics (MD) simulations, which allowed us to assess the network of electrostatic interactions in the Ki-67-HP1α condensates.

**Fig. 7:**
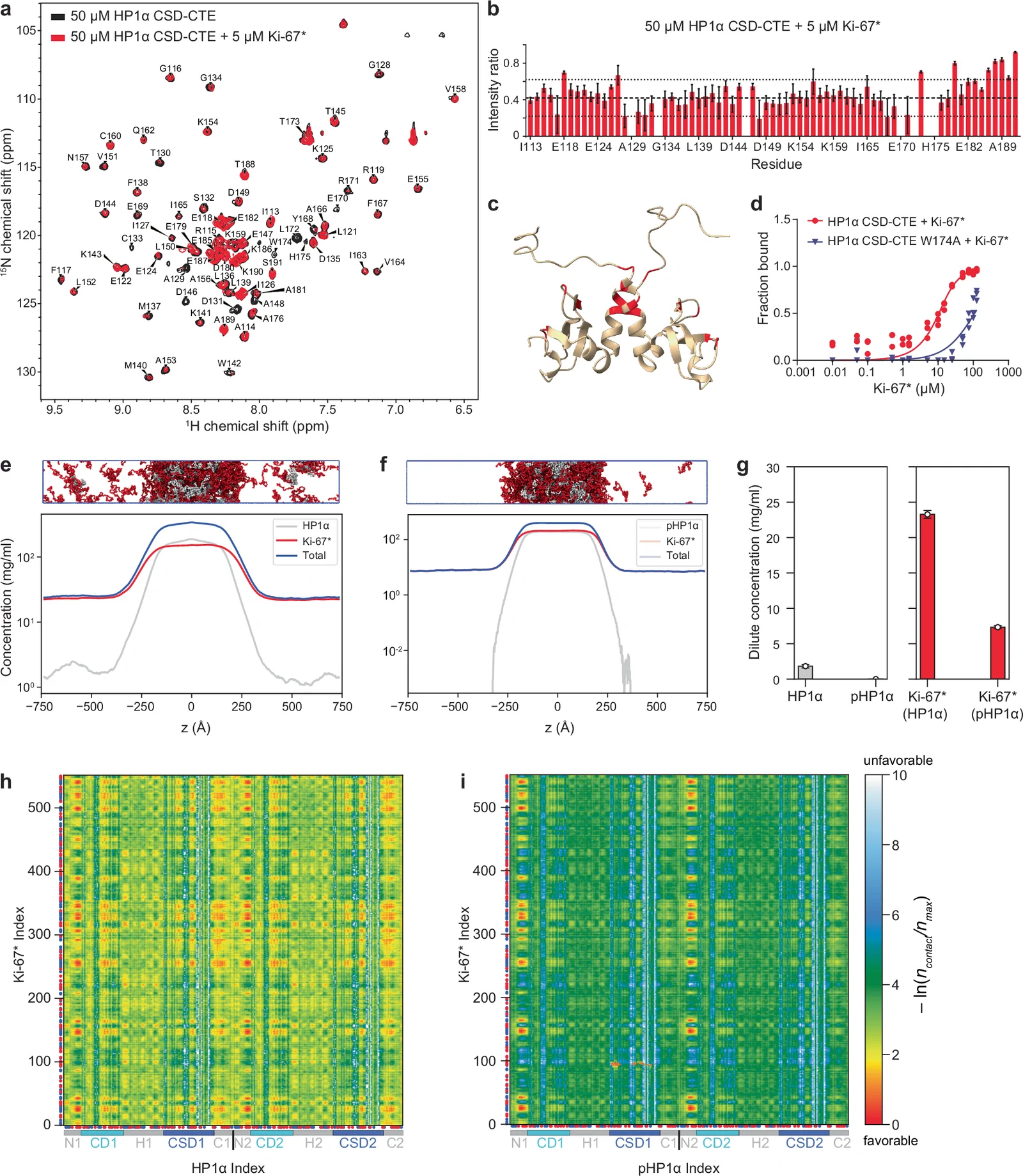
Ki-67* interacts with HP1α through a network of electrostatic interactions. **a** ^1^H-^15^N HSQC NMR experiments of 50 μM HP1α CSD-CTE with and without 5 μM Ki-67* (red and black spectra, respectively). **b** Analysis of the intensity ratios of the HP1α CSD-CTE cross-peaks in the presence and absence of 5 μM Ki-67*. Error bars are calculated based on the signal-to-noise ratio of the two compared spectra (see “Methods”). The black dashed line indicates the average intensity ratio when all peaks are considered, while the dotted lines represent one standard deviation above and below. Source data are provided as a source data file. **c** Model of the HP1α CSD-CTE dimer depicting the positions of residues that display intensity ratios below one standard deviation of the average (red), indicating potential interaction sites with Ki-67*. Source data are provided as a source data file. **d** Binding curves of 25 μM HP1α CSD-CTE (red) or CSD-CTE with the W174A mutation (blue) with varying concentrations of Ki-67*. The curves show data from three independent measurements.** e** Representative snapshot (top) and concentration profile (bottom) along the slab z-axis for coexistence simulations of HP1α mixed with Ki-67^*^, showing the partitioning of each component between the condensed and dilute phases. **f** Analogous data for pHP1α. **g** Dilute-phase concentrations of each component in mixtures of HP1α (or pHP1α) with Ki-67^*^. Error bars represent standard deviations from block averaging over five equal 800 ns blocks of a 5 μs trajectory after excluding the first 1 μs for equilibration. **h** Intermolecular contact map between Ki-67^*^ residues (y-axis) and HP1α residues (x-axis) within the condensates. Warmer colors indicate more favorable contacts. Blue and red dots on the axes denote the positions of positively (K/R) and negatively charged (D/E) residues, respectively. **i** Analogous dataset for pHP1α.

To perform NMR experiments, we isotopically labeled an HP1α construct consisting of the CSD-CTE portion of the sequence and capable of forming homodimers. This construct contains the relevant HAC binding surface and yields well-resolved ^1^H-^15^N heteronuclear single quantum coherence NMR spectra that allowed us to determine the specific HP1α residues involved in Ki-67* interactions (Fig. 7a). Titration with increasing concentrations of Ki-67* led to a global decline in cross-peak intensity, while for some residues (A129, C133, D146, A148, Y168, E170, L172, W174, and H175) the decrease in intensity was more substantial, indicating potential interaction hotspots on the CSD-CTE homodimer surface (Fig. 7b and Supplementary Fig. 12a–c). These residues are primarily concentrated in the HP1α region responsible for contacts with binding partners through their HAC motifs (Fig. 7c). In addition, we performed binding studies with a CSD-CTE W174A mutant (Fig. 7d), a construct that can still dimerize but displays substantially weaker binding to HAC motifs^62^. These experiments confirmed that indeed Ki-67* contacted HP1α at the interface responsible for HP1α binding partner interactions. Interestingly, however, deleting or mutating the two putative HAC motifs on Ki-67* did not significantly affect binding with the CSD-CTE homodimer (Supplementary Fig. 12d, e). These observations suggest that while Ki-67* contacts the HP1α CSD-CTE at a specific location, Ki-67* does not utilize a single HAC for this purpose, and most likely, there are several interaction regions that can compensate for each other.

To understand the multivalent interaction network between Ki-67* and HP1α/pHP1α in more detail, we turned to coarse-grained MD simulations based on the recently developed HPS-Urry model^63^. These simulations enable predictions regarding the relative phase separation propensity of different proteins and their variants, determination of protein concentrations in the dense and dilute phases, and analysis of protein-protein contacts within condensates^51^. For example, comparison of the phase separation propensity of Ki-67* with HP1α and pHP1α (Fig. 7e, f) revealed that HP1α phosphorylation significantly enhanced condensate formation, consistent with our experimental results. While both HP1α and pHP1α entered such condensates relatively efficiently, Ki-67* had a much stronger preference for pHP1α enriched condensates (Fig. 7g). The contact maps within these condensates revealed that HP1α and Ki-67* formed an extensive network of multivalent interactions mediated by multiple charged patches on both proteins (Fig. 7h). In pHP1α/Ki-67* condensates, on the other hand, the Ki-67* interactions focused on the phosphorylated N-terminal extension (NTE) of HP1α (Fig. 7i). These observations suggest that in the absence of HP1α phosphorylation, charged regions on both proteins compete for interactions, ultimately weakening phase separation propensity. NTE HP1α phosphorylation alleviates this competition by creating a dominant interaction hotspot that ultimately enhances condensate formation. Taken together, our NMR and MD simulation data indicate that while Ki-67* can contact HP1α at its specific CSD-CSD binding interface, phase separation between the two proteins is mediated by electrostatic interactions comprising multiple positively charged patches on Ki-67* and the negatively charged phosphorylated patch on the HP1α NTE.

### Ki-67* phase separates with HP1α/pHP1α and chromatin independently of histone modifications

Finally, we wondered how relevant histone PTMs may influence the ability of HP1α and Ki-67 to undergo phase separation. This included H3K9me3, a characteristic heterochromatin histone modification that recruits HP1α to nucleosomes. We were also keen to examine the mitosis-associated modification H3S10 phosphorylation (H3S10p). H3S10p accumulates in constitutive heterochromatin of APC7-deficient neurons as a result of impaired clearance of the APC substrate Chromosome Passenger Complex, whose kinase subunit Aurora B phosphorylates histone H3 to produce H3S10p^30^. Therefore, the enlarged chromocenters in *Anapc7*^-/-^ mice are expected to be enriched in both pKi-67 and H3S10p, suggesting that this modification may be relevant for the observed phenotype. On the other hand, H3S10p has a well-documented ability to disrupt HP1α binding to H3K9me3^64^ and to interfere with phase separation of the HP1α paralog HP1β in vitro^65^. Some studies propose that H3S10p and H3K9me3 constitute a phospho-methyl switch capable of displacing HP1α from chromatin during mitosis^64,66,67^, whereas other studies suggest that H3K9me3 and H3S10p can coexist during mitosis^64,68^. In light of this ambiguity, we opted to examine the phase separation of HP1α and Ki-67* in the presence of nucleosome arrays harboring either H3K9me3 or H3S10p alone as well as in combination. Arrays containing H3K9me3 were prepared by substituting a methyl-lysine analog at Lys9^69^, whereas phosphorylation of Ser10 was added in vitro by Aurora B Kinase (Supplementary Fig. 13).

We first confirmed that the modifications had the expected effects on HP1α and pHP1α phase separation. In particular, H3K9me3 enhanced the phase separation ability of both proteins, while 12xNAs harboring H3S10p reduced the turbidity of suspensions containing either HP1α or pHP1α relative to unmodified arrays (Supplementary Fig. 14). 12xNAs modified with both H3K9me3 and H3S10p exhibited intermediate behavior compared to arrays harboring each modification in isolation. The effect of H3S10p was less pronounced with HP1α than pHP1α, potentially resulting from the fact that HP1α binds H3K9me3 with less avidity^49^, instead associating with chromatin via non-specific interactions with DNA^48,51,70^.

**Fig. 8:**
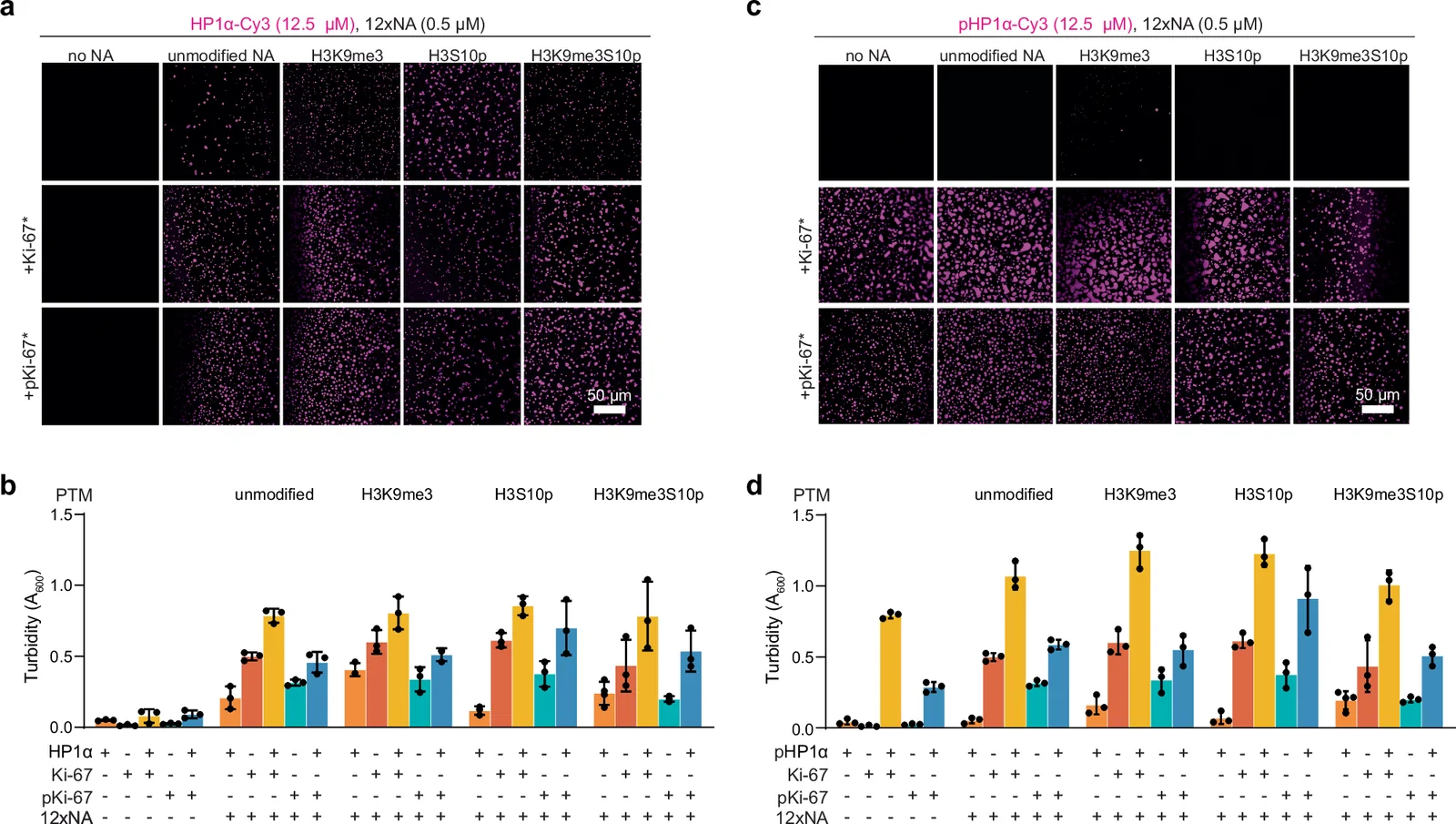
Influence of chromatin and Ki-67* PTMs on HP1α LLPS. **a** Confocal microscopy images of HP1α with variable 12xNA PTMs and Ki-67* or pKi-67*, visualized with 5% HP1α-Cy3. Colocalization images can be found in Supplementary Fig. 15 and Supplementary Fig. 16. **b** Turbidity at A600 of Ki-67*, HP1α, and 12xNA combinations with different PTMs. Standard concentrations are 12.5 μM HP1α, 2 μM Ki-67* or pKi-67*, and 40 nM 12xNA. Individual points represent three biological replicates (*n* = 3) except the HP1α + 12xNA H3K9me3S10p condition, where four biological replicates were obtained (*n* = 4). Error bars represent standard deviation. Source data are provided as a source data file. **c** Confocal microscopy images of pHP1α with variable 12xNA PTMs and Ki-67* or pKi-67*, visualized with 5% pHP1α-Cy3. Colocalization images can be found in Supplementary Fig. 15 and Supplementary Fig. 16. **d** Turbidity at A600 of Ki-67*, pHP1α, and 12xNA combinations with different PTMs. Standard concentrations are 12.5 μM pHP1α, 2 μM Ki-67* or pKi-67*, and 40 nM 12xNA. Individual points represent three biological replicates (*n* = 3) except the pHP1α + 12xNA H3K9me3S10p condition, where four biological replicates were obtained (*n* = 4). Source data are provided as a source data file.

We next examined how Ki-67* modulated phase separation of HP1α or pHP1α in the presence of 12xNAs and different PTMs (Fig. 8). Even though Ki-67* could not form condensates with HP1α at the concentrations used in Fig. 4a, the inclusion of unmodified 12xNA led to robust phase separation (Fig. 8a, b). The addition of H3K9me3, H3S10p or H3K9me3S10p on 12xNAs had no significant effect on the turbidity of the resulting suspensions, implying that phase separation was insensitive to histone PTMs (Fig. 8b). This was also the case in samples that contained only Ki-67* and unmodified/modified 12xNAs. These observations suggest that Ki-67* can utilize its alternating charged patterns to build robust interaction networks with nucleosome arrays, independent of different PTMs on the H3 tail. The presence of HP1α adds on to these interaction networks, leading to a moderate increase in turbidity for all PTM combinations.

We next assessed the ability of phosphorylation, whether on HP1α or Ki-67*, to influence phase separation in the context of 12xNAs. While pHP1α had lower phase separation propensity with 12xNAs compared to HP1α (Fig. 8c), the addition of Ki-67* significantly increased the turbidity of the samples (Fig. 8d) and surpassed the turbidity measured for HP1α, Ki-67* and 12xNAs (Fig. 8b). Thus, samples containing pHP1α, non-phosphorylated Ki-67* and 12xNAs displayed the highest propensity for phase separation, which was again largely insensitive to the presence of various histone modifications (Fig. 8d). We attribute this behavior to the strong electrostatic network between Ki-67* and the phosphorylated HP1α NTE as seen in our MD simulations (Fig. 7i), with 12xNAs bringing in additional contacts that can moderately enhance phase separation. On the other hand, phosphorylation of Ki-67* uniformly reduced turbidity in almost all samples, except for the combinations of HP1α/pHP1α, pKi-67* and H3S10p arrays. We interpret these datapoints with a degree of caution, as the use of recombinant Aurora B to phosphorylate H3 produces heterogeneously phosphorylated samples. We also cannot exclude the possibility that the presence of H3S10p refocuses the electrostatic interactions between the components, strengthening phase separation, similar to the effect of NTE phosphorylation on HP1α (Fig. 7i). Finally, our imaging experiments showed that all components, Ki-67*/pKi-67*, HP1α/pHP1α, and 12xNA, co-localized within the condensates (Supplementary Figs. 15, 16).

Taken together, our results suggest that Ki-67* can increase the phase separation propensity of HP1α and nucleosome arrays and that this process is tolerant to different histone PTMs. Phosphorylation of HP1α enhances this process further, while phosphorylation on Ki-67* reduces but does not prevent phase separation. These observations have important implications for the potential interactions that may be present in heterochromatin environments in neurons from patients with the *ANAPC7* neurodevelopmental syndrome (elevated pKi-67, H3S10p/H3K9me3-modified nucleosomes, pHP1α/HP1α) compared to the components representative of typically developing neurons, whose heterochromatin instead possesses pHP1α/ HP1α and H3K9me3 but is not enriched in Ki-67/pKi-67.

## Discussion

Ki-67 has a well-established function in mitosis where it forms a protective surfactant layer on the chromosomes to prevent their intermingling^11,13^. Upon exit from mitosis, Ki-67 is involved in chromosome condensation, a process that involves the formation of a liquid-like coat on the chromosome surfaces that promotes their clustering^14,39^. After mitosis, Ki-67 is rapidly degraded by the APC^31,71^, however, some Ki-67 remains associated with heterochromatin, where it is believed to play a role in the organization of this compartment^31,72,73^. Our study investigates the heterochromatic roles of Ki-67 in differentiating neurons, a context relevant for typical neurodevelopment and the *ANAPC7* neurodevelopmental syndrome, where Ki-67 is not degraded properly after mitosis^7^. Using genetic mouse models, we examine the effects of depletion or overabundance of Ki-67 on heterochromatin organization in mouse granule neurons, while our in vitro biophysical experiments and computational studies allow us to propose a mechanistic view of how Ki-67 influences heterochromatic environments.

To understand the effects of Ki-67 on heterochromatin compartments in vivo and in neurons, we relied on our previously published *Anapc7*^*-/-*^ mouse model^7,30^, in which Ki-67 is aberrantly elevated in the nucleus due to inadequate degradation, and our newly developed *Mki67*^*-/-*^ model in which Ki-67 is completely eliminated. Overabundance of Ki-67 results in enlarged chromocenters, while loss of Ki-67 leads to a more fragmented heterochromatin phenotype, where the chromocenters are smaller and more numerous. Although these effects are relatively modest in scale, they are consistent with previous observations in various other cell models. For example, knockdown of Ki-67 in HeLa and U2OS cells caused a reduction in perinucleolar heterochromatin as observed by DAPI staining, while overexpression of Ki-67 led to ectopic heterochromatin formation^31^. Experiments in hTERT-RPE1, HCT116, and K562 human cell lines, on the other hand, have shown that Ki-67 regulates the distribution of heterochromatin between the perinucleolar and the nuclear lamina compartments^73^. Finally, a recent study suggests that Ki-67 degradation in HCT116 cells leads to decompaction of centromeric satellite regions both in mitosis and G1^74^. Collectively, these results reinforce previous findings that post-mitotic Ki-67 plays a role in global heterochromatin organization, including in granule neurons.

While the picture of how Ki-67 affects global heterochromatin phenotypes is relatively consistent, Ki-67’s influence on the underlying epigenetic landscape seems to be more complex. The presence of Ki-67 within heterochromatin has been correlated with H3K9me3 enrichment^31^, while reduction of Ki-67 levels has been observed to cause the redistribution of H3K9me3 without changing its levels^73^. The effects appear to be cell-type specific. In our previous study of the *Anapc7*^*-/-*^ mouse model, where Ki-67 and the kinase Aurora B are both overabundant, we detected elevated levels of H3S10p in neuronal nuclei but no substantial changes in H3K9me3 levels^30^. Nevertheless, the abundance of major γ-satellite RNA was reduced in the developing cerebellum of *Anapc7*^*-/-*^ mice^7^. Depletion of Ki-67 in our *Mki67*^*-/-*^ model also did not cause substantial changes in heterochromatin and euchromatin marks, and we did not observe any significant effects on neuronal transcription. While this result contrasts with previous transcriptomic studies, this difference could result from distinct cellular contexts: euploid mouse neurons versus karyotypically abnormal carcinoma and sarcoma cell lines^31^. Taken together, our in vivo observations in the brain suggest that overabundant Ki-67 may be involved in silencing mechanisms that are not necessarily H3K9me3 dependent, while loss of Ki-67 may be compensated by other factors that help maintain the necessary levels of silencing despite the more fragmented heterochromatin phenotype.

Having observed intriguing changes in heterochromatin organization upon Ki-67 overabundance or depletion, we wondered about the molecular mechanisms that may be mediating those effects. Our results in mouse neurons confirm co-localization of Ki-67 and HP1α, while experiments in N2a cells indicate that the LRD is sufficient for localization into mouse chromocenters. Previous literature has suggested that this domain can interact with the CSD domains in HP1 proteins^19,20^. Our in vitro binding assays and NMR experiments confirmed this interaction and highlighted a groove on the CSD-CTE dimer interface as the binding site on HP1α. While this HP1α site is known to bind the so-called PXVXL or HAC motifs on multiple binding partners^60,61^, intriguingly, the LRD does not appear to have a classical binding motif. Instead, our computational studies suggest that the sequence patterning in the LRD (and the R12 repeat) promotes multivalent electrostatic interactions with HP1α through multiple charged patches.

Our experiments in live mouse neurons suggest that Ki-67-enriched nuclear bodies display dynamic properties consistent with phase separation, while our minimal recombinant Ki-67* construct can form condensates in vitro with nucleic acids and nucleosome arrays. As a binding partner, Ki-67* also significantly enhances the phase separation propensity of pHP1α, which is essentially always present in cells alongside its dephosphorylated counterpart^49^. While pHP1α typically undergoes phase separation on its own at concentrations ~100 μM and ~ 12.5 μM in the presence of H3K9me3 arrays (Supplementary Fig. 14), Ki-67* reduces this threshold concentration to ~ 3 μM (Fig. 4e, g). Interestingly, while Ki-67* does not enhance the phase separation propensity of unmodified HP1α when the two proteins are combined, Ki-67* readily partitions into and enhances HP1α condensation in the presence of nucleosome arrays (Fig. 8a, b). Our simulations (Fig. 7h, i) suggest that this difference arises due to refocusing of the electrostatic interactions between the highly positively charged Ki-67* and pHP1α, which contains a negatively charged patch on its N-terminus. These differences become diminished in the presence of nucleosome arrays (Fig. 8b, d), which provide a platform for a complex network of electrostatic interactions between all components, in addition to the binding interactions between HP1α and the LRD of Ki-67*, and between HP1α and H3K9me3 on nucleosomes. The encoded multivalency also allows the phase separation process to be remarkably robust with respect to histone PTMs, and the presence of H3S10p, which disrupts the specific interaction between HP1α and H3K9me3 but does not interfere with phase separation. Taken together, these observations suggest that Ki-67* enhances the phase separation of heterochromatin components, and that the phase separation process is resilient even in the presence of aberrant modifications such as H3S10p, which is enriched within heterochromatin of APC7-deficient neurons during neurodevelopment^30^.

Previous work has also suggested that phosphorylation of Ki-67 dramatically alters its biophysical properties, acting as an on-off switch to regulate chromosome clustering and Ki-67 condensation^39^. The effects of phosphorylating Ki-67* are more modest, as pKi-67* retains its ability to undergo phase separation with nuclear components, albeit at slightly higher concentrations. Instead, Ki-67* phosphorylation primarily impacts its mobility within condensates in vitro, a finding that is consistent with previous reports suggesting that Ki-67 has the highest mobility, as measured by FRAP, during stages of the cell cycle when it is likely to be phosphorylated^18^. Because Ki-67 accumulates as a phosphoprotein in developing APC7-deficient neurons^30^, these observations also reinforce the idea that pKi-67 may influence the dynamics and material properties of heterochromatin domains.

Taken together, our genetic mouse models, cellular experiments and in vitro biophysical work suggest that Ki-67 plays a role in heterochromatin organization in developing neurons and that this function may be mediated through a phase separation process that involves interactions between the Ki-67 LRD and HP1α. This phase separation process is likely distinct from the liquid coat formed by phosphorylated Ki-67 at the end of mitosis, which instead relies on its N-terminal charged patch^39^. While the role of phase separation in heterochromatin formation and organization has been actively debated in the literature^29,75–79^, our model is supported by the observed changes in chromocenter number and size as a function of Ki-67 depletion or overabundance, the high degree of co-localization between Ki-67 and H3K9me3^30^ and HP1α in cells, the ability of Ki-67 nuclear bodies to undergo fusion in live cell imaging of neurons, and the robust phase separation observed between Ki-67* and various heterochromatin components in vitro. The increase in chromocenter size as a function of Ki-67 concentration (comparing control vs Ki-67 enriched *Anapc7*^*-/-*^ neurons), in particular, supports a “concentration buffering” mechanism consistent with phase separation, rather than the “size buffering mechanism” proposed for the heterochromatin protein MeCP2, where the chromocenter size and number did not change as the expression levels of MeCP2 were modulated^77^. Nevertheless, we acknowledge that alternative models may also be able to explain our observations and that more work is necessary to unequivocally support the Ki-67 heterochromatin phase separation mechanism in mouse cells (e.g., FRAP experiments, which we were unable to perform due to the low sensitivity of RFP-tagged Ki-67 in live neurons) and human neurons, which differ substantially in heterochromatin organization and localization^77^. We also cannot rule out the involvement of other heterochromatin components, including proteins and RNA that have been shown to modulate phase separation^51,80,81^. Finally, while our in vitro work focused on a minimal construct of Ki-67 rather than the full protein, it is possible that other Ki-67 domains also contribute to its heterochromatin functions, even if they are not essential for localization to these compartments. It will also be important to further investigate the exact nature of Ki-67 and HP1α interactions, which exploit the binding interface of HP1α but do not appear to rely on a classical HAC/PXVXL-binding motif in the LRD.

In summary, our study bolsters the notion that the *ANAPC7* neurodevelopmental syndrome constitutes a bona fide member of the large and growing class of neurodevelopmental disorders that stem from mutations in genes whose encoded proteins control the properties and behavior of chromatin. Our examination of the role of Ki-67 in heterochromatin organization using in vitro and in vivo assays adds to the body of evidence indicating that Ki-67 can influence the biophysical properties of heterochromatin in collaboration with other important members of heterochromatin environments, such as HP1α. Therefore, our findings carry implications for understanding of the principles that govern heterochromatin formation during both typical neurodevelopment and in the pathogenesis of neurodevelopmental disorders.

## Methods

### Mice

Animals were cared for in accordance with NIH guidelines. All experimental methods were approved by the UCSD Institutional Committee on the Use and Care of Animals under protocol S20121. Animals were housed in a 12:12 light:dark cycle, ambient temperature and humidity. In general, we employed trio breeding. Pups were genotyped at P4-P5 by tail biopsy and weaned into sex-matched cages at P21 to be bred at 7-8 weeks. Animals were allocated to experimental versus control groups based on genotype. Sex matched-littermate controls were used in all experiments, and the age at which animals were used is reported in the figure legends. *ANAPC7-*mutant mice were generated as previously described^7^.

Embryonic stem (ES) cells on the JM8A3.N1 background (RRID:CVCL_J960, a specific sub-line of C57BL/6 N that have been engineered to correct the non-agouti mutation present in this mouse strain), which harbored the targeted *Mki67*^tm1a(EUCOMM)Wtsi^ allele were acquired from the European Mouse Mutant Cell Repository (EuMMCR). After blastocyst injection and transplantation into pseudopregnant females, chimeric male founders capable of germline transmission of the mutant allele were bred with female C57BL/6 mice expressing cre recombinase under the control of the Sox2 promoter (Jax #008454) for removal of floxed exon 4 and the neomycin resistance cassette. Heterozygous *Mki67*^+/-^ mice were backcrossed onto the Jackson labs C57BL/6 strain for 5 generations. For experiments with *Mki67*^-/-^ mice, sex-matched littermates of the genotypes *Mki67*^+/-^ and *Mki67*^+/+^ mice served as controls.

### Nuclear isolation

Mice were anesthetized with isoflurane before decapitation and extraction of the cerebellum. Each cerebellum was homogenized with a Dounce A pestle (Wheaton) in 2 mL lysis buffer (Nuclei EZ Lysis Buffer (Sigma) supplemented with 1X Halt combined protease and phosphatase inhibitor cocktail (Thermo Fisher Scientific), 10 mM sodium butyrate (Sigma)). This suspension was incubated on ice before centrifugation at 500 x *g* for 5 min, with acceleration and deceleration on the lowest setting, at 4 °C. The supernatant was aspirated, and nuclei were resuspended in lysis buffer and incubated on ice for an additional 5 min prior to centrifugation. Nuclei were resuspended in wash buffer (20 mM HEPES pH 7.5, 150 mM NaCl, 0.5 mM spermidine, supplemented with Roche complete EDTA-free protease inhibitor tablet). Pelleted nuclei were resuspended twice in wash buffer and subjected to centrifugation at 500 x *g* for 5 min. Nuclei were resuspended in 1 mL, filtered by gravity through a 40 µm mesh, and counted with a Countess II automated cell counter (Invitrogen).

### CUT&RUN

Between 200,000–500,000 nuclei were used in each CUT&RUN experiment. Nuclei were bound to Concanavalin A-coated magnetic beads (EpiCypher). Following bead binding, the supernatant was discarded, and the bead-nuclei slurry was resuspended in 75 µL of antibody buffer (wash buffer supplemented with 0.005% digitonin (Promega) and 2 mM EDTA) containing primary antibody for H3K4me3 (9751S, CST), H3K27ac (39034, Active Motif), H3K9me3 (39766, Active Motif), or H3K27me3 (9733, CST) at a concentration of 1:75. Antibody incubation was carried out overnight at 4 °C on a nutator. Samples were washed with cold cell permeabilization buffer (wash buffer with 0.005% digitonin) to remove unbound antibody, and then incubated with 1X Protein A/G-MNase (EpiCypher) in cell permeabilization buffer for 15 minutes at room temperature on a shaker. Samples were washed with cold cell permeabilization buffer and then resuspended in 50 µL cell permeabilization buffer containing 2 mM CaCl_2_. Samples were digested for 2 hr at 4 °C on a nutator. The digestion reactions were quenched with 33 µL of STOP buffer (340 mM NaCl, 20 mM EDTA pH 8, 4 mM EGTA pH 7.7, RNase A 0.05 mg/mL (Promega), Glycogen 0.05 mg/mL (Invitrogen)). Samples were then incubated for 10 min at 37 °C to release digested chromatin fragments. The supernatant containing CUT&RUN fragments was transferred to fresh tubes, and DNA was purified via column purification.

### CUT&RUN library preparation and sequencing

Library preparation for CUT&RUN experiments was performed according to the manufacturer’s specifications using SPRI-selected beads (Beckman). The concentration of DNA yielded from CUT&RUN was measured on a Qubit device, and between 25 and 50 ng of input DNA was used for library preparation, with the amount of input DNA kept constant for all samples using the same antibody. 14 PCR cycles were used in the final amplification with Illumina-compatible adapters, universal i5 primer and barcoded i7 primers (NEB). After measuring the concentration of the amplified product on a Nanodrop device (Thermo Scientific), all samples were diluted to the same concentration for a given antibody, ranging from 10 ng/uL to 40 ng/uL. Tapestation was performed to determine the precise concentration and fragment distribution range and to ensure there were no significant amounts of adapter dimers. Samples were pooled for sequencing 100 bp reads in paired-end configuration on an Illumina NovaSeq X Plus platform at the UCSD Institute for Genomic Medicine. We aimed for > 25 million reads for all samples except for H3K4me3, for which 15 million reads were adequate given the narrow distribution of this histone modification.

### CUT&RUN data analysis

Compressed sequenced reads in fastq.gz format were first trimmed using Trimmomatic v.0.39^82^. We then aligned samples to the mm10 genome using Bowtie2 v.2.3.4.1^83^ and sorted BAM files, removing unmapped fragments using SAMtools v.1.14^84^. During alignment, we set a minimum Phred score of 33 and employed the --dovetail option. We then converted alignment files to.bigwig files using the bamCoverage tool in Deeptools v.3.5.0^85^ with 50 bp bins. Duplicate reads were not removed. Normalization was performed as previously described^86^. Bigwig files were examined in Integrated Genome Viewer and visualized using KaryoplotR v.1.22.0^87^.

### RNA isolation, sequencing, and analysis

Mouse cerebellar nuclei were isolated using the same nuclear isolation protocol for CUT&RUN. Isolated nuclei were homogenized in Trizol (Invitrogen). Total nuclear RNA was isolated using the column-based Qiagen RNeasy kit. For in vitro RNA experiments, total RNA samples were eluted in 20 mM Tris, 10 mM KCl, pH 7.8. Library prep was performed using 1 μg of input RNA with the Illumina Stranded Total RNA Prep Ligation with Ribo-Zero Plus kit. After depletion of rRNA and cDNA synthesis, the DNA was quantitated using Qubit and equalized across samples. Illumina-compatible dual index primers were used during 15 PCR cycles. The prepared libraries were subsequently sequenced using the Illumina NovaSeq X Plus platform to generate 100 bp paired-end reads. Each sample was sequenced to a depth of at least 20 million reads. RNAseq libraries were aligned using STAR v.2.7.9a^88^. Gene expression was calculated by featureCounts v.1.6.0^89^ for paired reads. Alignment files were converted to bigwig format using deepTools bamCoverage v.3.5.0. DESeq2 v.1.44.0^90^ was then used to generate the normalized counts metric.

Assessment of the abundance of transcripts derived from major repeats was performed as previously described^7^. After isolating RNA, we treated the RNA with DNase I (NEB) according to the manufacturer’s protocol. We generated cDNA using Bio-Rad iScript without polyA selection and included the essential no RT control in our experiment. The abundance of each transcript was normalized to GAPDH, and we subtracted the signal observed under no RT conditions from the amount of transcript detected in the RT condition to compensate for the small amount of DNA remaining after DNase treatment.

### Cerebellar granule neuronal culture

Cultures of mouse primary cerebellar granule neurons were generated from P7 mice as previously described^32^. For imaging experiments, primary granule neurons were plated onto acid-treated, poly-L-ornithine coated optical cover glass in 24-well dishes with 1,000,000 cells per well.

### Immunocytochemistry

Cells grown on optical cover glass were fixed in 4% paraformaldehyde for 15 min at 25 °C. All fixed cells were washed twice with PBS solution after fixation. Permeabilization was performed using a 0.3% PBS-Triton solution for 30 min followed by blocking in 5% normal goat serum for 1 hr. Cells were incubated with the antibody for Ki-67 (1:500, 14-5698-82, Invitrogen) overnight at 4 °C. Cells were washed before applying fluorophore-labeled secondary antibodies and Hoechst (1:1000) for 1 hr at 25 °C, then washed again and mounted.

For co-labeling of Ki-67 with HP1α (1:500, 2616, Cell Signaling technology), cells were fixed and permeabilized in ice-cold 1:1 methanol:acetone before drying for 5 min at room temperature and rehydrating with PBS. Blocking and subsequent steps were carried out as above.

### Immunofluorescence in tissue

P8 mice were decapitated before brain dissection and immersion fixation of the whole brain in neutral-buffered formalin overnight at 4 °C. Brain tissue was then placed in 30% sucrose in PBS until it sank, rocked in equal parts Optimal Cutting Temperature (OCT) & 30% sucrose until homogeneous, and placed in pure OCT overnight. Brains were sagitally cut, flash frozen on dry ice and then sectioned at 20 μm in a Leica cryostat. Slices were washed twice with PBS and permeabilized with a 0.3% PBS-Triton solution for 45 min, followed by blocking in 5% normal goat serum for 1 hr. Calbindin antibody binding (1:800, 13176, CST) and secondary antibody binding was performed as described for cells.

### Confocal imaging of cell cultures

For cell imaging, we primarily used the AXR system at the UCSD Nikon Imaging Center, built upon the Eclipse Ti2-E body. Two-color images were acquired in image scanning super resolution mode with the NSPARC detector in resonant mode. Using the Plan Apo ʎ 60x NA 1.40 oil WD 130 µm objective, we acquired 0.25 μm Z-stacks over the entire thickness of imaged cells. For CGN images, Z stacks were flattened by making maximum intensity Z-projections in ImageJ. To ensure there was no bias in the choice of individual fields for imaging, each field was chosen based exclusively upon the Hoechst channel, with the microscopist agnostic to the signal in other channels. For imaging of *Anapc7*-mutant cells, we used the Zeiss 880 LSM2 microscope equipped with an Airyscan hexagonal detector array at the UCSD Microscopy Core for near super-resolution confocal microscopy. Primary cultures of cerebellar granule neurons were fixed and subjected to immunostaining as described above at DIV2. We used the 63x objective to acquire 0.16 μm optical sections prior to Airyscan post-processing with default settings.

### Image analysis – chromocenters

Raw images of primary cerebellar granule neurons were acquired as described above and processed in ImageJ (v.2.9.0). Maximum intensity Z projections were generated for every image. The Hoechst channel was used to define regions of interest (nuclei), and binaries were manually generated within these boundaries. During analysis of cultured neurons, apoptotic cells or cells that did not display cerebellar granule neuron morphology were manually excluded. Rolling Ball background subtraction was applied to the Hoechst signal, followed by a threshold to generate the binaries for chromocenters. The Analyze Particles feature was utilized to extract chromocenter areas, numbers, and the percentage of space occupied within each nucleus.

### Image analysis – subcellular correlation

Raw images of N2a cells were acquired as described above and processed in the NIS elements software (v.5.30.1). The Hoechst channel was used to define regions of interest (nuclei), and 3D binaries were generated within these boundaries. The mean object intensity for each construct was then measured within these binaries. After generating the binaries on nuclei, the Pearson R Colocalization feature in NIS Elements was used to compute the Pearson R value on a pixel-by-pixel basis within a region of interest, in this case, a single nucleus, generating the correlation coefficient between the Hoechst and construct signals.

### Cloning

The FHA region, the N-terminal region, the charged patch segment, the R12_2_-LRD, and the R12_2_ regions of the human Ki-67 coding sequence were ordered as synthetic DNA molecules from Twist Biosciences and cloned into a CMV-mCherry vector by Gibson assembly using BamHI and HindIII sites. The LR region was PCR amplified from the R12_2_-LRD mCherry plasmid and cloned into a CMV-mCherry vector. Fragments were designed to be expressed as N-terminal mCherry fusion proteins.

### Cell culture and transfection

N2a (Neuro2a) cells (authenticated by manufacturer, ATCC: CCL-131) were maintained in Dulbecco’s modified Eagle medium (Gibco) supplemented with 10% fetal bovine serum (Gibco) plus 1% (v/v) penicillin-streptomycin (Gibco), and grown at 37 °C in 5% CO_2_. Cells were split 1:6 every 3–4 days. 50,000 cells were plated onto sterile, acid-treated 13 mm coverslips and grown for 8–16 hr before transfection using jetPrime (Polyplus). For each coverslip, 0.5 µg of DNA was used to transfect at a 1:2 DNA/jetPRIME ratio. Cells were then grown for an additional 16–24 hr before fixation.

### Ki-67* construct expression and purification

All Ki-67* constructs were expressed in BL21 (DE3) Rosetta *Escherichia coli* cells in a pET-2B-T vector with a His-tag followed by a TEV site. Cells were grown in Luria Bertani media with ampicillin at 37 °C, induced at an OD_600_ of 0.6-0.8 using 0.5 mM isopropyl-β-D-thiogalactoside (IPTG), and harvested after 3 hr. The bacterial pellet was resuspended in lysis buffer (50 mM Tris-HCl, 500 mM KCl, 0.5 mM tris(2-carboxyethyl) phosphine (TCEP), 10 mM imidazole, pH 8 at 4 °C, supplemented with Roche EDTA-free Protease Inhibitor Cocktail tablets. Cells were lysed by sonication, and the soluble fraction was collected by centrifugation. The supernatant was then incubated with Ni-NTA resin (5 mL resin per 1 L culture) with rotation at 4 °C for 1 hr and the lysate-resin was loaded onto a Bio-Rad Econo-Column and washed with wash buffer (50 mM Tris-HCl, 500 mM KCl, 0.5 mM TCEP, 10 mM imidazole, pH 7.8 at 4 °C), and then eluted with wash buffer containing 400 mM imidazole. The eluate was incubated with TEV-protease during dialysis into imidazole-free 20 mM HEPES, 50 mM KCl, 0.5 mM TCEP, pH 7.2 buffer overnight at 4 °C. Then, the TEV that precipitated overnight was removed by centrifugation, and the sample was loaded onto a HiTrap SP HP cation exchange column (5 mL) (Cytiva), and eluted using a linear gradient from 50 mM to 1 M KCl over 1 hr. Pure fractions were then dialyzed into 20 mM Tris-HCl, 50 mM KCl, pH 7.8 at 4 °C, 0.5 mM TCEP, concentrated to ~ 100–200 μM, aliquoted, flash frozen, and stored at − 80 °C until use. The isolated protein was characterized by sodium dodecyl sulfate–polyacrylamide gel electrophoresis (SDS-PAGE), reverse-phase high-performance liquid chromatography (RP-HPLC), and mass spectrometry (MS). Since Ki-67* constructs have an extinction coefficient of 0 M^−1^cm^−1^ at 280 nm due to the lack of tryptophan residues, protein concentration was determined using a Thermo Scientific Pierce BCA Protein Assay Kit.

### Histone expression and purification

Histones were prepared according to an established protocol^91^. Briefly, H2A, H2B, H3, and H4 were separately expressed in BL21(DE3) Rosetta *Escherichia coli* cells in Luria Bertani broth at 37 °C to an OD_600_ of 0.6–0.8 and then induced with 0.5 mM IPTG. The cells were then harvested by centrifugation after 2 hr and resuspended in lysis buffer (20 mM Tris-HCl, 200 mM NaCl, 1 mM EDTA, 1 mM DTT, pH 7.6), supplemented with Roche EDTA-free Protease Inhibitor Cocktail and lysed by sonication. The insoluble pellet was collected for inclusion body extraction, resuspended, and washed twice with lysis buffer containing 0.1% v/v of Triton-X and once with lysis buffer without detergent. The pellet was then solubilized in 6 M guanidine hydrochloride, 20 mM Tris-HCl, 1 mM EDTA, 100 mM NaCl (pH 7.5 at 4 °C) for 2 hr at 4 °C. The mixture was then centrifuged, and the supernatant was collected and dialyzed against buffer containing 7 M urea, 10 mM Tris-HCl, 1 mM EDTA, 100 mM NaCl, 1 mM DTT, pH 7.5 at 4 °C, loaded onto a HiTrap SP HP cation exchange column (5 mL) (Cytiva), and eluted using a linear gradient of 0.1 to 1 M NaCl. Pure fractions were collected and loaded onto a Waters Xbridge BEH C18 prep-size column and purified by RP-HPLC. Fractions were collected using a gradient of 30–70% acetonitrile in water with 0.1% TFA. The isolated protein was characterized by SDS-PAGE, UV-absorbance, RP-HPLC, and MS. Protein concentrations were obtained by using extinction coefficients ε_M_ =  4470, 7450, 4470, and 5960 M^−1^cm^−1^ at 280 nm for H2A, H2B, H3, and H4, respectively. All histones were lyophilized and stored at − 80 °C prior to assembly into octamers.

### Histone 3 Lys 9 Trimethylation (H3K9me3)

Trimethylated Histone 3 Lys 9 (H3K9me3) was prepared using an analog as previously described^69^. A construct of H3 K9C (lysine to cysteine mutation) was expressed and purified similarly to the other histone proteins, as written above. The protein was then reduced in alkylation buffer (1 M HEPES, 4 M guanidium-HCl, 10 mM D/L-methionine, 20 mM DTT, pH 7.8) for 1 hr at 37 °C. The sample was then incubated with 400 mM (2-bromoethyl)-trimethylammonium bromide (Sigma) in the dark for 2.5 hr with mixing, fresh DTT was added, and then the reaction was further allowed to proceed in the dark for an additional 2.5 hr. Reaction completion was verified by RP-HPLC, and the reaction was quenched with 700 mM 2-mercaptoethanol. The product was loaded onto a Waters Xbridge BEH C18 prep-size column and purified by RP-HPLC using a 30–70% acetonitrile/water gradient with 0.1% TFA. Fractions were collected and confirmed by MS.

### DNA preparation

12mer nucleosome array DNA is a 2.1 kbp construct composed of 12 repeats of the 601 Widom sequence flanked by 30 bp linkers, used to position 12 histone octamers and form the 12mer nucleosome array complex^92^. The DNA was prepared by transforming DH5α cells with a plasmid containing the 12mer DNA sequence, and the cells were cultured in Terrific Broth for 18 hr at 37 °C^46^. The cells were harvested, resuspended in buffer containing 25 mM Tris-HCl, 50 mM glucose, 10 mM EDTA, pH 8.0, and lysed with the addition of a buffer containing 0.2 M NaOH and 1% sodium dodecyl sulfate. The lysate was then neutralized with 4 M potassium acetate and 2 N acetic acid. Plasmid DNA was precipitated using isopropanol precipitation, the pellet was then suspended in TE 10/50 buffer (10 mM Tris-HCl, 50 mM EDTA, pH 8.0) and incubated with 20 mg/mL RNase A at 37 °C for 12 hr. Proteins were extracted from the mixture using phenol-chloroform, and RNA was removed by polyethylene glycol (PEG) precipitation. The 12mer DNA sequence was excised from the plasmid using Eco-RV digestion (New England BioLabs), and the excess plasmid was removed via PEG purification. The purified 12mer DNA was concentrated by ethanol precipitation and stored in TE 10/0.1 buffer (10 mM Tris-HCl, 0.1 mM EDTA, pH 8.0), and frozen at − 20 °C. The same protocol was used to prepare the 155 bp nucleosome-binding MMTV buffer DNA, which is used to sequester excess octamer during 12mer array assembly. DNA was analyzed on a 5% TBE gel, stained by SYBR Gold (Life Technologies), and imaged using a Typhoon FLA 9500 (Cytiva) imager.

### Assembly of histone octamers and 12mer nucleosome arrays (12xNA)

12mer nucleosome arrays (12xNA) were assembled according to the following protocol^46,91^. Lyophilized histones were dissolved at 2 mg/mL in a buffer containing 6 M guanidine hydrochloride, 20 mM Tris-HCl, 5 mM dithiothreitol, pH 7.6 at 4 °C at a histone ratio of 1.0:1.0:0.9:0.9 of H2A:H2B:H3:H4. The octamers were refolded through dialysis into a buffer containing 2 M NaCl, 10 mM Tris, 1 mM EDTA, 1 mM dithiothreitol, pH 7.6. The octamers were then purified using a Superdex 200 10/30 column (Cytiva) to remove histone tetramers and dimers. The pure octamers were concentrated and stored in 50% glycerol at -20 °C. 12xNA were reconstituted by combining 1 equivalent 12mer DNA (601 sites), 0.3 equivalent MMTV DNA, and ~ 1.6 equivalent octamers in a buffer containing 10 mM Tris-HCl, 0.1 mM EDTA, 2 M KCl at pH 7.5 at 4 °C^91,93^. The combined DNA and octamers were then subjected to a salt gradient dialysis into a buffer containing 10 mM Tris-HCl, pH 7.5, 0.1 mM EDTA, 10 mM KCl for ~ 24 hr at 4 °C. The 12xNA were precipitated by the addition of ~2 mM MgCl_2_ to purify from MMTV mononucleosomes and excess MMTV DNA, and dialyzed into a buffer containing 20 mM Tris-HCl, pH 7.8 at 4 °C, 10 mM NaCl, 0.5 mM TCEP. The purity of 12 x NA was confirmed using APAGE gels (2% acrylamide, 1% Biorad agarose) stained with SyBr Gold (Life Technologies). Concentrations were determined using the molar extinction coefficient ε_A260_ = 2822957 M^−1^cm^−1^.

### HP1α expression and purification

BL21(DE3)-Rosetta *Escherichia coli* cells were co-transformed with the His-tagged HP1α and Casein kinase 2 (CK2) plasmids to make in-cell phosphorylated HP1α^94^. Briefly, cells were grown in Luria Bertani media with ampicillin (and streptomycin for pHP1α) at 37 °C and induced at an OD_600_ of 0.6 using 0.5 mM IPTG. Cells were transferred to 18 °C and pelleted by centrifugation after 16–24 hr. The pellet was resuspended in lysis buffer (1x PBS, pH 7.4, 300 mM NaCl, 10% glycerol, 7.5 mM imidazole) supplemented with Roche protease inhibitor tablets. The solution was sonicated, centrifuged, and the lysate supernatant was then incubated with Ni-NTA resin (5 mL resin per 1 L culture) with rotation at 4 °C for 1 hr. The lysate-resin was loaded onto a Bio-Rad Econo-Column and washed with wash buffer (1x PBS, pH 7.4, 300 mM NaCl, 10% glycerol, 7.5 mM imidazole), and then eluted with wash buffer containing 400 mM imidazole. The eluate was incubated with TEV-protease and dialyzed into imidazole-free buffer overnight at 4 °C. The solution was then adjusted to 6 M guanidine-HCl and purified using a Waters XBridge BEH C18 prep-size RP-HPLC column using a gradient of 10-60% acetonitrile in water with 0.1% TFA. Pure fractions were combined, lyophilized, and stored at − 80 °C. Protein concentration was obtained by using an extinction coefficient ε_M_ =  29160 M^−1^cm^−1^ at 280 nm. Mass spectrometry analysis has indicated that this protocol results in the predominant phosphorylation of S11, S12, S13, and S14 on the HP1α N-terminus, with relatively minimal phosphorylation elsewhere in the sequence^51^.

To refold, lyophilized HP1α was resuspended into resuspension buffer (20 mM HEPES, pH 7.2, 6 M guanidine-HCl, 20 mM NaCl, 2 mM DTT) at 1 mg/mL, and refolded by dialyzing into 20 mM HEPES, 150 mM NaCl, 1 M guanidine-HCl at pH 7.2 for 2-3 hr, and then dialyzing against guanidinium-free buffer with 300 mM NaCl overnight at 4 °C. 300 mM NaCl is used to prevent phase separation of pHP1α during the concentration step. Unphosphorylated HP1α was expressed and purified by the same procedure, except that the CK2 plasmid was omitted in the initial transformation step and streptomycin was excluded from the media.

### Fluorophore conjugation

Ki-67* was labeled on its native cysteines using Cy5 maleimide (Lumiprobe). To label, 1.5X molar excess Cy5-maleimide was added to a final concentration of 30 μM Ki-67* at pH 6.7 overnight at 25 °C with rotation and was then quenched with excess β-mercaptoethanol. Labeled Ki-67* was purified using a PD-10 desalting column (Cytiva). Fluorophore conjugation was confirmed by ESI-TOF-MS, SDS-PAGE, and fluorescence was visualized using a Typhoon FLA 9500 (Cytiva) imager.

Meanwhile, HP1α was labeled with Cy3 on a non-native cysteine. For this purpose, we used a construct containing a GSKCK tag added to its C-terminus to avoid perturbing the native cysteines in the CD and CSD domains^27,94^. This construct was expressed and purified by the same protocol as HP1α (see above). To label, excess Cy3-maleimide was added to 100 μM HP1α, and then quenched with excess β-mercaptoethanol after a 10 s reaction, favoring labeling of only the most solvent-exposed cysteine. The labeled protein was purified by RP-HPLC from excess Cy3 using a 10–60% acetonitrile in water with 0.1% TFA. Pure fractions were lyophilized and refolded similarly to HP1α and pHP1α.

### In vitro phosphorylation of Ki-67*

Ki-67* was phosphorylated in vitro similarly to a previous protocol^40^. 50 µL of 75 µM Ki-67* was incubated with ~ 0.7 μg CDK1–cyclin B1 (Abcam) in 1X Protein kinase buffer (NEB), 0.5 mM TCEP, 2.5 mM adenosine-5′-triphosphate (ATP; Gold Bio) at 30 °C for 16 hr. The kinase was then heat-inactivated for 20 min at 65 °C, followed by centrifugation to remove precipitated kinase. Phosphorylation was verified via SDS-PAGE and MS.

### Aurora B Kinase expression and purification

BL21(DE3)-Rosetta *Escherichia coli* cells were transformed with the His and Glutathione S-transferase (GST) tagged Aurora B Kinase construct with a thrombin cleavage site (pHH0103_AURKB_Pkinase was a gift from Sachdev Sidhu (Addgene plasmid # 110025; http://n2t.net/addgene:110025; RRID:Addgene_110025)^95^. Aurora B Kinase was expressed and purified as previously described^27,65^. Cells were grown in Luria Bertani media with ampicillin at 37 °C, induced at an OD_600_ of 0.6-0.8 using 0.5 mM isopropyl-β-D-thiogalactoside (IPTG), transferred to 18 °C for 16–24 hr, and pelleted by centrifugation. The bacterial pellet was resuspended in lysis buffer (50 mM Tris-HCl, 500 mM KCl, 0.5 mM TCEP, 10 mM imidazole, pH 7.8 at 4 °C, supplemented with Roche EDTA-free protease inhibitor cocktail tablets. Cells were lysed by sonication, and the soluble fraction was collected by centrifugation. The supernatant was then incubated with Ni-NTA resin (5 mL resin per 1 L culture) with rotation at 4 °C for 1 hr, and the lysate-resin was loaded onto a Bio-Rad Econo-Column and washed with wash buffer (50 mM Tris-HCl, 500 mM KCl, 0.5 mM TCEP, 10 mM imidazole, pH 7.8 at 4 °C), and then eluted with wash buffer containing 400 mM imidazole. The eluate was dialyzed into 20 mM HEPES, 50 mM KCl, 0.5 mM TCEP, pH 7.2 buffer overnight at 4 °C. Then, the sample was loaded onto a HiTrap SP HP cation exchange column (5 mL) (Cytiva), and eluted using a linear gradient from 50 mM to 1 M KCl over 1 hr. Pure fractions were then dialyzed into 20 mM Tris-HCl, 50 mM KCl, pH 7.8 at 4 °C, 0.5 mM TCEP, concentrated to ~ 20 μM or higher, supplemented with 25% glycerol, aliquoted, flash frozen, and stored at − 80 °C until use. The isolated protein was characterized by SDS-PAGE, HPLC and MS. Protein concentration was obtained by using an extinction coefficient of 84230 M^−1^cm^−1^ at 280 nm. Kinase activity was verified by immunoblotting for H3S10 phosphorylation (Cell Signaling Technologies, #9701).

### In vitro phosphorylation of 12mer nucleosome arrays (12xNA)

The phosphorylation protocol was modified from previous protocols^27,65^ with additional purification steps. Freshly made unmodified or H3K9me3-modified 12xNA were prepared as described above and dialyzed into 20 mM HEPES, 10 mM KCl, 0.5 mM TCEP, pH 7.2. 1 μM of 12xNA (601 sites) was incubated with 4 μM Aurora B kinase, 0.2 mM adenosine-5′-triphosphate (ATP; Gold Bio), 1 mM MgCl_2_, 50 mM KCl, and 1 mM PMSF. 50 mM KCl was critical to avoid the precipitation of Aurora B Kinase at low salt, and PMSF is crucial for avoiding degradation of the histone tails. The reaction was then incubated at 30 °C for 5 hr. The 12xNA were then precipitated by the addition of ~ 5 mM MgCl_2_, in order to pellet and purify 12xNA from Aurora B kinase and most reactants. The phosphorylated 12xNA were resuspended and dialyzed into a buffer containing 20 mM Tris-HCl, pH 7.8 at 4 °C, 10 mM KCl, 0.5 mM TCEP. The purity of 12xNA was confirmed using APAGE gels (2% acrylamide, 1% Biorad agarose) stained with SyBr Gold (Life Technologies). Concentrations were determined using molar extinction coefficient ε_A260_ = 2822957 M^−1^cm^−1^ at 260 nm. Phosphorylation was confirmed by immunoblotting. Each replicate using H3S10 phosphorylated nucleosome arrays was an independent phosphorylation reaction.

### Immunoblotting

Purified phosphorylated 12xNA (100 ng) were run on a 18% SDS-PAGE gel and transferred onto polyvinylidene difluoride (PVDF) membranes via tank immersion. Blocking and antibody incubation steps were performed in Tris-buffered saline with 0.1% Tween-20 (TBST) and 5% BSA. Following blocking, the blot was incubated with a Phospho-Histone H3 (Ser10) primary antibody (Cell Signaling Technologies #9701) at a 1 to 1000 dilution overnight at 4 °C. After wash steps, the blot was incubated with an Anti-rabbit IgG, HRP-linked Antibody (Cell Signaling Technologies #7074) at a 1 to 1000 dilution for two hours at 25 °C. Following wash steps, the blot was incubated in chemiluminescent substrate (Thermo Scientific Pierce™ ECL Western Blotting Substrate) and imaged using a BioRad ChemiDoc. Exposure time was automatically selected to avoid pixel saturation.

### Confocal imaging of droplets

For droplet imaging, images were acquired using a Leica SP8 microscope with a 60X oil immersion objective. Phase separation was induced immediately before imaging. Samples (5 µL) were placed in a µ-Slide 18-well glass bottom multi-well plate (Ibidi) and were allowed to settle for 5–10 min prior to imaging. Leica LASX (v.3.5.7.23225) and Fiji/ImageJ (v.2.9.0) were used for image processing and analysis.

### Fluorescence recovery after photobleaching (FRAP)

Condensates were prepared as described above and imaged using a Leica SP8 confocal microscope. In each experiment, 3 to 4 μm regions of interest were bleached at 100% laser power in the center of each condensate. Two pre-bleach frames were acquired, and 60 post-bleaching frames were acquired (1.295 s/frame), corresponding to 1 min of recovery time. Data were analyzed using Fiji/ImageJ. The FRAP curves were normalized to the maximal prebleach and minimal postbleach intensities, and an unbleached reference was used to correct for photobleaching due to image acquisition. The error bars represent standard deviation from data collected with 10–35 condensates from at least two independent samples, and the curve represents the best fit to a one-phase decay model.

### Turbidity assays

Turbidity of protein samples was obtained from the optical density at 600 nm using a NanoDrop spectrophotometer (ThermoFisher). Protein solutions were pre-incubated at 25 °C, and proteins were thoroughly mixed at a 10 μL final volume and immediately measured for turbidity. All turbidity measurements were conducted in 20 mM Tris-HCl, 75 mM KCl, 0.5 mM TCEP, pH 7.2 at 25 °C. Two technical replicates were obtained for each sample, as well as three to four independent replicates per condition.

### Native PAGE binding assays

Native polyacrylamide gel electrophoresis (PAGE) binding assays were performed using either HP1α CSD-CTE or HP1α CSD-CTE W174A with several different Ki-67* constructs, namely Ki-67*, Ki-67* with residues 340-344 (PVVIM) deleted, and Ki-67* with residues 340-344 (PVVIM) and 475-479 (AKVLM) mutated to alanines. The samples were prepared in 20 mM HEPES, pH 7.2, 300 mM KCl, 1 mM TCEP, and 10% glycerol. The concentration of the CSD-CTE constructs was 25 μM (i.e., 12.5 μM dimer), while the concentration of Ki-67* constructs ranged from 0.01 μM to 125 μM. The samples were incubated at room temperature for 20 min after mixing, and 10 μL of each sample was loaded onto a 5% TBE native gel. The gel running condition was 180 V for 30 min at 4 °C in 1X TBE running buffer. The gels were stained with Coomassie and images were acquired using a Biorad Chemidoc MP imager. The images were analyzed using ImageJ and data was plotted using GraphPad Prism. The binding data was fit using equation 5 in ref. ^96^.

### NMR Experiments

The NMR samples were prepared in 20 mM HEPES, pH 7.2, 75 mM KCl, and 1 mM TCEP with 10% D_2_O. Samples contained ^15^N labeled 50 μM HP1α CSD-CTE with either 2.5 μM, 5 μM, or 25 μM Ki-67*. Data was collected on an 800 MHz Neo Bruker NMR spectrometer. Pulses were calibrated using the standard TopSpin protocol. HSQC experiments were performed with the pulse program fhsqcf3gpph^97^, with 32 scans and a relaxation delay of 1.5 s. The following parameters were set for the ^1^H dimension: 13.583 ppm spectral width, frequency offset of 4.717 ppm, 2048 points and DQD acquisition mode. The following parameters were used for the ^15^N dimension: 36.00 ppm spectral width, frequency offset of 120 ppm, 128 points and States-TPPI acquisition mode. Data was processed in Bruker TopSpin and analyzed in POKY^98^, while GraphPad Prism was used to prepare the intensity plots. The intensity cutoff for quantifying cross-peaks was $4\,\times \,{\sigma }_{{noise}}$ where ${\sigma }_{{noise}}$ was the average noise level of the spectrum as calculated by POKY. The plotted intensity ratios represent ${I}_{{bound}}/{I}_{{free}}$ where ${I}_{{free}}$ represents the intensity of cross-peaks in the HP1α CSD-CTE spectrum, while ${I}_{{bound}}$ represents the intensity of the matching cross-peaks in the spectrum with Ki-67*. Error bars were calculated using the equation $\sqrt{{\left({\sigma }_{{bound}}/{I}_{{bound}}\right)}^{2}+{\left({\sigma }_{{free}}/{I}_{{free}}\right)}^{2}}$ .

### Coarse-grained molecular dynamics simulations

Coarse-grained (CG) coexistence simulations were performed using the HOOMD-Blue 4.7 software package^99^ with slab geometry as described previously^100–102^. Proteins were modeled using the one-bead-per-residue HPS-Urry model^63^, with parameters for phosphorylated serine and threonine taken from the HPS-PTM model^103^. For HP1α and pHP1α homodimers, the folded domains were treated as rigid bodies^104^ to prevent unfolding while allowing them to translate and rotate freely. Each CD was constrained as an independent rigid body, while the two CSD monomers were constrained together as a single rigid body to maintain the dimerization interface. The disordered regions (NTE, hinge, and CTE) remained fully flexible, enabling interactions between all protein segments throughout the simulation. In simulations with HP1α (or pHP1α) at molar ratios of 1:1, Ki-67^*^ was treated as a flexible chain to reflect its intrinsically disordered nature. Mixtures of the two components (40 chains each) were initialized in a simulation box of dimensions 210 × 210 × 1470 Å^3^. The box size and the number of protein chains were chosen to minimize finite-size effects, consistent with our previous work^105^. Coexistence slab simulations were conducted for 5 μs in the canonical (NVT) ensemble at 320 K using a Langevin thermostat with friction coefficient γ = m_AA_/τ, where m_AA_ is the mass of each amino acid bead, and τ is the damping time constant set to 1000 ps. The elevated temperature of 320 K was used based on our previous analysis^51^, which showed that this temperature ensures sufficient protein density in the dilute phase for accurate characterization of the coexistence behavior. The integration time step was 10 fs. The first 1 μs of each trajectory was discarded as equilibration before calculating density profiles and contact maps. Protein density was analyzed as a function of the z-coordinate to distinguish the dense and dilute phases. For contact analysis, two residues were considered in contact if their inter-bead distance was less than 1.5 times the arithmetic mean of their van der Waals diameters. Simulation snapshots were visualized using VMD^106^.

### Reporting summary

Further information on research design is available in the Nature Portfolio Reporting Summary linked to this article.

## Supplementary information

Supplementary Information

Description of Additional Supplementary Files

Supplementary Data 1

Reporting Summary

Transparent Peer Review file

## Source data

Source Data

## Data Availability

Source data are provided with this paper. CUT&RUN and RNA-seq data generated in this study have been deposited in the BioProject/SRA database under accession code PRJNA1242856 [https://www.ncbi.nlm.nih.gov/sra?term=SRP573657]. NMR assignments of the CSD-CTE homodimer of HP1α have been deposited in the BMRB under accession code 53698. Mass spectrometry data has been deposited in MassIVE archive under accession code MSV000102862. Source data are provided in this paper.
