# Supplementary material for: Ki-67 regulates heterochromatin organization in neurons and forms condensates with heterochromatic components
Source: Nat Commun. 2026 Sep 8;17:10615. doi: 10.1038/s41467-026-77430-2 (PMC13649782; doi:10.1038/s41467-026-77430-2)
Supplement: Supplementary file 1 [file 41467_2026_77430_MOESM1_ESM.pdf]

Ki-67 regulates heterochromatin organization in neurons and forms condensates with heterochromatic components

Lannah S. Abasi[1], Challana Tea[1,2], Rushabh Bhakta[1], Donald Le[2], Tien M. Phan[3], Jeetain Mittal[3,4,5], Cole J. Ferguson[2*], Galia T. Debelouchina[1*]

[1]*Department of Chemistry and Biochemistry, University of California San Diego, La Jolla, CA 92093, USA*

[2]*Department of Pathology, University of California San Diego, La Jolla, CA 92093, USA*

[3]*Artie McFerrin Department of Chemical Engineering, Texas A&M University, College Station, TX 77843, USA*

[4]*Department of Chemistry, Texas A&M University, College Station, TX 77843, USA*

[5]*Interdisciplinary Graduate Program in Genetics and Genomics, Texas A&M University, College Station, TX 77843, USA*

**Correspondence: cferguson@health.ucsd.edu and gdebelouchina@ucsd.edu*

SUPPLEMENTARY FIGURES

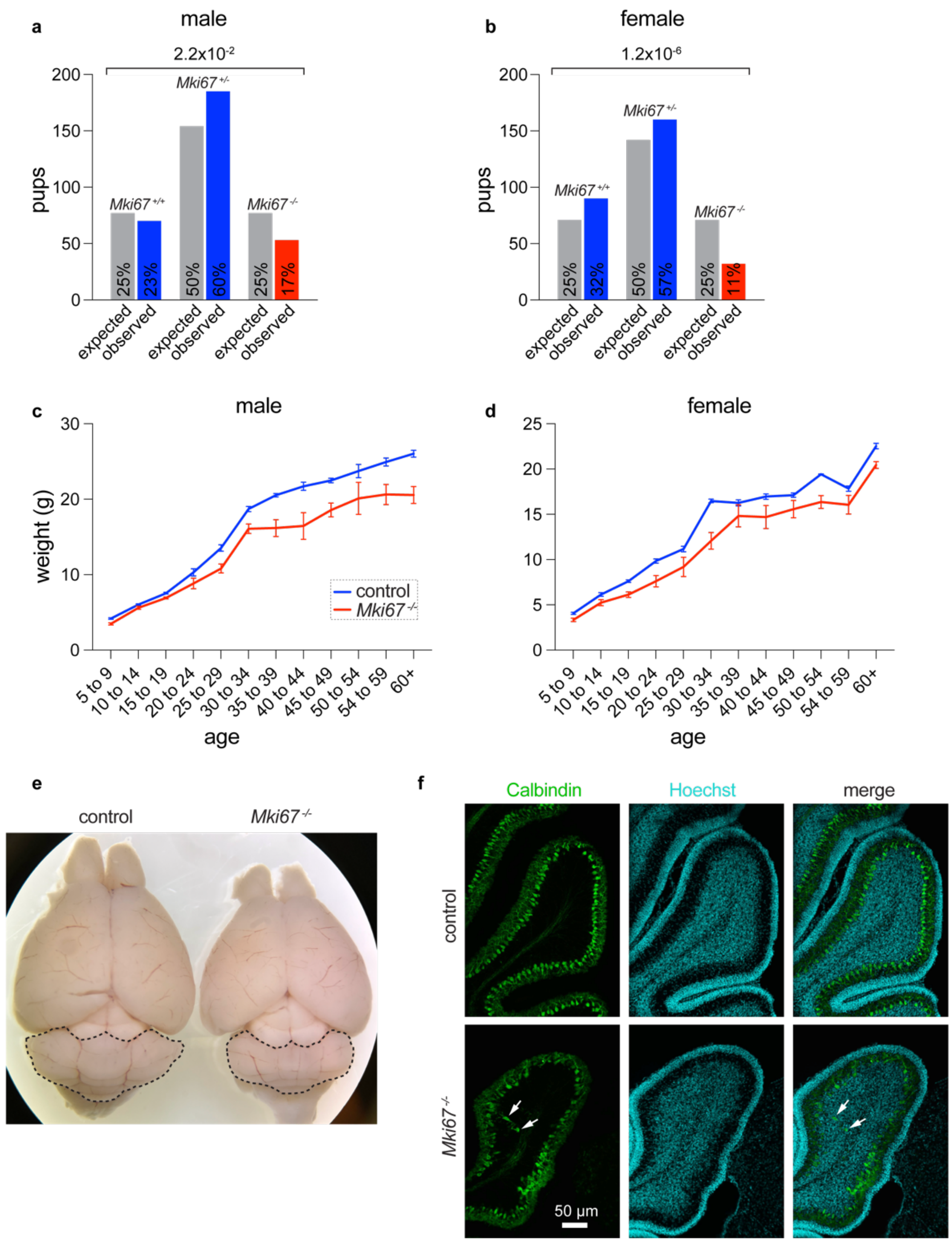

Supplementary Figure 1. Characterization of *Mki67* $^{-/-}$ mice. a) Genotype distribution of male pups born from crosses of heterozygous parents. Pups were genotyped between the ages of P4 and P7. P-value by chi-square test. Source data are provided as a source data file. b) Genotype distribution of female pups. P-value by chi-square test. Source data are provided as a source data file. c) Weights of control (*Mki67* $^{+/+}$ and *Mki67* $^{+/-}$) and mutant (*Mki67* $^{-/-}$) male mice from P5-

P60, binned into five-day intervals. Error bars represent standard error of the mean. Source data are provided as a source data file. d) Corresponding data for the weights of female mice from P5-P60, binned into five-day intervals. Error bars represent standard error of the mean. Source data are provided as a source data file. e) Representative images of control and *MKi67* $^{-/-}$ mouse brains at age P13, documenting cerebellar hypoplasia. f) Confocal microscopy images of P8 cerebella from control and *MKi67* $^{-/-}$ mice. Purkinje neurons were identified using anti-Calbindin immunofluorescence in frozen sections. Arrows represent ectopic Purkinje neurons.

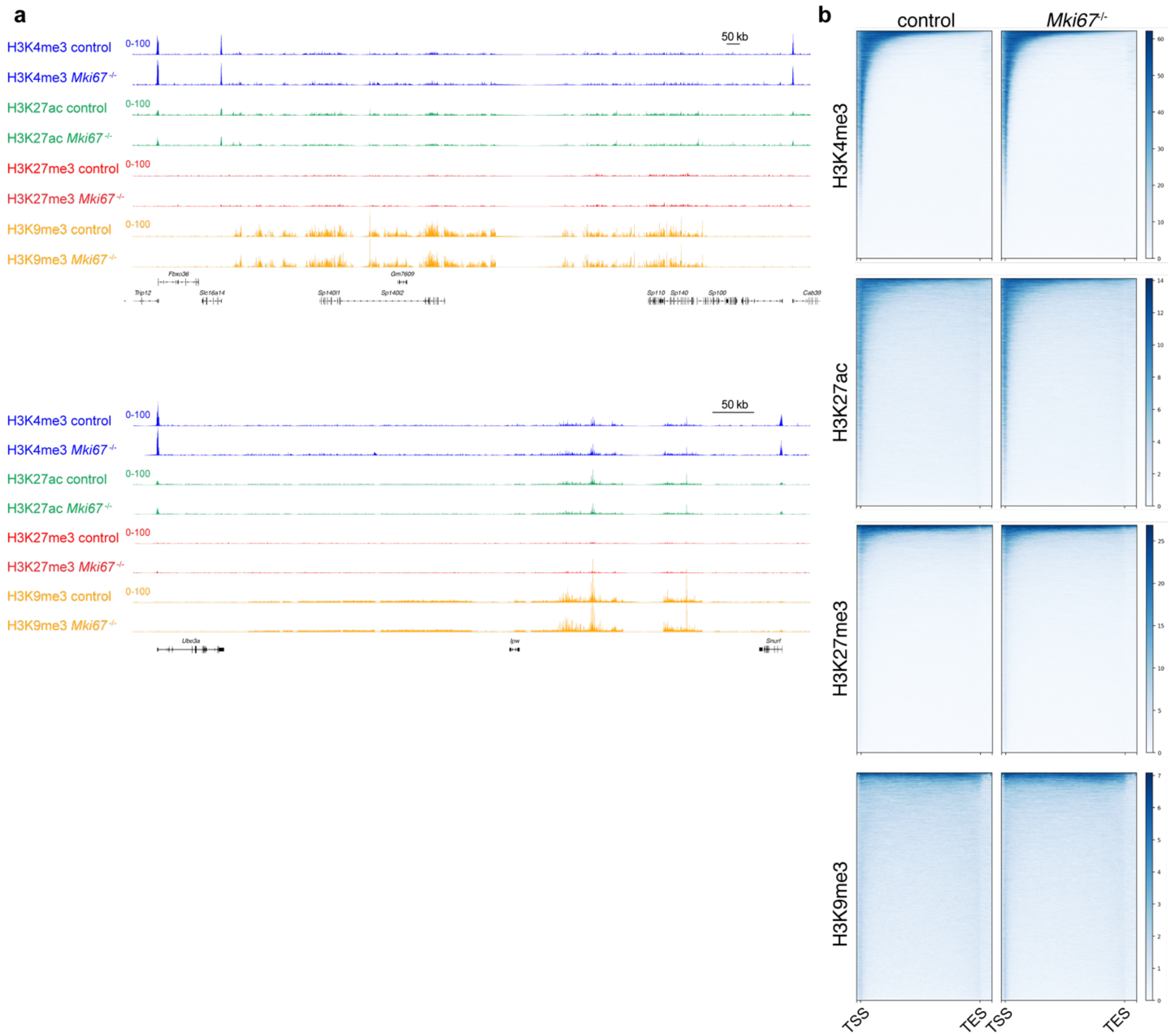

Supplementary Figure 2. Genomic analysis of key regulatory histone PTMs in the cerebellum of *Mki67* $^{-/-}$ mice. a) CUT&RUN coverage tracks of H3K4me3, H3K27ac, H3K27me3, and H3K9me3 from P13 cerebella of control and *Mki67* $^{-/-}$ mice. b) Metagene heatmaps of CUT&RUN detection of H3K4me3, H3K27ac, H3K27me3, and H3K9me3 across all 22597 protein-coding genes. The color scale reflects normalized coverage.

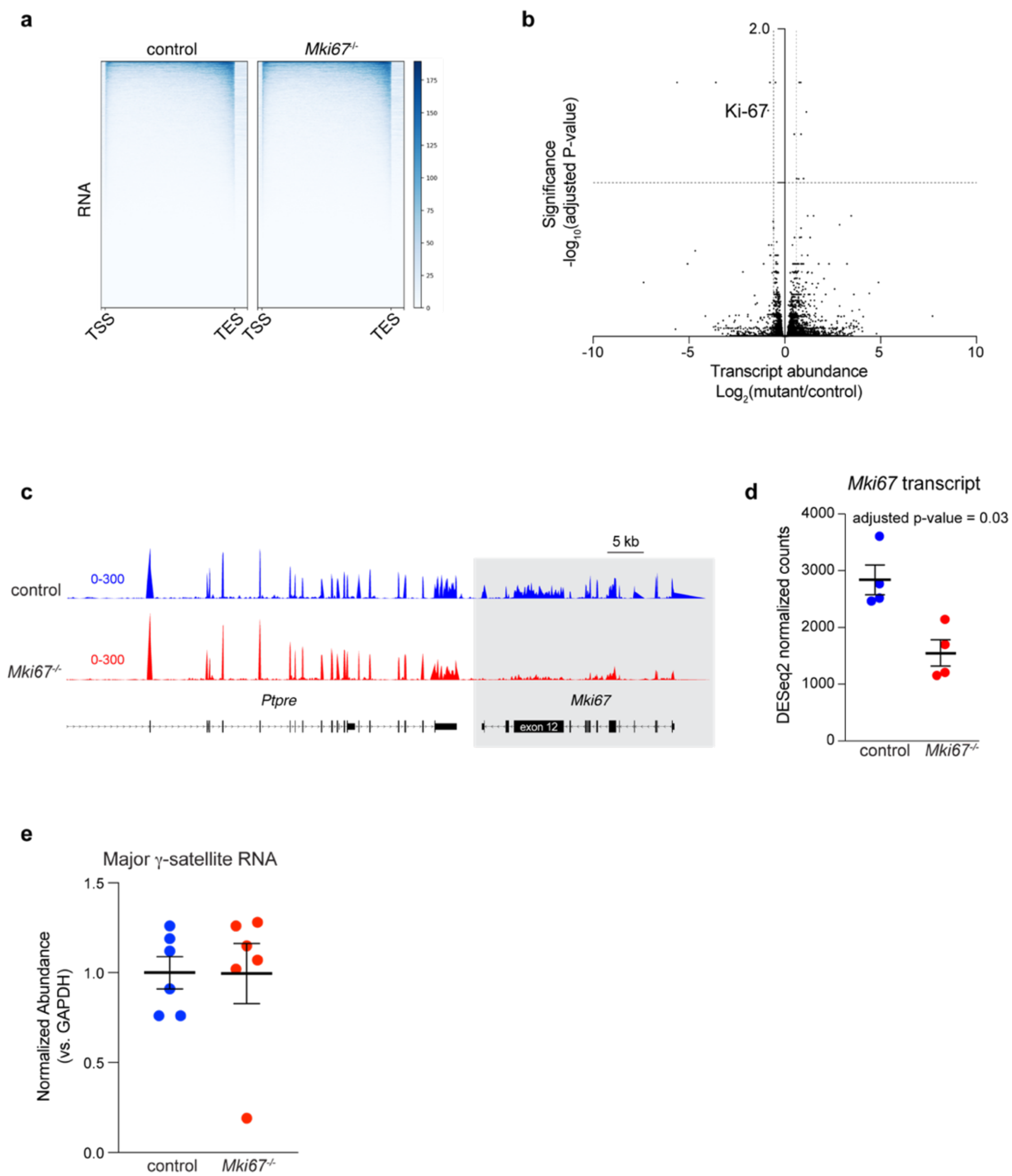

Supplementary Figure 3. RNAseq analysis of *Mki67*$^{-/-}$ mice. a) Metagene heatmap of RNAseq data across protein coding genes. The color scale reflects raw coverage. b) Volcano plot of differential expression between control and mutant *Mki67*$^{-/-}$ mice at P10-11 (n=4 mice). Source data are provided as a source data file. c) RNAseq coverage tracks at the *Mki67* locus. Exon 12 encodes the disordered Ki-67 repeat domains. d) DESeq2-normalized counts and adjusted p-values (n = 4 mice). Source data are provided as a source data file. e) Quantitative RT-PCR assessment of non-coding RNAs transcribed from the major satellite repeat (n = 6 mice). Error bars represent standard error of the mean. Source data are provided as a source data file.

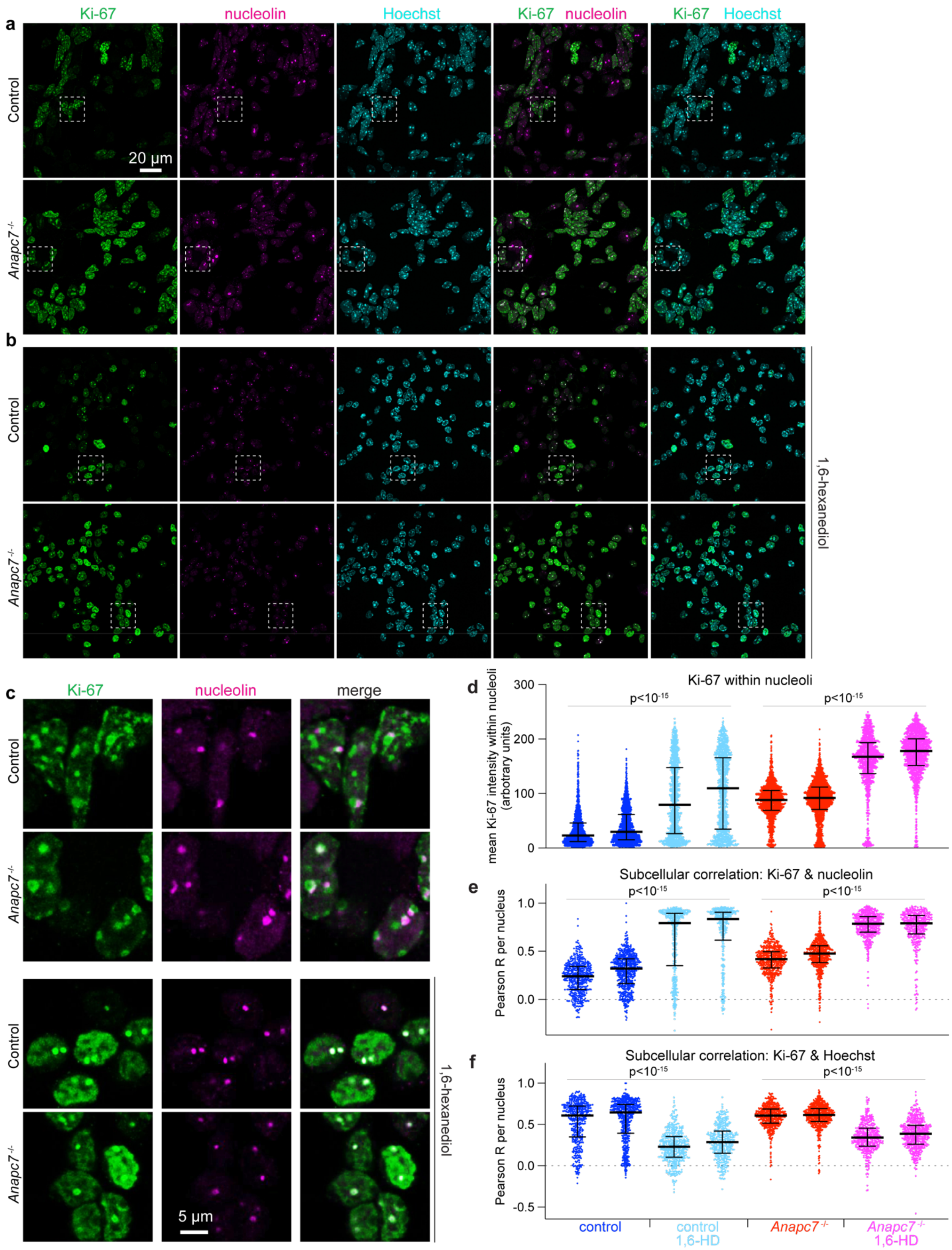

a
Ki-67
nucleolin
Hoechst
Ki-67 nucleolin
Ki-67 Hoechst
Control
Anapc7-/-
20 µm
b
Control
Anapc7-/-
1,6-hexanediol
c
Ki-67
nucleolin
merge
Control
Anapc7-/-
1,6-hexanediol
5 µm
d
Ki-67 within nucleoli
mean Ki-67 intensity within nucleoli (arbotrary units)
p<10-15
e
Subcellular correlation: Ki-67 & nucleolin
Pearson R per nucleus
f
Subcellular correlation: Ki-67 & Hoechst
control
control 1,6-HD
Anapc7-/-
Anapc7-/- 1,6-HD

Supplementary Figure 4. Redistribution of neuronal Ki-67 away from constitutive heterochromatin and into nucleoli upon 1,6-hexanediol treatment. a) Immunofluorescence (IF) detection of Ki-67 and nucleolin followed by confocal microscopy of cerebellar granule neuron cultures on day 2 *in vitro* (DIV2). b) Granule neuron cultures on DIV2 treated with 7% 1,6-hexanediol for 10 minutes before immunofluorescence. c) Higher magnification of boxed regions outlined in the above figures. d) Quantification of Ki-67 fluorescence intensity within nucleoli, as identified by nucleolin immunofluorescence. Each dot represents a cell. Two biological replicates are shown for each condition, but were combined for statistical analysis. Error bars depict the median and interquartile ranges. P-values by Kruskal-Wallis test followed by Dunn's test. Source data are provided as a source data file. e) Subcellular correlation (Pearson R value) between Ki-67 and nucleolin. Error bars depict the median and interquartile ranges. P-values by Kruskal-Wallis test followed by Dunn's test. Source data are provided as a source data file. f) Subcellular correlation of Ki-67 and Hoechst. Error bars depict the median and interquartile ranges. P-values by Kruskal-Wallis test followed by Dunn's test. Source data are provided as a source data file.

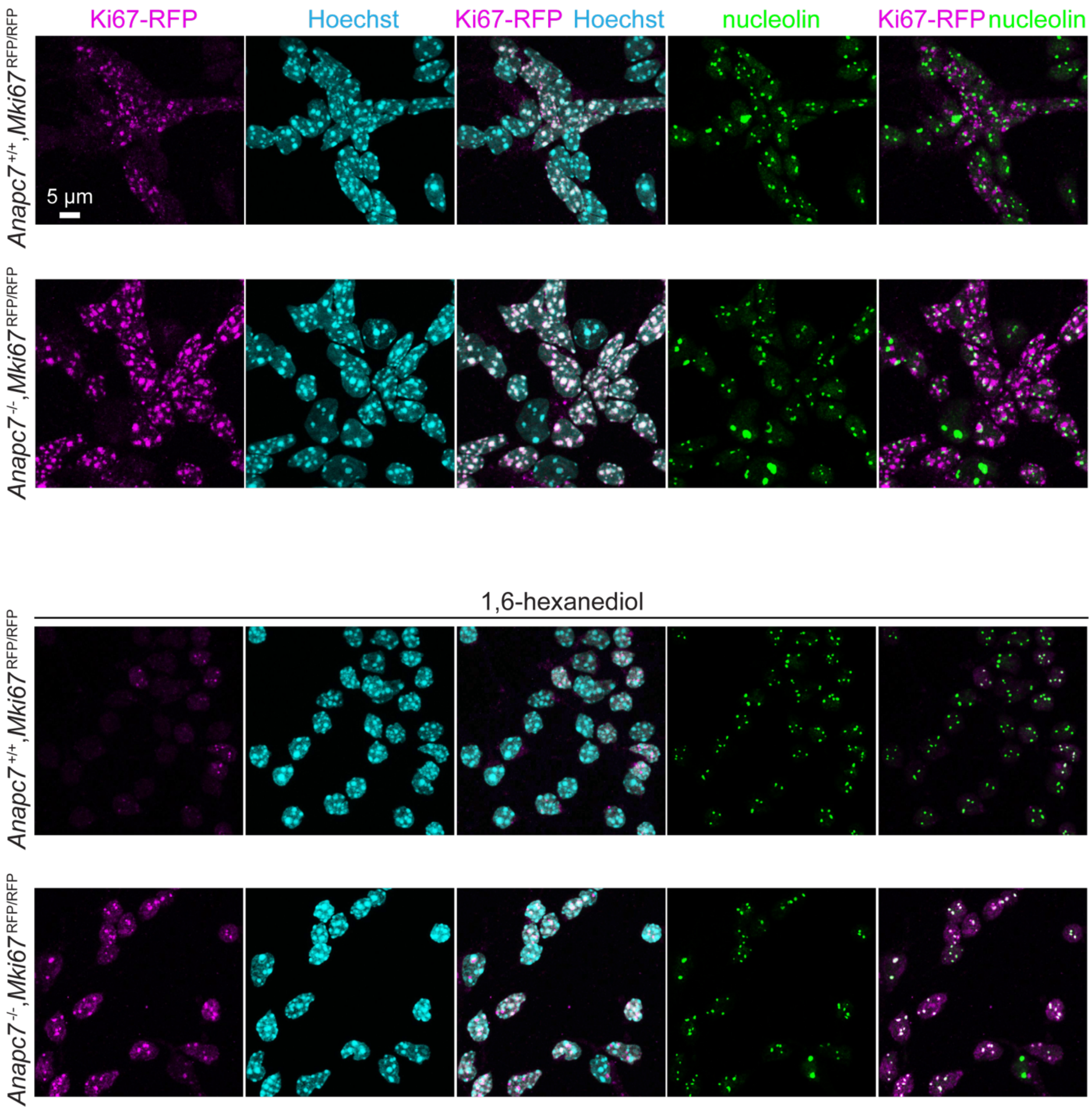

Supplementary Figure 5. 1,6-hexanediol treatment of cultured neurons from mice harboring a reporter for endogenous Ki-67 (*Mki67* ^{RFP}). a) Confocal microscopy images of untreated cerebellar granule neurons of the indicated genotypes on DIV2. Nucleolin was detected by immunofluorescence. b) Granule neuron cultures on DIV2 treated with 7% 1,6-hexanediol for 10 minutes before immunofluorescence.

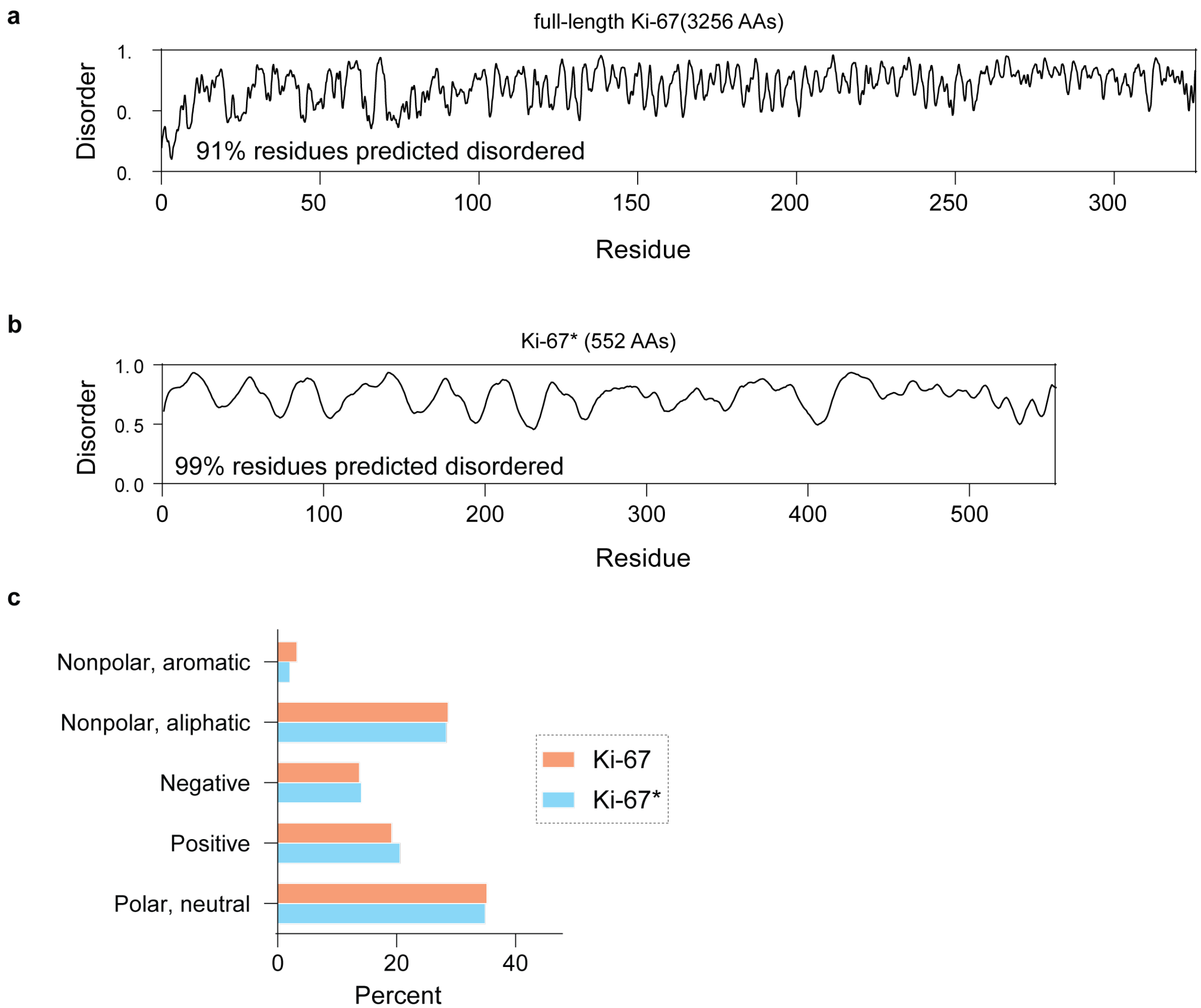

Supplementary Figure 6. Biochemical features of Ki-67*. a) IUPred3 output of full-length Ki-67. Regions with disorder values above 0.5 are predicted to be disordered. b) IUPred3 output of the Ki-67* construct. c) Comparison of amino acid composition of the full-length and Ki-67* constructs. Source data for all panels are provided as a source data file.

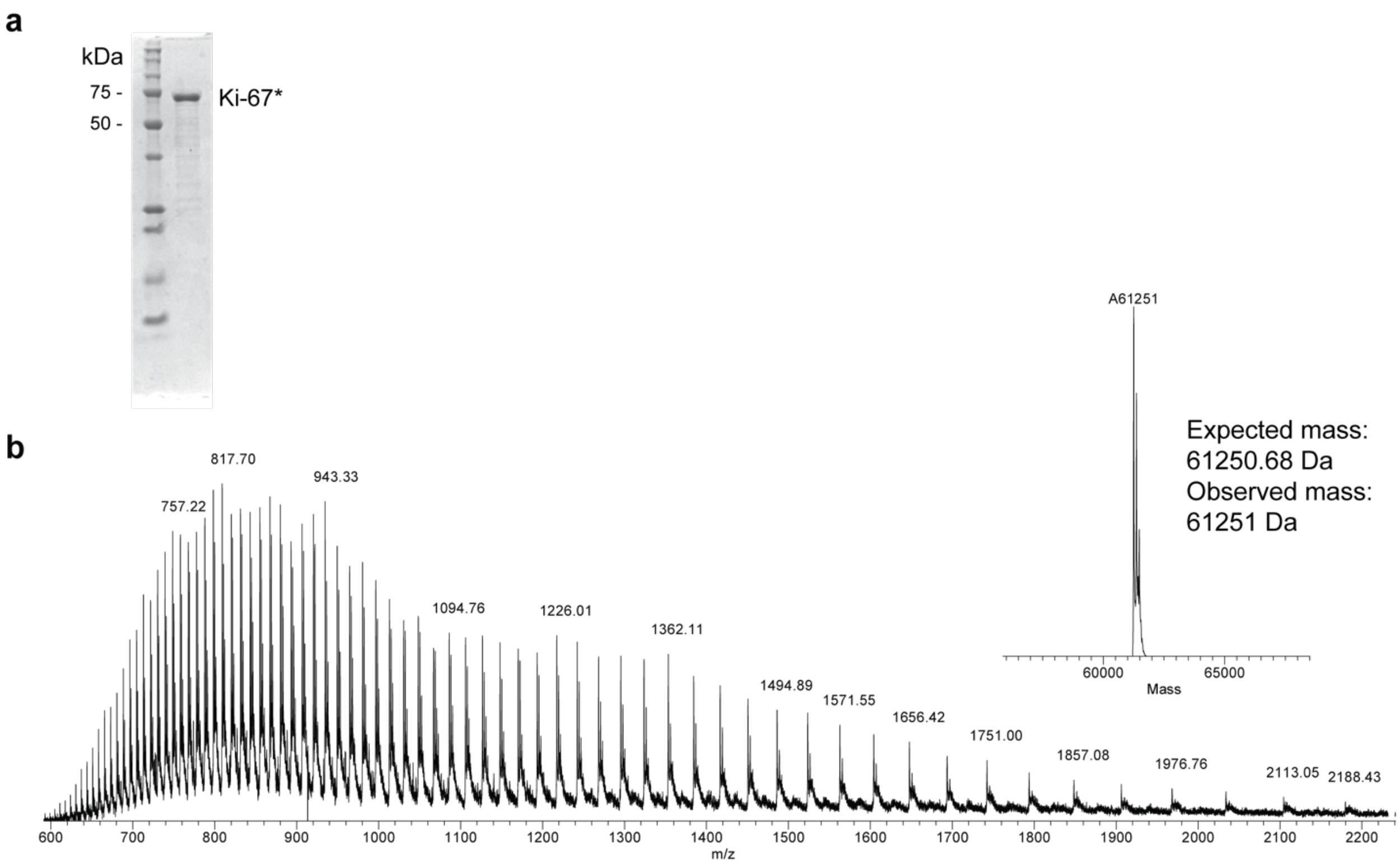

Supplementary Figure 7. Analysis of purified Ki-67*. a) Purified Ki-67* analyzed by SDS-PAGE and b) mass spectrometry. Analysis was performed on each new batch of purified protein.

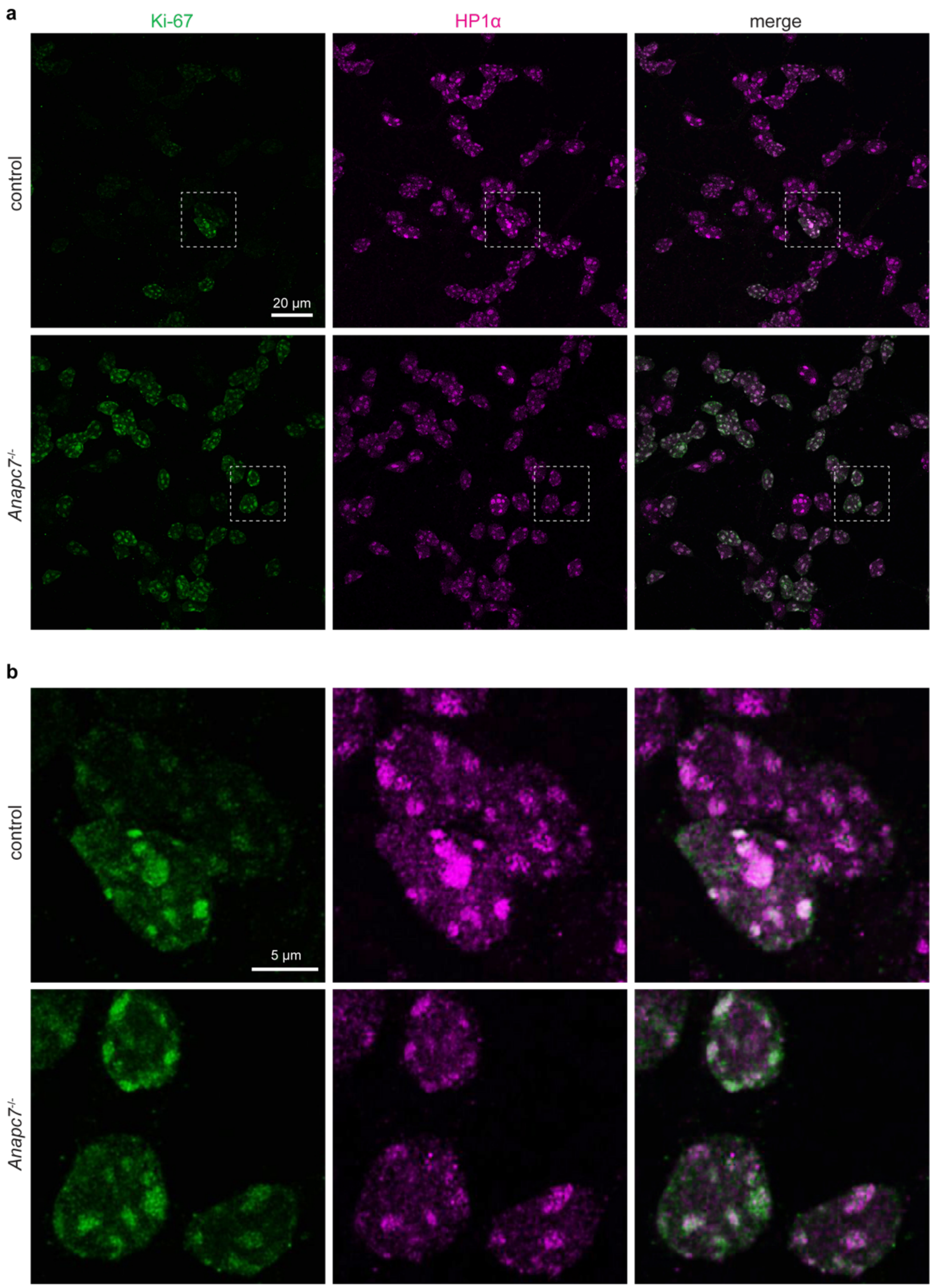

Supplementary Figure 8. Colocalization of endogenous HP1α and Ki-67 in neurons. a) Cultured cerebellar granule neurons of the indicated genotypes on DIV2 subjected to immunofluorescence labeling of Ki-67 and HP1α following fixation in 1:1 methanol:acetone. b) Higher magnification images of the boxed regions.

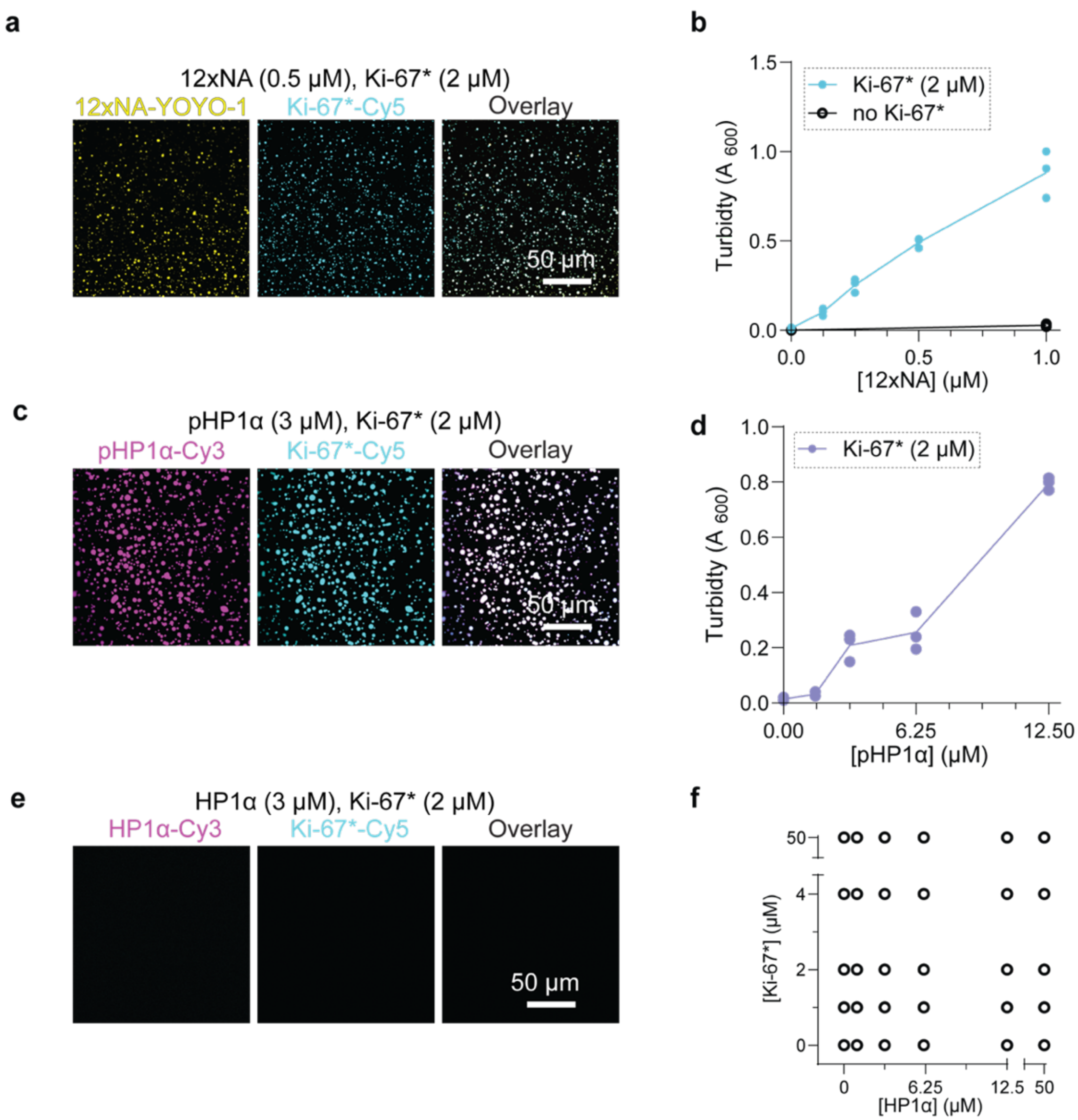

Supplementary Figure 9. Extended phase separation behavior data of Ki-67* and nuclear interactors. a) Confocal microscopy images of droplets of 2 µM Ki-67* with 40 nM 12mer nucleosome arrays (12xNA; 0.5 µM equivalent of mononucleosomes), visualized with 5% Ki-67*-Cy5 and 0.4 µM YOYO-1, respectively. b) Turbidity at A600 of increasing concentrations of 12xNA with or without 2 µM Ki-67*. Line connects the mean of 3 independent replicates. Source data are provided as a source data file. c) Confocal microscopy images showing colocalization of 3 µM pHP1α and 2 µM Ki-67* visualized with 5% pHP1α-Cy3 and 5% Ki-67*-Cy5, respectively. d) Turbidity at A600 of 2 µM Ki-67* with increasing concentrations of pHP1α. Line connects the

mean of 3 independent replicates. Source data are provided as a source data file. e) Confocal microscopy images of 3 μM HP1α and 2 μM Ki-67* visualized with 5% HP1α-Cy3 and 5% Ki-67*-Cy5, respectively. f) Phase diagram of Ki-67* liquid droplet formation at varying concentrations of HP1α. Open dots indicate no phase separation. Each condition was assessed twice by microscopy. Phase separation experiments were performed at least three times with independent batches of proteins with similar results.

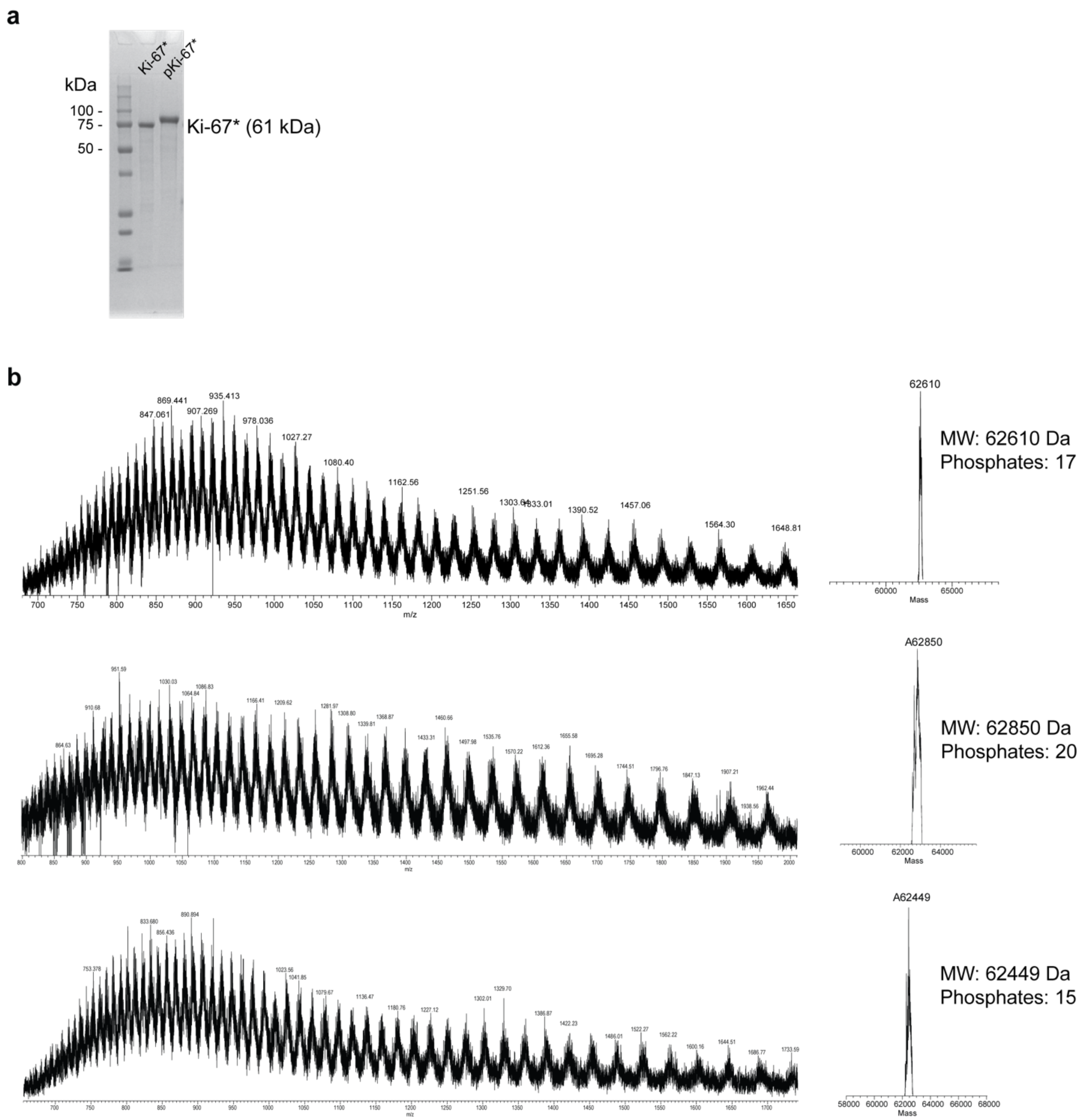

Supplementary Figure 10. Gel and mass spectrometry analysis of phosphorylated Ki-67*. a) Full image of the 12% SDS-PAGE gel of phosphorylated Ki-67* (pKi-67*) shown in **Fig. 5b**. b) MS analysis of individual replicates (R1, R2, R3 shown top to bottom) of the phosphorylation reactions shown in **Fig. 5c**. Number of phosphate groups is calculated by subtracting the mass of a phosphate group (80 Da).

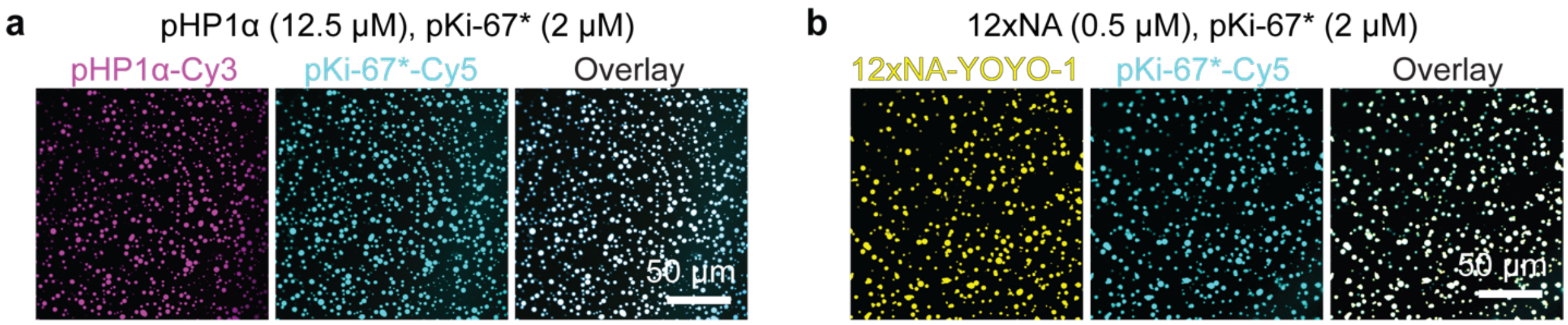

Supplementary Figure 11. Extended phase separation behavior data of phosphorylated Ki-67* and nuclear interactors. a) Confocal microscopy images of 3 μM pHP1α and 2 μM pKi-67* visualized with 5% pHP1α-Cy3 and 5% Ki-67*-Cy5, respectively. b) Confocal microscopy images of droplets formed with 2 μM pKi-67* and 40 nM 12mer nucleosome arrays (12xNA; 0.5 μM equivalent of mononucleosomes), visualized with 5% pKi-67*-Cy5 and 0.4 μM YOYO-1, respectively. Experiments were performed three times with independent batches of proteins with similar results.

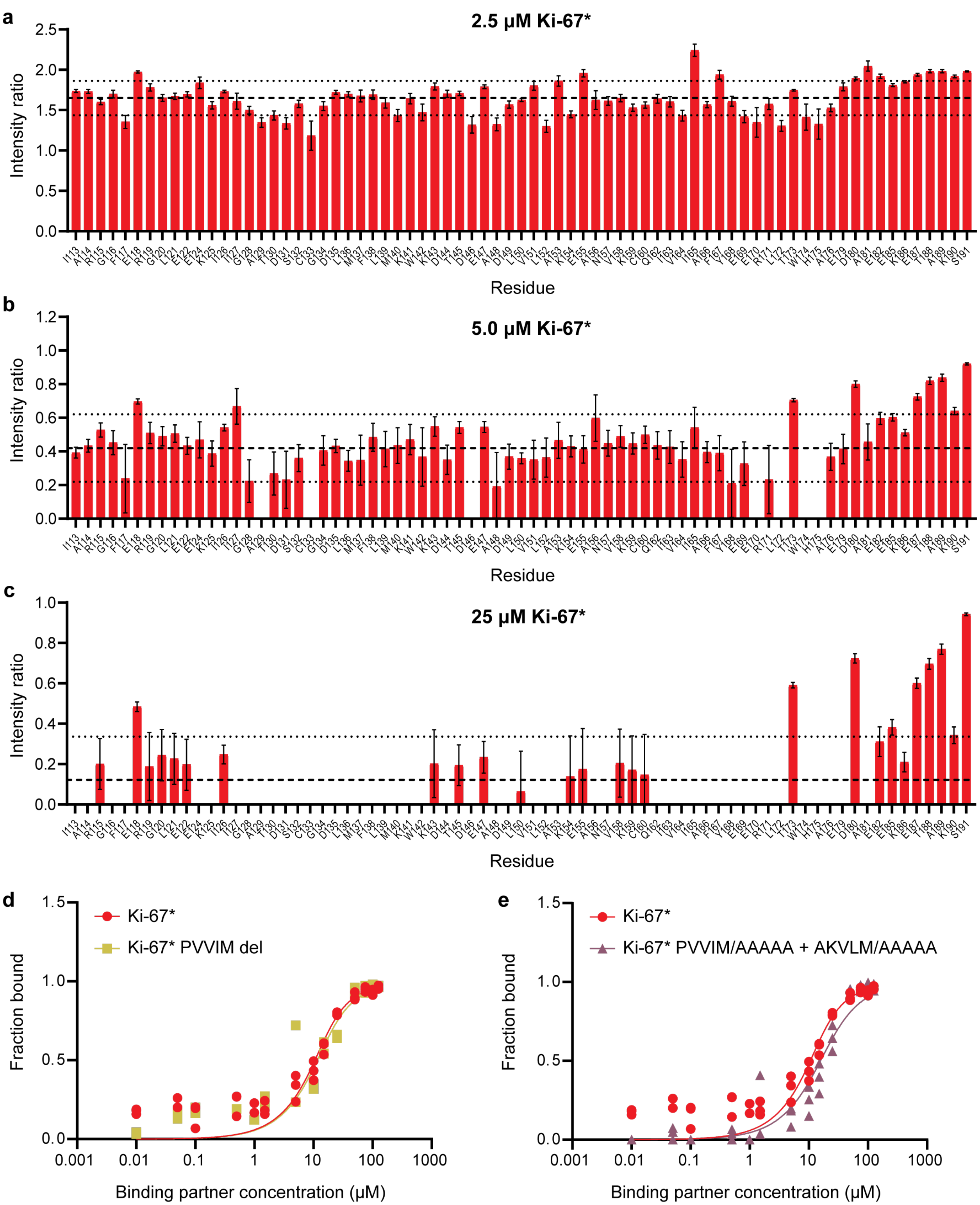
a
2.5 µM Ki-67*
Intensity ratio
Residue
b
5.0 µM Ki-67*
Intensity ratio
Residue
c
25 µM Ki-67*
Intensity ratio
Residue
d
Ki-67*
Ki-67* PVVIM del
Fraction bound
Binding partner concentration (µM)
e
Ki-67*
Ki-67* PVVIM/AAAAA + AKVLM/AAAAA
Fraction bound
Binding partner concentration (µM)

Supplementary Figure 12. **Analysis of the interactions between Ki-67* and HP1α CSD-CTE dimers.** a-c) Analysis of the intensity ratios of ^{1}H-^{15}N HSQC NMR experiments of 50 μM HP1α CSD-CTE with and without 2.5 μM, 5.0 μM, and 25 μM Ki-67*. Error bars are calculated based on the signal-to-noise ratio of the spectra (see Methods). The black dashed line indicates the average intensity ratio when all peaks are considered, while the dotted lines represent one standard deviation above and below. The 5.0 μM dataset was chosen for further analysis due to the balance of overall intensity ratio decrease and the detection of specific interaction sites. Titration was performed twice with similar results. d) Binding curves of 25 μM HP1α CSD-CTE with varying concentrations of Ki-67* (red) or Ki-67* with deletion of one of the putative HAC motifs, namely the PVVIM sequence (gold). The curves show data from three independent measurements. e) Binding curves of 25 μM HP1α CSD-CTE with varying concentrations of Ki-67* (red) or Ki-67* where two putative HAC motifs (the PVVIM and the AKVLM sequences) were mutated to alanine (purple). The curves show data from three independent measurements. Source data for all panels are provided as a source data file.

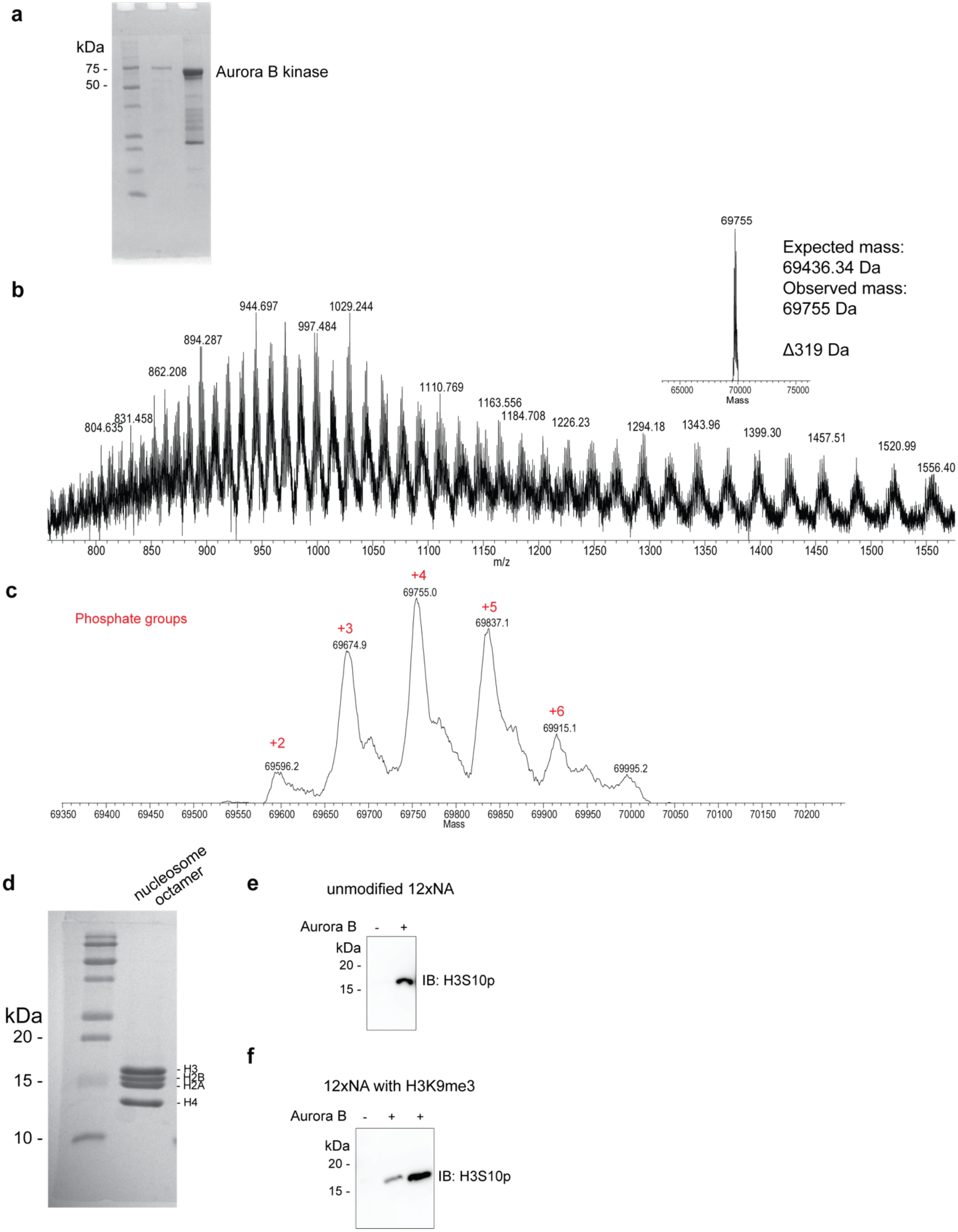
a
kDa
75 -
50 -
Aurora B kinase
b
69755
Expected mass:
69436.34 Da
Observed mass:
69755 Da
Δ319 Da
Mass
m/z
944.697
1029.244
997.484
894.287
862.208
1110.769
1163.556
1184.708
1226.23
804.635
831.458
1294.18
1343.96
1399.30
1457.51
1520.99
1556.40
c
Phosphate groups
+2
69596.2
+3
69674.9
+4
69755.0
+5
69837.1
+6
69915.1
69995.2
Mass
d
nucleosome
octamer
kDa
20 -
15 -
10 -
H3
H2B
H2A
H4
e
unmodified 12xNA
Aurora B - +
kDa
20 -
15 -
IB: H3S10p
f
12xNA with H3K9me3
Aurora B - + +
kDa
20 -
15 -
IB: H3S10p

Supplementary Figure 13. Analysis of Aurora B Kinase preparation. a) SDS-PAGE analysis of Aurora B samples. The middle lane is another unrelated sample. b) Mass spectrometry analysis of Aurora B. c) Mass spectrometry analysis of the heterogeneity within the peak. The difference in mass from the expected mass is consistent with autophosphorylation (increases in 80 Da increments per peak). d) SDS-PAGE analysis of non-phosphorylated histone octamers. e) H3S10p immunoblot of Aurora B kinase incubation with unmodified nucleosome arrays. Source data are provided as a source data file. f) H3S10p immunoblot of Aurora B kinase incubation with increasing amounts of H3K9me3 nucleosome arrays. Source data are provided as a source data file. The Western blot was repeated twice with similar results.

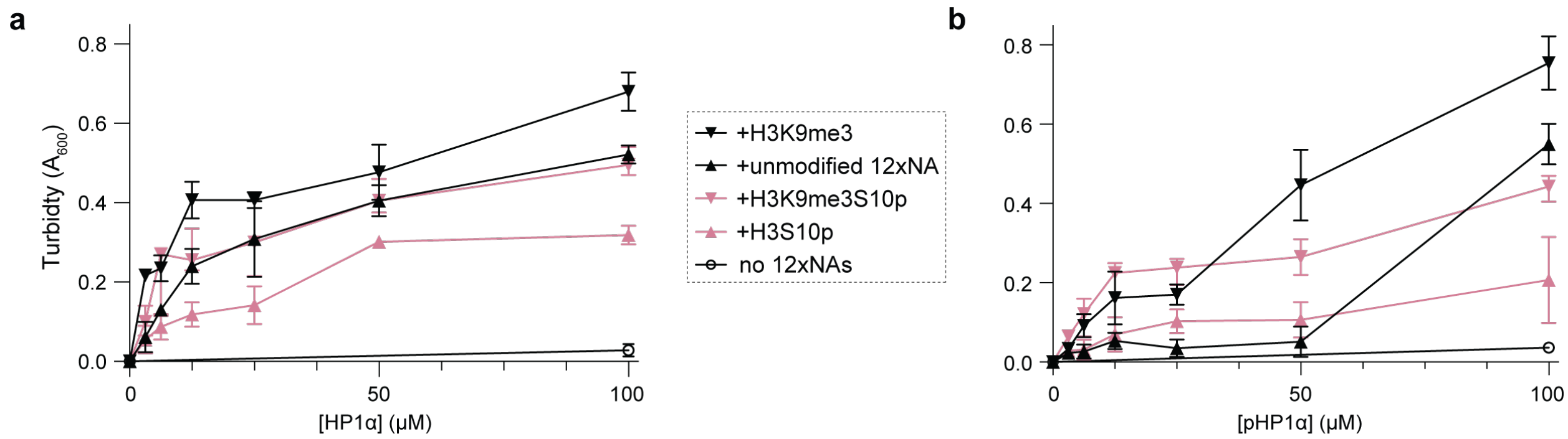

Supplementary Figure 14. Turbidity of HP1α/pHP1α in combination with nucleosome arrays modified by heterochromatin- or disease-associated histone PTMs. a) Turbidity at A600 of varying concentrations of HP1α with 40 nM 12mer nucleosome arrays (12xNA; 0.5 μM equivalent of mononucleosomes) with variable PTMs. The average of 3 independent replicates is shown, and error bars represent the standard deviation. b) Turbidity at A600 of varying concentrations of pHP1α with 40 nM 12mer nucleosome arrays (12xNA; 0.5 μM equivalent of mononucleosomes) with variable PTMs. The average of 3 independent replicates is shown, and error bars represent the standard deviation. Source data for all panels are provided as a source data file.

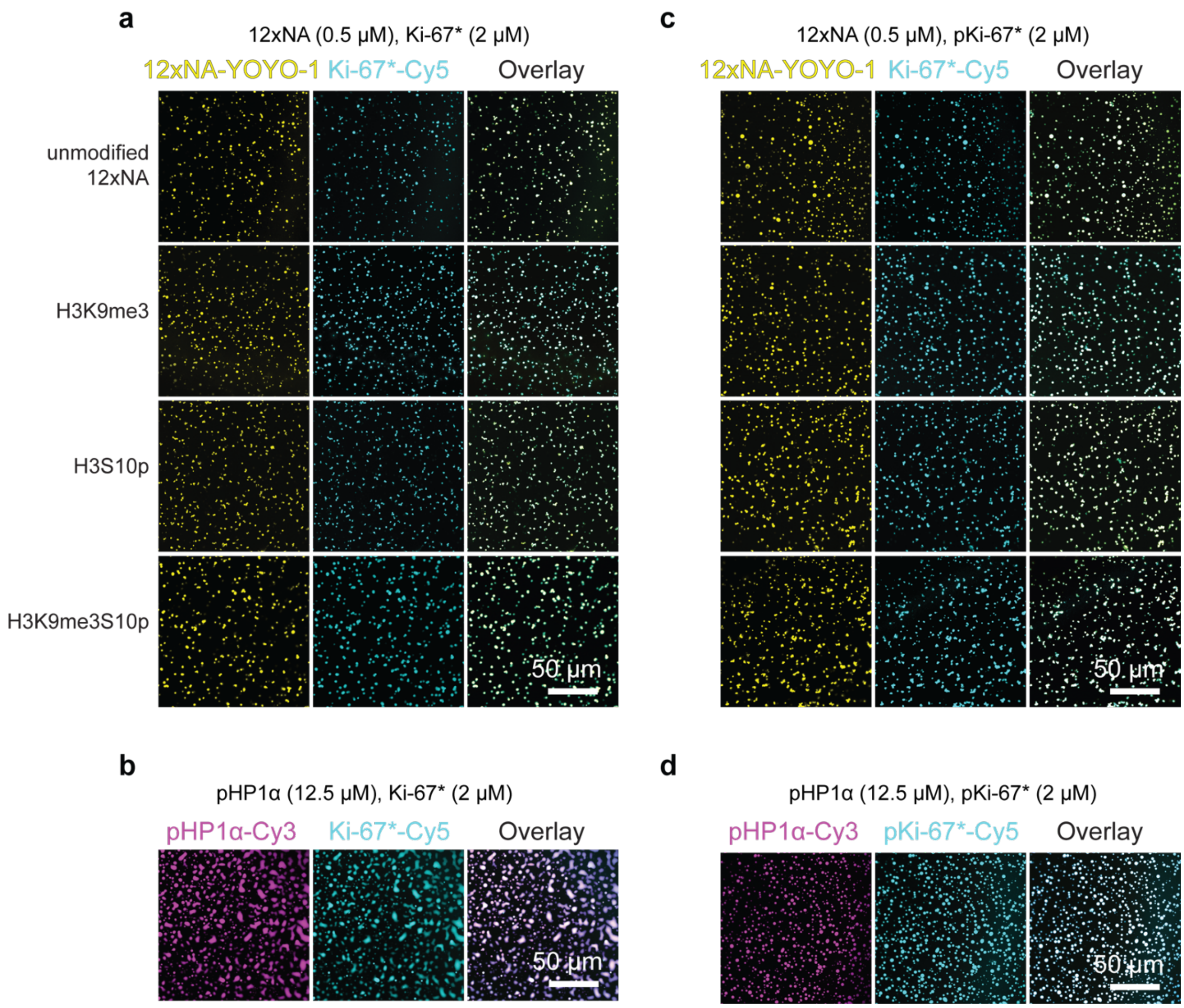

Supplementary Figure 15. Colocalization of Ki-67* with 12xNAs or pHP1α with variable PTMs. a) Confocal microscopy images of 2 μM Ki-67* and 40 nM 12xNA (0.5 μM equivalent nucleosomes) with variable PTMs, visualized with 5% Ki-67*-Cy5 and 0.4 μM YOYO-1. b) Confocal microscopy images of 12.5 μM pHP1α and 2 μM Ki-67*, visualized with 5% pHP1α-Cy3 and Ki-67*-Cy5. c) Confocal microscopy images of 2 μM pKi-67* and 40 nM 12xNA (0.5 μM equivalent nucleosomes) with variable PTMs, visualized with 5% pKi-67*-Cy5 and 0.4 μM YOYO-1. d) Confocal microscopy images of 12.5 μM pHP1α and 2 μM pKi-67*, visualized with 5% pHP1α-Cy3 and 5% pKi-67*-Cy5. Similar results were obtained from three independent batches of proteins.

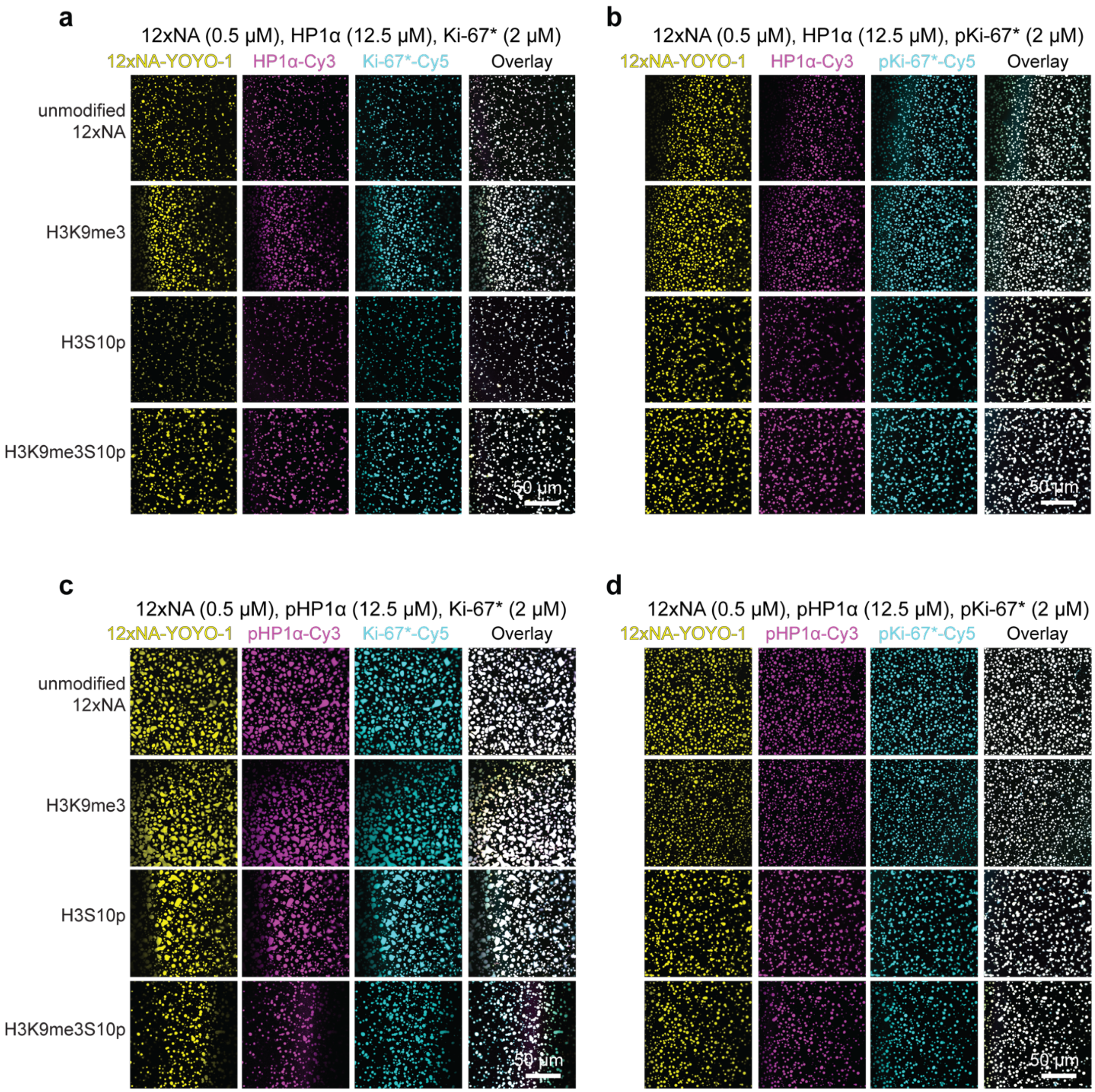

Supplementary Figure 16. Colocalization of Ki-67*/pKi-67*, 12xNAs, and HP1α/pHP1α with variable PTMs. a) Confocal microscopy images of 12.5 μM HP1α, 2 μM Ki-67*, and 40 nM 12xNA (0.5 μM equivalent nucleosomes) with variable PTMs, visualized with 5% HP1α-Cy3, 5% Ki-67*-Cy5, and 0.4 μM YOYO-1, respectively. b) Confocal microscopy images of 12.5 μM HP1α, 2 μM pKi-67*, and 40 nM 12xNA (0.5 μM equivalent nucleosomes) with variable PTMs, visualized with 5% HP1α-Cy3, 5% pKi-67*-Cy5, and 0.4 μM YOYO-1, respectively. c) Confocal microscopy images of 12.5 μM pHP1α, 2 μM Ki-67*, and 40 nM 12xNA (0.5 μM equivalent nucleosomes) with variable PTMs, visualized with 5% pHP1α-Cy3, 5% Ki-67*-Cy5, and 0.4 μM

YOYO-1, respectively. d) Confocal microscopy images of 12.5 μM pHP1α, 2 μM pKi-67*, and 40 nM 12xNA (0.5 μM equivalent nucleosomes) with variable PTMs, visualized with 5% pHP1α-Cy3, 5% pKi-67*-Cy5, and 0.4 μM YOYO-1, respectively. Similar results were obtained from three independent batches of proteins.

SUPPLEMENTARY TABLES

Supplementary Table 1. Maximum recovery (%) and half-time (s) parameters obtained in different FRAP experiments.

LLPS condition	Figure	Maximum recovery (%)	SEM	Half-life ($t_{1/2}$) (s)	95% CI Range	n
Ki-67* + 12xNA	6e,f	21.1	0.01	30.7	24.4 to 41.2	27
Ki-67* + DNA	6e,f	12.4	0.002	10.4	9.2 to 11.9	10
Ki-67* + RNA	6e,f	12.3	0.005	22.8	19.6 to 27.3	20
Ki-67* + pHP1α	6e,f	31.1	0.02	37.8	31.5 to 47.2	29
pKi-67* + 12xNA	6e,f	30.2	0.003	12.3	11.4 to 13.3	19
pKi-67* + DNA	6e,f	49.8	0.004	12.1	11.4 to 12.8	21
pKi-67* + RNA	6e,f	57.0	0.006	13.3	12.4 to 14.3	35
pKi-67* + pHP1α	6e,f	43.6	0.02	26.1	22.1 to 32.0	32

SEM – standard error of the mean

n – number of replicates

CI – confidence interval
