# Supplementary material for: Ki-67 regulates heterochromatin organization in neurons and forms condensates with heterochromatic components
Source: Nat Commun. 2026 Sep 8;17:10615. doi: 10.1038/s41467-026-77430-2 (PMC13649782; doi:10.1038/s41467-026-77430-2)
Supplement: Supplementary file 2 [file 41467_2026_77430_MOESM2_ESM.pdf]

Description of Additional Supplementary Files

Supplementary Data 1: Sequences of the Ki-67 constructs used in this study.
