# Supplementary material for: Ki-67 regulates heterochromatin organization in neurons and forms condensates with heterochromatic components
Source: Nat Commun. 2026 Sep 8;17:10615. doi: 10.1038/s41467-026-77430-2 (PMC13649782; doi:10.1038/s41467-026-77430-2)
Supplement: Supplementary file 3 [file 41467_2026_77430_MOESM4_ESM.pdf]

nature portfolio

Corresponding author(s): Galia Debelouchina, Cole Ferguson

Last updated by author(s): Aug 19, 2026

Reporting Summary

Nature Portfolio wishes to improve the reproducibility of the work that we publish. This form provides structure for consistency and transparency in reporting. For further information on Nature Portfolio policies, see our Editorial Policies and the Editorial Policy Checklist.

Statistics

For all statistical analyses, confirm that the following items are present in the figure legend, table legend, main text, or Methods section.

n/a	Confirmed	
☐	☒	The exact sample size (*n*) for each experimental group/condition, given as a discrete number and unit of measurement
☐	☒	A statement on whether measurements were taken from distinct samples or whether the same sample was measured repeatedly
☐	☒	The statistical test(s) used AND whether they are one- or two-sided<br>*Only common tests should be described solely by name; describe more complex techniques in the Methods section.*
☒	☐	A description of all covariates tested
☐	☒	A description of any assumptions or corrections, such as tests of normality and adjustment for multiple comparisons
☐	☒	A full description of the statistical parameters including central tendency (e.g. means) or other basic estimates (e.g. regression coefficient) AND variation (e.g. standard deviation) or associated estimates of uncertainty (e.g. confidence intervals)
☐	☒	For null hypothesis testing, the test statistic (e.g. *F*, *t*, *r*) with confidence intervals, effect sizes, degrees of freedom and *P* value noted<br>*Give P values as exact values whenever suitable.*
☒	☐	For Bayesian analysis, information on the choice of priors and Markov chain Monte Carlo settings
☒	☐	For hierarchical and complex designs, identification of the appropriate level for tests and full reporting of outcomes
☐	☒	Estimates of effect sizes (e.g. Cohen's *d*, Pearson's *r*), indicating how they were calculated

Our web collection on statistics for biologists contains articles on many of the points above.

Software and code

Policy information about availability of computer code

Data collection	Illumina Novaseq X Plus
Data analysis	Trimmomatic (v.0.39), Bowtie2 (v.2.3.4.1), SAMtools (v.1.14), Deeptools (v.3.5.0), SEACR (v.1.1), KaryoplotR (v.1.22.0), R (v.4.4.0), STAR v.2.7.9a, deepTools bamCoverage v.3.5.0., DESeq (v.1.44.0), featureCounts (v.1.6.0), IGV (broadinstitute.org/igv), ImageJ (v.2.9.0), NIS-elements (v.5.30.1), GraphPad Prism (v.10.4.1), Leica LAS X (v.3.5.7.23225), Bruker Topspin 4.0, POKY, HOOMD-Blue 4.7, VMD

For manuscripts utilizing custom algorithms or software that are central to the research but not yet described in published literature, software must be made available to editors and reviewers. We strongly encourage code deposition in a community repository (e.g. GitHub). See the Nature Portfolio guidelines for submitting code & software for further information.

Data

Policy information about availability of data

All manuscripts must include a data availability statement. This statement should provide the following information, where applicable:

- Accession codes, unique identifiers, or web links for publicly available datasets
- A description of any restrictions on data availability
- For clinical datasets or third party data, please ensure that the statement adheres to our policy

Source data are provided with this paper. CUT&RUN and RNA-seq data generated in this study have been deposited in the BioProject/SRA database under accession code PRJNA1242856 [https://www.ncbi.nlm.nih.gov/sra?term=SRP573657]. NMR assignments of the CSD-CTE homodimer of HP1α have been deposited in the BMRB under accession code 53698 [https://bmrb.io/data_library/summary/index.php?bmrbId=53698]. Mass spectrometry data has been deposited in MassIVE

archive under accession code MSV000102862 [https://massive.ucsd.edu/ProteoSAFe/dataset.jsp?accession=MSV000102862].

Research involving human participants, their data, or biological material

Policy information about studies with human participants or human data. See also policy information about sex, gender (identity/presentation), and sexual orientation and race, ethnicity and racism.

Reporting on sex and gender	*Use the terms sex (biological attribute) and gender (shaped by social and cultural circumstances) carefully in order to avoid confusing both terms. Indicate if findings apply to only one sex or gender; describe whether sex and gender were considered in study design; whether sex and/or gender was determined based on self-reporting or assigned and methods used.*<br>*Provide in the source data disaggregated sex and gender data, where this information has been collected, and if consent has been obtained for sharing of individual-level data; provide overall numbers in this Reporting Summary. Please state if this information has not been collected.*<br>*Report sex- and gender-based analyses where performed, justify reasons for lack of sex- and gender-based analysis.*
Reporting on race, ethnicity, or other socially relevant groupings	*Please specify the socially constructed or socially relevant categorization variable(s) used in your manuscript and explain why they were used. Please note that such variables should not be used as proxies for other socially constructed/relevant variables (for example, race or ethnicity should not be used as a proxy for socioeconomic status).*<br>*Provide clear definitions of the relevant terms used, how they were provided (by the participants/respondents, the researchers, or third parties), and the method(s) used to classify people into the different categories (e.g. self-report, census or administrative data, social media data, etc.)*<br>*Please provide details about how you controlled for confounding variables in your analyses.*
Population characteristics	*Describe the covariate-relevant population characteristics of the human research participants (e.g. age, genotypic information, past and current diagnosis and treatment categories). If you filled out the behavioural & social sciences study design questions and have nothing to add here, write "See above."*
Recruitment	*Describe how participants were recruited. Outline any potential self-selection bias or other biases that may be present and how these are likely to impact results.*
Ethics oversight	*Identify the organization(s) that approved the study protocol.*

Note that full information on the approval of the study protocol must also be provided in the manuscript.

Field-specific reporting

Please select the one below that is the best fit for your research. If you are not sure, read the appropriate sections before making your selection.

[x] Life sciences [ ] Behavioural & social sciences [ ] Ecological, evolutionary & environmental sciences

For a reference copy of the document with all sections, see nature.com/documents/nr-reporting-summary-flat.pdf

Life sciences study design

All studies must disclose on these points even when the disclosure is negative.

Sample size	No sample size calculation was performed. As this is an exploratory work, we only aimed to detect if there were any genes or binding sites with high fold change differences, thus using four replicates for RNA-seq and one replicate for CUT&RUN.
Data exclusions	No data was excluded from the analyses.
Replication	All in-cell transfection experiments were repeated at least twice. All in vitro assays were repeated at least three times, and each independent replicate included two technical replicates. The NMR titration experiments were performed with three different concentrations of Ki-67 to ensure the reproducibility of the observed intensity change patterns. Two biological replicates from individual experiments are presented in Figure 1c, d. Because Ki-67 mutants are born at sub-Mendelian ratios and the APC7 allele is recessive, typically only one mutant is obtained per litter, necessitating separate imaging experiments. Statistical comparisons were performed separately for each experiment because fluorescence intensities and segmentation thresholds varied between imaging sessions, making pooling of absolute measurements inappropriate. Within each experiment, control and mutant samples were acquired and analyzed under identical conditions; thus, these comparisons assess genotype-associated differences independently of experiment-specific imaging effects. Multiple experiments indicate whether the observed phenotype is reproducible across biological replicates.
Randomization	Animals were allocated to experimental versus control groups based on genotype. Wild-type littermates were used as controls for mutant mice.
Blinding	Processing and analysis of the samples used in immunofluorescence were performed blindly. In vitro experiments were not performed in a blinded fashion as sample preparation and/or analysis required knowledge of the precise mixture of the components.

Reporting for specific materials, systems and methods

We require information from authors about some types of materials, experimental systems and methods used in many studies. Here, indicate whether each material, system or method listed is relevant to your study. If you are not sure if a list item applies to your research, read the appropriate section before selecting a response.

Materials & experimental systems

n/a	Involved in the study
☐	☒ Antibodies
☐	☒ Eukaryotic cell lines
☒	☐ Palaeontology and archaeology
☐	☒ Animals and other organisms
☒	☐ Clinical data
☒	☐ Dual use research of concern
☒	☐ Plants

Methods

n/a	Involved in the study
☒	☐ ChIP-seq
☒	☐ Flow cytometry
☒	☐ MRI-based neuroimaging

Antibodies

Antibodies used

Rat monoclonal anti-Ki-67 (SolA15), Thermo Fisher Scientific, Cat #14-5698-82, RRID: AB_10854564
Rabbit monoclonal anti-Calbindin, Cell signaling technologies, Cat#13176, RRID:AB_2687400
Rabbit polyclonal anti-H3K9me3, Active Motif, Cat# 39065, RRID: AB_2793334
Rabbit monoclonal anti-H3K27me3, Cell signaling technologies, Cat#9733, RRID: AB_2616029
Rabbit monoclonal anti-H3K4me3, Cell signaling technologies, Cat#9751, RRID:AB_2616028
Rabbit polyclonal anti-H3K27ac, Active Motif, Cat#39133, RRID:AB_2561016
Rabbit anti-Phospho-Histone H3 (Ser10), Cell signaling technologies, Cat#9701
Goat anti-rabbit IgG, HRP-linked Antibody, Cell Signaling Technologies, Cat#7074
Donkey anti-rat IgG, AlexaFluor 594, Invitrogen, Cat#A-21209, RRID: AB_2535795
Donkey anti-rabbit IgG, AlexaFluor 488, Invitrogen, Cat#A-21206, RRID: AB_2535792

Validation

https://www.cellsignal.com/products/primary-antibodies/calbindin-d1i4q-xp-rabbit-mab/13176?srsltid=AfmBOopEfa8FT2Vm8DBAvwrWGNpsDPuOSDnTGVCMKYFa11d2Rm5r9-Fs
Calbindin antibody validated for immunofluorescence. 1:400 dilution used.
https://www.thermofisher.com/antibody/product/Ki-67-Antibody-clone-SolA15-Monoclonal/14-5698-82
Ki-67 antibody validated for immunofluorescence. 1:500 dilution used.
Alll CUT&RUN antibodies used at 1:75 dilution and were validated in a previous paper (https://journals.plos.org/plosgenetics/article?id=10.1371/journal.pgen.1011843)
Rabbit anti-Phospho-Histone H3 (Ser10), Cell signaling technologies, 1:1000 dilution
https://www.cellsignal.com/products/primary-antibodies/phospho-histone-h3-ser10-antibody/9701?srsltid=AfmBOoqg_4t2zpaese29_DzGJB7oPLP4-qTiEbdKHno2SEAJFq19g8ca

Eukaryotic cell lines

Policy information about cell lines and Sex and Gender in Research

Cell line source(s)	N2a cells were obtained from ATCC (CCL-131).
Authentication	Authenticity was guaranteed by the supplier.
Mycoplasma contamination	Cells were routinely checked in-house for mycoplasma using abcam Mycoplasma PCR detection kit and tested negative for mycoplasma contamination.
Commonly misidentified lines (See ICLAC register)	N/A

Animals and other research organisms

Policy information about studies involving animals; ARRIVE guidelines recommended for reporting animal research, and Sex and Gender in Research

Laboratory animals

ANAPC7 mutant mice, C57BL/6J, males, 7 days of age
Ki-67 mutant mice, C57BL/6J, males, 7, 10-11, 13 days of age
Animals were housed in a 12:12 light:dark cycle, ambient temperature and humidity. In general, we employed trio breeding. Pups were genotyped at P4-P5 by tail biopsy and weaned into sex-matched cages at P21 to be bred at 7-8 weeks.

Wild animals

The study did not involve wild animals.

Reporting on sex	Imaging experiments were repeated in both male and female mutants. Due to higher birth rates of male mutants, CUT&RUN and RNA-seq experiments were performed with only male pairs.
Field-collected samples	The study did not involve field-collected samples.
Ethics oversight	All animal procedures were approved by UCSD's Institutional Animal Care and Use Committee (IACUC) Office.

Note that full information on the approval of the study protocol must also be provided in the manuscript.

Plants

Seed stocks	*Report on the source of all seed stocks or other plant material used. If applicable, state the seed stock centre and catalogue number. If plant specimens were collected from the field, describe the collection location, date and sampling procedures.*
Novel plant genotypes	*Describe the methods by which all novel plant genotypes were produced. This includes those generated by transgenic approaches, gene editing, chemical/radiation-based mutagenesis and hybridization. For transgenic lines, describe the transformation method, the number of independent lines analyzed and the generation upon which experiments were performed. For gene-edited lines, describe the editor used, the endogenous sequence targeted for editing, the targeting guide RNA sequence (if applicable) and how the editor was applied.*
Authentication	*Describe any authentication procedures for each seed stock used or novel genotype generated. Describe any experiments used to assess the effect of a mutation and, where applicable, how potential secondary effects (e.g. second site T-DNA insertions, mosiacism, off-target gene editing) were examined.*
