# Supplementary material for: Ki-67 regulates heterochromatin organization in neurons and forms condensates with heterochromatic components
Source: Nat Commun. 2026 Sep 8;17:10615. doi: 10.1038/s41467-026-77430-2 (PMC13649782; doi:10.1038/s41467-026-77430-2)
Supplement: Supplementary file 4 [file 41467_2026_77430_MOESM5_ESM.pdf]

nature portfolio

Peer Review File

Ki-67 regulates heterochromatin organization in neurons and forms condensates with heterochromatic components

Corresponding Author: Professor Galia Debelouchina

Parts of this Peer Review File have been redacted as indicated to remove third-party material.

This file contains all reviewer reports in order by version, followed by all author rebuttals in order by version.

Version 0:

Reviewer comments:

Reviewer #1

(Remarks to the Author)
Abasi et al report an interesting link between Ki-67 properties of phase separation, heterochromatin maintenance and the neurodevelopment syndrome ANAPC7.

They start from the discovery that, in the ANAPC7 syndrome, Ki-767 (together with other important chromatin associated proteins) is up-regulated in neurones that have exited the cell cycle.
Their observation that these neurones have also expanded constitutive heterochromatin led them to investigate how Ki-67 contributed to this phenotype. They mainly use in vitro assays to dissect the biochemical and phase separation properties of Ki-67 domains, their interaction with chromatin arrays (modified and non-modified) and heterochromatin protein 1 (HP1) in its phospho and non- phospho status.
Altogether, this paper adds some novel aspects to Ki-67 biology (still quite obscure), provides additional explanations of how its biochemical properties may be important for chromatin organisation, reports a novel phenotype of Ki-67 null mice (this contrasts with what has been reported so far) and highlight another important link between neural biology and heterochromatin maintenance.
Because of all these factors, I believe it would represent a good publication suited for the Nature Communication readership as it encompassed several areas where important knowledge is still scarse.
However, I would recommend some revisions before its acceptance that clarify aspects that could mislead readership and generate confusion in the field.

My detailed comments are below:

1) My first concern is the title. I think it is too strong. The in vivo analyses do not show any staining with HP1 (they should provide some images and quantifications) using both the ANAPC7-/- neurones and the Ki-67-/- neurones. This is important also because previous work by the Fisher lab had shown that the three HP1 isoforms were still maintained in their normal localisation in the absence of Ki-67, suggesting that this is a Ki-67 independent behaviour. Moreover, the author should add a control of a Ki-67 mutant that cannot bind HP1 alpha for the key experiments in Figure 6. Here they demonstrate that nucleosomes are essential for HP1a to phase separate and adding Ki-67 provided a increase in turbidity but the mechanisms could be additional effects of two different phenomena. Similarly, the effect of pHP1 and Ki67 could be driven by some electrostatic interactions independent from the ability of Ki-67 to bind HP1(in fact Ki-67 phosphorylation seems to alleviate this clustering). It will be important to know also if these phosphorylation affect HP1 binding by other means. These are important clarification that the authors should make in order to avoid spreading strong assumption in this already complicated field.

2) The authors also claim that they demonstrate that disease-relevant histone modifications influence the phase separation properties. Again this statement is too strong. First of all, there is no evidence that these modifications are really relevant to the disease. There are not experiments showing that these modifications are causative of the disease; they are just found in the disease and maybe be just bystanders.

3) A strong link between Ki-67 and heterochromatin has been in the literature for some time and the authors should

acknowledge the literature that is directly relevant to their findings.
For example, starting from old reports (Bridger, Ian R. Kill & Peter Lichter Chromosome Research 1998) found association of pKi-67 with satellite DNA of the human genome in early G1 cells. Then Fisher et al, reported a link between Ki-67 and heterochromatin foci in human and mouse.
More recently, the Van Steensel group (EMBO reports 23: e55782 | 2022) showed a localisation and regulation of constitutive heterochromatin by Ki-67; lastly Stamatious et al, (doi: https://doi.org/10.1101/2024.04.29.591626) show a clear co-localisation between Ki-67 and centromeric chromatin and an expansion of the centromeric satellites areas upon Ki-67 degradation. None of those observation have provided a biochemical mechanisms but they all strengthen the observations provided in this study and they should be referred to.

4) The phenotype of the Ki-67 null mouse is also novel and interesting. It should be discussed and compared to the published one by Fisher et al where no difference in viability or genotype was observed but still defect in H3K9me3 distribution was present.

5) On this line, a discussion on the difference between the distribution of H3K9me3 should also be present. The authors report that there are no changes in histone modifications. This is in contrast with what reported in the Ki-67 KO mouse published by Fisher et al but aligns with the findings in human cells described by the Van Steensel group. These are important clarification in the field.

6) Some discussion is also needed to clarify an important point that puzzle me (and possibly the readers). The relationship between dynamic behaviour and phase separation. How do they explain that very dynamic interaction behaviour of pKi-67* is still generating condensates with the same initial properties.?

7) Finally, the link with the disease is still mysterious. At the end, there are no changes no gene expression and a discussion on how an increase in heterochromatin area could be a fundamental disease signature, is not analysed.

Minor comments:

1) For Figure 1A, 2A, and in general for Figures where Ki-67 localisation is shown, the authors should provide a higher magnification image. It is very difficult to get the details from the illustration presented.

2) A co-staining with HP1 would also be important to support the link between therse two proteins localisation during perturbation (in ANAPC7-/- and Ki-67-/-).

3) An explanation for the increased phase separation of pKi-67/pHP1 And H3S10ph should be provided. Ki-67 is highly phosphorylated in mitosis and clusters around the chromosomes, a phenomenon attributed to its phase separation properties. However, most of HP1 leaves chromatin in mitosis but it is not recruited to the chromosome periphery, actually a small fraction id retained at the pericentromeric regions. How do the authors reconcile their in vitro data with the biology of these proteins during cell cycle?

4) Statistical analyses. The authors have adopted -test for all the quantifications. While in some instances this can be appropriate, in others, and based on the dataset presented in the graph, the distributions do not appear normal. Therefore they should re-evaluate if a more appropriated statistical test should be employed (e.g. Mann-Whitney U test or a Wilcoxon test).

5) Line 216 should read " Ki-67's repeat domains were previously shown not to separate in isolation,.."

Reviewer #2

(Remarks to the Author)
In their manuscript entitled "Ki-67 promotes HP1α phase separation and regulates heterochromatin domains in the ANAPC7 neurodevelopmental syndrome" by Abasi et al, the authors study heterochromatin in mouse neurons that either lack Ki-67 or exhibit an accumulation of Ki-67 (ANAPC7 syndrome). They report a moderate increase in chromocenter sizes upon accumulation of Ki-67 (Anapc7 -/-) and a moderate increase in chromocenter number upon loss of Ki-67. Their images show that Ki-67 localizes to the periphery of chromocenters and relocalizes to nucleoli when cells are treated with 1,6-hexanediol. The authors then present a series of in vitro experiments to show that Ki-67 can form condensates after addition of DNA, RNA, nucleosome arrays or pHP1α. They also show that phosphorylation of Ki-67 enhances its mobility in condensates. Based on their results, the authors suggest that Ki-67 may act through a phase separation mechanism that involves heterochromatin components such as HP1.

While I find the question timely and interesting and although I find the mouse models relevant, I have doubts about the conclusions the authors make. In brief, I am wondering how the conclusion that Ki-67 acts by regulating HP1α phase separation fits to the observation that HP1α knockout mice do not show any phenotype and that cells lacking HP1α do not exhibit major heterochromatin perturbations. Also, I am not convinced that the in vitro experiments, where HP1α and Ki-67 colocalize in condensates, recapitulate the situation in cells, where Ki-67 localizes to the periphery of chromocenters while HP1 localizes throughout the chromocenters. I have a hard time imagining how two proteins would phase-separate together if they do not really localize to the same place.

Major points:

1. Mice lacking HP1α do not show any major phenotype (see for example PMIDs 21250545, 32020053) and mouse cells lacking HP1α still contain chromocenters with similar numbers and sizes (PMIDs 29166597, 37082862), suggesting that defects in HP1α function and phase separation can be compensated. This seems to be incompatible with the idea that phenotypes caused by loss/accumulation of Ki-67 arise from defects in HP1α function/phase separation. Rather, the effects on heterochromatin could be mediated by other proteins.

2. The heterochromatin alterations that the authors describe in Fig. 1 seem moderate, with 3 chromocenters more in the absence of Ki-67, which corresponds to the standard deviations in the individual conditions. Could the authors clarify how they think this difference makes a functional difference for the cell? Do they have any suggestion where the heterogeneity in the wildtype situation originates from (cell cycle? differentiation stage?) and if these factors could be affected in the mutant cells?

3. The authors put forward the model that HP1α phase-separates in the wildtype condition. It is unclear how the authors' conclusions about Ki-67 then fit to this model. If Ki-67 enhances phase separation of HP1α, then loss of Ki-67 should dissolve chromocenters (make them larger and more numerous) while accumulation of Ki-67 should reinforce them (make them smaller/more compact, and fewer)? This is not what is observed in Fig. 1C/D. It would be helpful to explicitly spell out how the phase separation mechanism the authors propose would affect the parameters quantified in Fig. 1C/D.

4. The authors use 1,6-hexanediol to draw conclusions about phase separation, although it is quite clear now that 1,6-hexanediol is toxic, dissolves nuclear pores (PMID 12509452), induces chromatin condensation (PMID 33536240), affects kinases and phosphatases (PMID 33814344), and perturbs hydrophobic interactions rather than dissolving condensates, which could also be formed via electrostatic interactions that are not affected by 1,6-hexanediol (PMID 36526633). I acknowledge that the authors have put a word of caution when interpreting their experiments with 1,6-hexanediol, but I would strongly advice to not make any conclusions about phase separation based on these experiments.

5. The authors suggest a model in which Ki-67 modulates HP1α phase separation and thereby affects chromocenter number and size. In their 1,6-hexanediol experiments, where Ki-67 leaves from the chromocenters, they actually have a tool to directly test this. Surprisingly, they did not quantify chromocenter sizes in these experiments. The prediction from the authors' model would be that the sizes change if I am not mistaken. Is this the case? If not, this speaks against a direct scaffolding role of Ki-67 in heterochromatin condensates.

6. The in vitro work is interesting, showing that Ki-67 can phase-separate when different molecules are added, including DNA, RNA, nucleosome arrays, HP1α/pHP1α. However, I am wondering if these experiments really recapitulate the situation in cells, where Ki-67 localizes to the periphery of DNA/nucleosome-dense chromocenters (see first column in Fig. 2A, where Ki-67 forms rings around Hoechst-stained foci, most pronounced in Anapc7 -/- cells but also in control cells. Line profiles could help to better visualize this). In the in vitro experiments, no Ki-67 ring structures are observed upon addition of DNA, HP1α and nucleosome arrays, see for example Fig. 3B-E, Fig. 4D). From my perspective, it is crucial to explain this difference, as my naive interpretation would be that these components form condensates in vitro but not in cells (hence, lack of colocalization in foci in cells but instead Ki-67 rings around Hoechst foci). To clarify the relative localization of the individual components in the in vitro assays, it would help to also label DNA, RNA, nucleosomes arrays in addition to Ki-67, and acquire multi-color images.

7. I am surprised that previous work on Ki-67 and heterochromatin regulation is not discussed/cited, including PMID 26949251 and 37082862. Rather, the authors write in the Abstract that the role of Ki-67 in regulating heterochromatin during interphase is poorly understood. I am not sure if this accurately reflects the current state of the art.

Minor points:

- RNA-seq: It would be interesting to know if the levels of major satellite transcripts are altered.

- I would not refer to a fragment of a protein as an "idealized" version of the protein (line 192).

- For the in vitro condensates, it does not seem to be entirely clear which component plays the role of driver/scaffold and which component that of a client. Nucleosome arrays can form condensates on their own if the solubility in the buffer is low enough (see for example PMID 31543265), so condensates made with nucleosome arrays and any protein could arise from the latter binding to the arrays and reducing their solubility, as opposed the protein itself undergoing phase separation. The effect of histone PTMs could then be to reduce the solubility of the arrays or to modulate the affinity of the binder that makes it more/less soluble. Maybe the authors want to take this into account when discussing their results.

Reviewer #3

(Remarks to the Author)
This work presents new findings spanning animal, neuron, sequencing, and biochemical studies on the role of Ki-67 in heterochromatin. First, new mice models are created that remove a ubiquitin ligase resulting in accumulation of Ki67

(ANAPC7-/-, mimicking genetic disease with heterochromatin defects) and, for comparison, Ki67 deletion. These mice show interesting phenotypic and mutant viability differences and neurons from these mice are analyzed showing disrupted heterochromatin. Heterochromatin marks were not different in these cells, suggesting a different reason for the change in organization, as suggested by previous research regarding the effect of Ki67 on chromatin directly. Ki67 localization is altered by 1,6 hexanediol, going to nucleolus, implying some change in weak interactions that this solvent can alter. In vitro biochemical assays show that a minimal Ki67 protein phase separates with heterochromatin components – nucleosome arrays, DNA, RNA, and HP1alpha. Ki67* phosphorylation alters this phase separation while nucleosome modifications make small changes to the behavior. The work is exciting due in large part to the animal and cellular data as well as the fundamental link made to the biochemical experiments – this link could be strengthened further. The lack of genomic data accounting for the phenotypes is demonstrated but a better hypothesis could be put forward (and tested). Additionally, a big gap between the biochemical and cellular data leave some questions, and the biochemical data are extensive but the connection to the cellular features is not so clear and the final sections could be shortened to strengthen the work. Experiments testing the connections made using mutagenesis in vitro and then in cells would help show the proposed features in vitro really explain the behavior in mouse/cells.

Major
Does it change the view of the field if the chromatin state is drastically changed but the markers are not? This should be expanded upon in the text.

It seems like a big jump to go from “not detect substantial changes in transcriptome” to “phase separation of nuclear components”. I think the connection should be made much more clear. I don’t think the biochemical experiments are really explaining the lack of difference. Also, could the changes in the cells be checked earlier for example immediately after fertilization or with in vitro fertilization on zygotes rather than in neurons?

I understand that expressing a 3000+ residue protein is probably not so feasible in bacteria, but the authors could try in bacteria and in insect or mammalian cells to do so. It is not clear why the FHA domain is not included and surprising that it doesn’t make a difference. The justification for the exclusion of domains should be better. Perhaps adding each one and showing they don’t do anything in the assays?

Quantification of the images of biochemical assays could help improve insight from this work.

How is 1,6 hexanediol working in cells? The figure showing the difference should be more clear and have quantification of the difference in panel A and not just the nucleolar localization if possible. Does it do anything in the biochemical assays? Recent work on hexanediol says it doesn’t work on charged condensates (https://pubmed.ncbi.nlm.nih.gov/40281357/ from Fawzi and coworkers), so what is it doing here? It sounds interesting and useful to show this, but the mechanism may not be due to Ki67 directly but rather to other cellular components and this should be commented on and explored in vitro.

The real strength of the biochemical assays is showing the condensation and the co-condensation. The meager effects of most of the modifications could be addressed much more briefly and placed in SI in my opinion. This would streamline the manuscript considerably. The lack of DNA binding domain and other domains from Ki67* (see comment above) is concerning and could be addressed. Cellular studies with domain deletions could also be performed – the assay on Ki67* in cells is good but co-localization with Ki67 endogenous should be demonstrated.

The bleach regions appear different in that some drops show apparent bleaching of the region surrounding. Therefore, is the normalization done to a region in the same drop that is outside the bleach region? Or is it normalized to a distinct droplet? If the latter, then there is no way the bleach region will recover above the unbleached region in same droplet. So larger droplets (higher concentrations) could be performed to remove such complications.

As described above, mutants that disrupt functions/localizations in cells could be compared in vitro to see if they disrupt phase separation. This would strengthen the claim that Ki67 is biologically relevant partner through phase separation. For example, it is not surprising that changing the charge by phosphorylation alters the phase separation of, or interactions with, something that is highly positively charged. Could variants altering the charge or modification sites in cells be performed to show that the changes in test tubes are found in cells?

The statement about “in vivo data support the conclusion … phase separation” could be improved by commenting in parallel on the in vitro work in the same sentence / paragraph. That is the strength here.

Minor
Abstract:
“defective proteomsomal degradation” – of what?

The hypothesis that “Ki67 cooperates with HP1alpha …” articulated in the introduction should be explicitly mentioned here

“that appears robust to” vague and weak, rephrase

“suggest that Ki67 may influence” weak – perhaps just “may influence” or “suggest”

Introduction:
“at smaller scales” confusing
“and/or epigenetic state” not clear
“multiplicity of neuronal functions” give some examples and maybe “myriad” or “many” is correct word
“unsurprising” used in two sentences in a row!
“Ki67 accumulates within” mention how this was demonstrated?
“Compared to its role in mitosis” rephrase to make more clear
“which is implicated to play a role” “which plays a role” “which is thought to play a role”

Results
Explicitly state that the human condition is recessive(?)
That the Sox2 promoter Cre results in global knockout should be demonstrated or at least a reference for this method as seems that it is not the standard way to generate a null.

“a membrane free organelle that forms” delete this and say “HC may be a distinct nuclear compartment formed through phase separation”

The domain structure in Fig3A should be shown in figure 2 perhaps and the features that are common to phase separation should be shown aligned with the domain structure

“its charge of +33 at pH 7.2” this is predicted charge and doesn’t account for pKa changes probably. add “predicted”

Ki-67* is also called R12,2-LRD which is confusing to use both, or am I not correct (are these the same?)

The text could mention if the pHP1alpha is pS/pT/pY etc and how many sites approx

Version 1:

Reviewer comments:

Reviewer #1

(Remarks to the Author)
In this revised manuscript, Adasi et al have spend quite some efforts in addressing the referee’s comments.

I appreciate they took into account several suggestions and clarified points that could have been confusing in the previous version.

I appreciate that some requests (although still standing as important) have proven technically challenging and cannot be addresses in full.

Overall, I am happy with the changes and I am in favour of accepting the manuscript in this present version.

Only one point that should be modified is that the reference “Stamatiou, K. et al. Multi-omic analyses reveal a differential contribution of chromatin-associated
PP1 holoenzymes to mitotic exit and G1 re-establishment. bioRxiv, 2024.2004.2029.591626
(2024). https://doi.org/10.1101/2024.04.29.591626

Now it is published and should be quoted as DOI: 10.1016/j.devcel.2026.02.016

Reviewer #2

(Remarks to the Author)
The authors have revised their manuscript. Unfortunately, they did not address most of my points. While this paper presents some interesting data on Ki-67, for example the effect of Ki-67 depletion on the viability and development of mice, and the lack of any effect on the transcriptome and the epigenetic state, I am not convinced that the central model of the paper is sufficiently supported, namely the model that Ki-67 organizes heterochromatin via phase-separation.

- I do not see any impressive colocalization between Ki-67 and chromocenters, which would be required for such a phase separation model (see for example Fig. S4a - Hoechst and Ki-67 do not overlap). There is a colocalization in Fig. S8, when cells are fixed with methanol:acetone, but it is clear that the nuclear organization is heavily perturbed under these conditions, as the authors state in the rebuttal letter, so that this colocalization is not representative of a living cell.
I do believe that some Ki-67 binds to heterochromatin, which has also been documented in the previous literature (see

references mentioned by me and the other reviewers in the first round of review). However, for condensate formation via phase separation, partial colocalization is not enough, because the concentration jumps where the dense phase forms, leading to a very prominent colocalization, not a partial colocalization, and a pronounced phase boundary between the coexisting phases. Partial colocalization and mixed localization at heterochromatin and other sites (nucleoli) is much more consistent with a model in which Ki-67 does not phase-separate but bind with some moderate affinity to heterochromatin sequences (and to sequences in nucleoli).

- I don't see statements like "Perturbing Ki-67 in differentiating neurons disrupts heterochromatin organization" supported, just because there is a moderate change in chromocenter numbers, within the standard deviation of chromocenter numbers observed in unperturbed cells. Without Ki-67, chromocenters look very similar to those in wildtype cells (Fig. 1a), the epigenetic state and the transcription at heterochromatin does not change. To me, it remains unclear if Ki-67 has a major impact on chromocenter organization and heterochromatin function. I am also missing any serious attempt to explain the mouse phenotypes in terms of viability and development by the moderate changes seen at heterochromatin.

- The authors insist on interpreting experiments with hexanediol and on drawing conclusions about cells based on in vitro experiments. I am not convinced that this can be done here. The in vitro system is highly simplified and behaves differently from cells (HP1/Ki-67/chromatin perfectly co-localize, while in cells they don't - this is not a detail, because co-localization is at the core of a phase separation mechanism). Hexanediol is known to have plenty of side effects and to be toxic.
I have no doubt that a large disordered protein like Ki-67 phase-separates in vitro, and I am also not surprised that it binds chromatin arrays, as it is a chromatin-binding protein. However, I don't see that these data can be used to justify the model that Ki-67 organizes heterochromatin in cells through phase-separation, especially due to the mild phenotypes seen upon depletion in cells (most chromocenters are still there) and the poor colocalization between Ki-67 and chromocenters in cells.

Reviewer #3

(Remarks to the Author)
The authors have addressed my comments throughly in their replies and updated manuscript.

Version 2:

Reviewer comments:

Reviewer #2

(Remarks to the Author)
The authors have revised their manuscript and now acknowledge more clearly the limitations and open questions. I think this improves the paper. I have two remaining points below that the authors may want to consider, but I do not want to delay the process given that the other reviewers appear satisfied.

1. For the colocalization analysis in Fig. S4f, it would be helpful to also look at the colocalization between HP1alpha and Ki-67 and between HP1alpha and Hoechst. With such an analysis, one could put the other numbers (Pearson R values for Ki-67/nucleolin etc) into context. At the end, a large part of the paper and the resulting model revolve around Ki-67 phase-separating with HP1alpha, and I would find it therefore important to quantify this in cells. In the best case, this would be via immunostaining against HP1alpha, otherwise via co-expression of HP1alpha tagged with GFP or some other fluorescent protein.

2. The observation that Ki-67-positive particles undergo fission is cited in support of a phase separation model (Discussion, page 17). However, classical phase separation theory does not predict fission: condensates are expected to grow over time, be it through Brownian coalescence or Ostwald ripening, toward the energetically favorable state of reduced interfacial area. Fission can occur in phase-separating systems, but it requires invoking mechanisms beyond the classical phase separation theory itself, such as active processes that mechanically fragment condensates, potentially an actively imposed change in condensate composition, etc. Since phase separation is an inherently passive process, fission would need to be explained by extensions or modifications of the phase separation model, not by that model alone. But obviously, other models could also be extended to explain fission, so I would not cite this observation in support of phase separation.

Open Access This Peer Review File is licensed under a Creative Commons Attribution 4.0 International License, which permits use, sharing, adaptation, distribution and reproduction in any medium or format, as long as you give appropriate credit to the original author(s) and the source, provide a link to the Creative Commons license, and indicate if changes were made.
In cases where reviewers are anonymous, credit should be given to 'Anonymous Referee' and the source.
The images or other third party material in this Peer Review File are included in the article's Creative Commons license, unless indicated otherwise in a credit line to the material. If material is not included in the article's Creative Commons license and your intended use is not permitted by statutory regulation or exceeds the permitted use, you will need to obtain permission directly from the copyright holder.
To view a copy of this license, visit https://creativecommons.org/licenses/by/4.0/

Response to the reviewer comments

We are grateful to the reviewers for their thoughtful and detailed comments, which have helped us significantly improve the manuscript. We have done our best to address their comments, resulting in substantial changes in the manuscript, including a new results section that contains structural and computational data, as well as a new version of the discussion to elevate previously published work and present a more unified model of the role of Ki-67 in heterochromatin organization in the context of neurodevelopment. After thorough review of the reviewers' suggestions and questions, we felt that some topics would warrant a much more detailed investigation (e.g. specifics regarding the phase separation mechanism in the multicomponent assemblies described in the manuscript or detailed characterization of the interactions between Ki-67 and HP1α). Rather than detract from the main messages of the manuscript (Ki-67 contributes to heterochromatin organization in neurons and can phase separate with heterochromatin components), we hope to pursue these topics in much more detail in the future. We are grateful for all of the ideas the reviewers have given on these topics. Below, we present our detailed responses to all comments.

Reviewer #1 (Remarks to the Author):

Abasi et al report an interesting link between Ki-67 properties of phase separation, heterochromatin maintenance and the neurodevelopment syndrome ANAPC7. They start from the discovery that, in the ANAPC7 syndrome, Ki-767 (together with other important chromatin associated proteins) is up-regulated in neurones that have exited the cell cycle. Their observation that these neurones have also expanded constitutive heterochromatin led them to investigate how Ki-67 contributed to this phenotype. They mainly use in vitro assays to dissect the biochemical and phase separation properties of Ki-67 domains, their interaction with chromatin arrays (modified and non-modified) and heterochromatin protein 1 (HP1) in its phospho and non- phospho status.
Altogether, this paper adds some novel aspects to Ki-67 biology (still quite obscure), provides additional explanations of how its biochemical properties may be important for chromatin organisation, reports a novel phenotype of Ki-67 null mice (this contrasts with what has been reported so far) and highlight another important link between neural biology and heterochromatin maintenance.
Because of all these factors, I believe it would represent a good publication suited for the Nature Communication readership as it encompassed several areas where important knowledge is still scarse.
However, I would recommend some revisions before its acceptance that clarify aspects that could mislead readership and generate confusion in the field.

My detailed comments are below:

1) My first concern is the title. I think it is too strong.

We have changed the title from “Ki-67 promotes HP1α phase separation and regulates heterochromatin domains in the *ANAPC7* neurodevelopmental syndrome” to “Ki-67 regulates heterochromatin organization in neurons and forms condensates with heterochromatin components”. We appreciate the reviewer’s concerns and believe that the new title better represents the conclusions of our study.

The in vivo analyses do not show any staining with HP1 (they should provide some images and quantifications) using both the ANAPC7-/- neurones and the Ki-67-/- neurones. This is important also because previous work by the Fisher lab had shown that the three HP1 isoforms were still maintained in their normal localisation in the absence of Ki-67, suggesting that this is a Ki-67 independent behaviour.

We invested a great deal of time and effort into this important critique. We tested several commercial antibodies to HP1α with limited success, attempting many different fixation and detergent conditions. The CST reagent worked to a degree in immunofluorescence, but detection required methanol/acetone fixation, which distorts the nuclear architecture of primary neurons. Ki-67 immunofluorescence does not work nearly as well with methanol/acetone fixation as with paraformaldehyde. In the resubmitted version we include images of rare publication quality fields with decent preservation of nuclear morphology and Ki-67 immunoreactivity (**Figure S8**). These demonstrate colocalization of HP1α with Ki-67 within chromocenters. However, the quality of these images is simply not good enough for quantitation. It is likely that Fisher’s group had better results because they used fibroblasts, which do not suffer from the same frailty as neurons when subjected to fixation with organic solvents.

Moreover, the author should add a control of a Ki-67 mutant that cannot bind HP1 alpha for the key experiments in Figure 6. Here they demonstrate that nucleosomes are essential for HP1a to phase separate and adding Ki-67 provided a increase in turbidity but the mechanisms could be additional effects of two different phenomena. Similarly, the effect of pHP1 and Ki67 could be driven by some electrostatic interactions independent from the ability of Ki-67 to bind HP1(in fact Ki-67 phosphorylation seems to alleviate this clustering). It will be important to know also if these phosphorylation affect HP1 binding by other means. These are important clarification that the authors should make in order to avoid spreading strong assumption in this already complicated field.

We also spent a significant amount of time trying to identify a Ki-67* mutant that does not bind HP1α. We know that the LR domain is required for co-localization to chromocenters (see new **Figure 3**), that this domain contains several putative HAC motifs (HP1a access codes or PXVXL-like motifs), and that it interacts with HP1α at the CSD-CSD dimer interface where known HP1α binding partners interact (see new **Figure 7**). However, mutations in the putative HAC motifs on Ki-67* were insufficient to abolish binding to HP1α (see new **Figure S12d,e**), implying more complexity in the binding interactions. To gain more insights into these puzzling observations, we performed coarse-grained MD simulations (see new **Figure 7**) in the context

of phase separation. The simulations suggest that the charge patterning in Ki-67* results in a complex network of multivalent electrostatic interactions with the entire HP1ɑ sequence. When HP1ɑ is phosphorylated on the N-terminus, the electrostatic interactions become much more focused on the HP1ɑ NTE, although they still involve multiple sites on the Ki-67* construct. Therefore, we conclude that Ki-67*/HP1ɑ phase separation is driven by a complex network of electrostatic interactions, and it would be difficult to abolish this without substantial mutations to the Ki-67* sequence. We will be following up on these observations in a future manuscript, as we have similar results with other HP1ɑ binding partners. To address this important point in the current manuscript, we have added a new results section and figures (**Figure 7, Figure S12**) where we present and discuss the binding assays, the NMR interaction data, and the simulations.

2) The authors also claim that they demonstrate that disease-relevant histone modifications influence the phase separation properties. Again this statement is too strong. First of all, there is no evidence that these modifications are really relevant to the disease. There are not experiments showing that these modifications are causative of the disease; they are just found in the disease and maybe be just bystanders.

We agree with the reviewer. We do not have evidence that H3S10p is pathogenic, just observations that it is enriched in the *ANAPC7-/-* case. We have removed any claims regarding its potential pathogenic role, however, we still believe it is important to include in our analysis, as its presence on the H3 tail should affect HP1ɑ binding to nucleosomes. However, as discussed in the manuscript, it appears that the complex interaction networks in Ki-67*/(p)HP1ɑ/nucleosome arrays condensates can compensate for its presence.

3) A strong link between Ki-67 and heterochromatin has been in the literature for some time and the authors should acknowledge the literature that is directly relevant to their findings.
For example, starting from old reports (Bridger, Ian R. Kill & Peter Lichter Chromosome Research 1998) found association of pKi-67 with satellite DNA of the human genome in early G1 cells. Then Fisher et al, reported a link between Ki-67 and heterochromatin foci in human and mouse. More recently, the Van Steensel group (EMBO reports 23: e55782 | 2022) showed a localisation and regulation of constitutive heterochromatin by Ki-67; lastly Stamatious et al, (doi: https://doi.org/10.1101/2024.04.29.591626) show a clear co-localisation between Ki-67 and centromeric chromatin and an expansion of the centromeric satellites areas upon Ki-67 degradation. None of those observation have provided a biochemical mechanisms but they all strengthen the observations provided in this study and they should be referred to.

We appreciate this conscientious feedback from this knowledgeable reviewer. In the resubmitted version we make a concerted effort to do a better job doing justice to these foundational studies, and we specifically address them in the new version of the discussion section.

4) The phenotype of the Ki-67 null mouse is also novel and interesting. It should be discussed

and compared to the published one by Fisher et al where no difference in viability or genotype was observed but still defect in H3K9me3 distribution was present.

The Ferguson lab imported the Ki-67 point mutant from the Fisher lab with the intention of studying neurons in the brain of these mice. However, we were disappointed to observe that neurons from homozygous mutants exhibited no reduction of Ki-67 in immunofluorescence using antibodies targeting the Ki-67 repeat domains (the epitope bound by most monoclonal antibodies against Ki-67). Preservation of Ki-67 in this model probably arises from the manner in which the Fisher lab made this mutation, which was to mutate the start codon in exon 1. The Mki67 gene contains an alternative start codon in exon 4 lying well upstream of the massive exon 12 that encodes the Ki-67 repeat domains and the last few exons encoding the C-terminal LR domain. This was one of the major motivations for us to make a true knockout allele, in this case one that targets exon 4 and actually inactivates the Mki67 gene. Our immunofluorescence experiments demonstrate elimination of Ki-67 in the cells of interest, and very likely throughout the whole body. Elimination of Ki-67, versus the mild hypomorphic allele reported by Fisher's group, is likely to account for differences in viability.

5) On this line, a discussion on the difference between the distribution of H3K9me3 should also be present. The authors report that there are no changes in histone modifications. This is in contrast with what reported in the Ki-67 KO mouse published by Fisher et al but aligns with the findings in human cells described by the Van Steensel group. These are important clarification in the field.

In mouse cells, H3K9me3 overlaps precisely with chromocenters. The Fisher lab examines H3K9me3 using spatial distribution imaging approaches in cell lines. In our case we examine heterochromatin by staining chromocenters, observing Ki-67-dependent differences in their number and distribution. We also observed changes in constitutive heterochromatin using imaging approaches. What we fail to find are changes in the abundance or genomic distribution of H3K9me3 using next-generation sequencing chromatin mapping methods. These are not mutually exclusive results. Ki-67 could be responsible for the spatial organization of heterochromatin without being required for its epigenetic state, which is likely to be determined by specific sequence characteristics of constitutive heterochromatin. We have attempted to clarify our interpretation of these results in the resubmitted version.

6) Some discussion is also needed to clarify an important point that puzzle me (and possibly the readers). The relationship between dynamic behaviour and phase separation. How do they explain that very dynamic interaction behaviour of pKi-67* is still generating condensates with the same initial properties.?

To keep the manuscript more focused, we have avoided extensive discussion of the exact mechanisms of phase separation as they can be quite complicated, especially in the multicomponent systems we investigate here. We plan to pursue this further in a dedicated future manuscript, but we suspect that it all comes down to refocusing of the interactions. The figure below shows the charge patterns for Ki-67* and pKi-67* (assuming all known phosphorylation sites are occupied). Upon phosphorylation, which is primarily concentrated in

the repeat region, there is extensive reorganization of the charge patterns, resulting in a predominantly negative repeat region, and a predominantly positively charged LRD. While this arrangement may reduce the interactions with negatively charged components, such as DNA, it may increase interactions with the positively charged histone tails, or promote more extensive pKi-67*/pKi-67* contacts, thus changing the dynamic properties of the protein within the condensate. We also note that since we use recombinant AuroraB kinase to phosphorylate Ki-67*, there is significant heterogeneity in the samples, and we are likely not observing the full range of dynamic behavior.

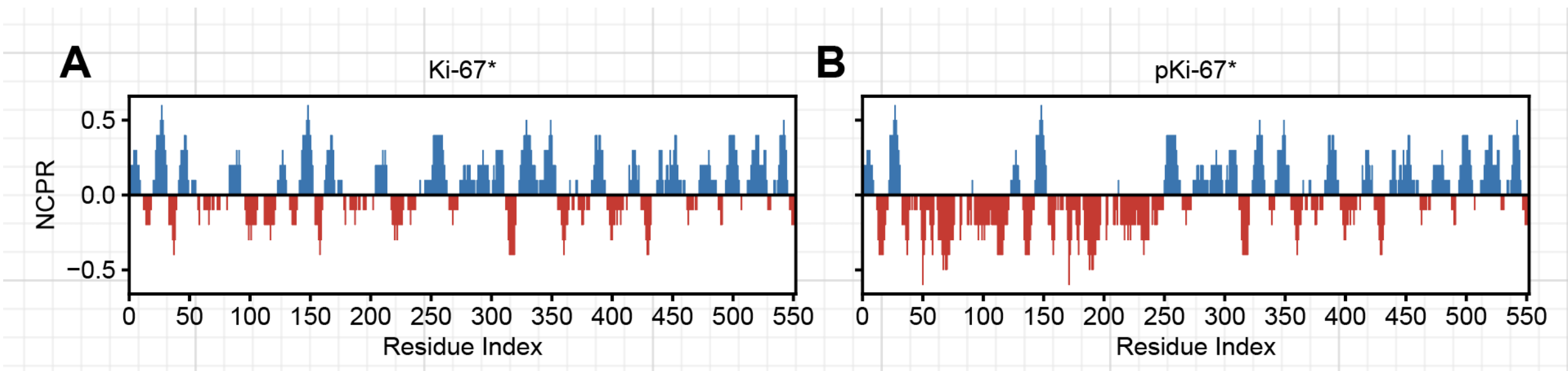

7) Finally, the link with the disease is still mysterious. At the end, there are no changes no gene expression and a discussion on how an increase in heterochromatin area could be a fundamental disease signature, is not analysed.

We agree with the reviewer. Defining how pathologic molecular and cellular mechanisms in the *ANAPC7* syndrome contribute to atypical neurodevelopment has been challenging. In the resubmitted version, we de-emphasize the disease connection a bit and instead focus on some fundamental questions related to Ki-67's function/mechanism in heterochromatin compartments. We speculate that changes in phase separation propensity in the nucleus can redistribute chromatin proteins leading to molecular changes at a specific location and at a specific time. We also cannot exclude a potential role for RNA in this process. We hope to address these hypotheses in future studies.

Minor comments:

1) For Figure 1A, 2A, and in general for Figures where Ki-67 localisation is shown, the authors should provide a higher magnification image. It is very difficult to get the details from the illustration presented.

In the resubmitted version, we provide sharper images of Ki-67 staining in **Figure 1**, **Figure S4**, **Figure S5**, **Figure S8**, including higher magnification images.

2) A co-staining with HP1 would also be important to support the link between these two proteins localisation during perturbation (in ANAPC7-/- and Ki-67-/-).

We have included this in our new **Figure S8**, although we encountered the technical challenges described above that prevented quantitation of HP1α. Therefore we could not examine how overabundance of Ki-67 or Ki-67 loss impacts the size and distribution of HP1α-positive heterochromatin in the nuclei of neurons.

3) An explanation for the increased phase separation of pKi-67/pHP1 And H3S10ph should be provided. Ki-67 is highly phosphorylated in mitosis and clusters around the chromosomes, a phenomenon attributed to its phase separation properties. However, most of HP1 leaves chromatin in mitosis but it is not recruited to the chromosome periphery, actually a small fraction id retained at the pericentromeric regions. How do the authors reconcile their in vitro data with the biology of these proteins during cell cycle?

We interpret the increased phase separation of pKi-67/pHP1α and H3S10p with caution. We used recombinant Aurora B to phosphorylate our nucleosome arrays *in vitro*, which leads to incomplete phosphorylation and some variability from sample to sample, as reflected in the larger error bar for the biological replicates in this assay. Therefore, it is possible that this increase is simply due to incomplete phosphorylation. We have added discussion of this issue in the text.

4) Statistical analyses. The authors have adopted -test for all the quantifications. While in some instances this can be appropriate, in others, and based on the dataset presented in the graph, the distributions do not appear normal. Therefore they should re-evaluate if a more appropriated statistical test should be employed (e.g. Mann-Whitney U test or a Wilcoxon test).

We have now analyzed non-normal data using the Mann-Whitney U test.

5) Line 216 should read " Ki-67's repeat domains were previously shown not to separate in isolation,.."

Previous literature (Ref. 39) has shown that the repeat domains can undergo phase separation, but only if crowding agents are added. To make this more clear and to take into account the reviewer's comment, we have changed the sentence to (now lines 236 - 238):

"While Ki-67* has previously been shown to undergo phase separation on its own in the presence of crowding agents[39], here we focused on its ability to form condensates with heterochromatin components without the addition of molecular crowders."

Reviewer #2 (Remarks to the Author):

In their manuscript entitled "Ki-67 promotes HP1α phase separation and regulates heterochromatin domains in the ANAPC7 neurodevelopmental syndrome" by Abasi et al, the authors study heterochromatin in mouse neurons that either lack Ki-67 or exhibit an accumulation of Ki-67 (ANAPC7 syndrome). They report a moderate increase in chromocenter

sizes upon accumulation of Ki-67 (Anapc7 -/-) and a moderate increase in chromocenter number upon loss of Ki-67. Their images show that Ki-67 localizes to the periphery of chromocenters and relocalizes to nucleoli when cells are treated with 1,6-hexanediol. The authors then present a series of in vitro experiments to show that Ki-67 can form condensates after addition of DNA, RNA, nucleosome arrays or pHP1α. They also show that phosphorylation of Ki-67 enhances its mobility in condensates. Based on their results, the authors suggest that Ki-67 may act through a phase separation mechanism that involves heterochromatin components such as HP1.

While I find the question timely and interesting and although I find the mouse models relevant, I have doubts about the conclusions the authors make. In brief, I am wondering how the conclusion that Ki-67 acts by regulating HP1α phase separation fits to the observation that HP1α knockout mice do not show any phenotype and that cells lacking HP1α do not exhibit major heterochromatin perturbations. Also, I am not convinced that the in vitro experiments, where HP1α and Ki-67 colocalize in condensates, recapitulate the situation in cells, where Ki-67 localizes to the periphery of chromocenters while HP1 localizes throughout the chromocenters. I have a hard time imagining how two proteins would phase-separate together if they do not really localize to the same place.

The reviewer keenly observes that in our high magnification images of Ki-67 immunofluorescence in neurons with loss of Anapc7, there does appear to be a rim of Ki-67 positivity surrounding chromocenters. However, we think the interpretation that this represents the general spatial relationship may be a bit misleading. For this reason, we now include a larger field, not just the zoomed in images (**Figure S4a-c**). These images show that this effect is only really observed in the Anapc7 mutant, in which Ki-67 is much more abundant. This effect is only rarely observed in control cells. This could also represent a side effect of formaldehyde fixation, which does not always preserve the true locations of disordered phase separated proteins (PMID 36444977). In addition, we now include new **Figure S8**, which shows the co-localization of endogenous HP1α and Ki-67 in control and ANAPC7 neurons (subject to the challenges discussed above, in response to comments by Reviewer 1).

Major points:

1. Mice lacking HP1α do not show any major phenotype (see for example PMIDs 21250545, 32020053) and mouse cells lacking HP1α still contain chromocenters with similar numbers and sizes (PMIDs 29166597, 37082862), suggesting that defects in HP1α function and phase separation can be compensated. This seems to be incompatible with the idea that phenotypes caused by loss/accumulation of Ki-67 arise from defects in HP1α function/phase separation. Rather, the effects on heterochromatin could be mediated by other proteins.

The reviewer is correct to point out that loss of HP1α does not necessarily change mouse chromocenters, although these effects can be cell type specific and it is possible that in some studies loss of HP1α is compensated by HP1β or HP1γ. As a field, we still have a lot of work to

do in order to understand the specific and redundant functions of the HP1 paralogs, especially in the context of phase separation and heterochromatin regulation in cells. Here, we focus on Ki-67-HP1α interactions as HP1α has a well-documented ability to undergo phase separation on its own but future studies should certainly address the properties of Ki-67 in the presence of HP1β and HP1γ. In a complex environment such as heterochromatin, it is indeed highly likely that other factors (proteins, RNA, chromatin itself) provide compensatory mechanisms for regulation. In this context, our study addresses the question of how other heterochromatin components such as HP1α binding partners (e.g. Ki-67) modulate these properties and how this may manifest as changes in chromatin organization.

2. The heterochromatin alterations that the authors describe in Fig. 1 seem moderate, with 3 chromocenters more in the absence of Ki-67, which corresponds to the standard deviations in the individual conditions. Could the authors clarify how they think this difference makes a functional difference for the cell? Do they have any suggestion where the heterogeneity in the wildtype situation originates from (cell cycle? differentiation stage?) and if these factors could be affected in the mutant cells?

Loss of Ki-67 has been investigated in many cellular systems over the years. However, Ki-67 has never been depleted from neurons, which are exquisitely sensitive to perturbations in chromatin regulation. In addition, dysregulation of Ki-67 in neurons is the only context linked to inherited disorders. However, in each of these systems, the effect of Ki-67 loss is typically modest at the cellular level. In that sense, our findings accord with previous studies.

There is indeed heterogeneity in these neurons, probably arising from the fact that they are primary neuronal cultures. Growing cells in a monolayer is necessary for high resolution imaging of nuclear features, which are far too crowded in the cerebellum *in vivo* to achieve cellular resolution. In mice, cerebellar granule neurons are produced in huge numbers from around postnatal day 4 to postnatal day 16. Cultures are typically prepared on day 6-7. This means that tissue dissociation inevitably intermixes cells at varying stages of differentiation. The culture process does promote differentiation and imposes a degree of synchrony, but this effect is far from universal. Cultured neurons also only remain healthy for about 5 days, imposing a relatively short window during which they can be studied. So the observed variability could definitely result from asynchrony.

3. The authors put forward the model that HP1α phase-separates in the wildtype condition. It is unclear how the authors' conclusions about Ki-67 then fit to this model. If Ki-67 enhances phase separation of HP1α, then loss of Ki-67 should dissolve chromocenters (make them larger and more numerous) while accumulation of Ki-67 should reinforce them (make them smaller/more compact, and fewer)? This is not what is observed in Fig. 1C/D. It would be helpful to explicitly spell out how the phase separation mechanism the authors propose would affect the parameters quantified in Fig. 1C/D.

The reviewer brings up an important point. Based on our results, we know the following: 1) Overabundance of Ki-67 in neurons leads to larger chromocenters, while depletion of Ki-67

leads to smaller and more numerous chromocenters; 2) Ki-67-enriched nuclear bodies (likely coinciding with chromocenters) exhibit properties consistent with phase separation (new data). 3) Ki-67's LRD domain is sufficient for chromocenter localization and the LRD domain interacts with HP1α (new data); 4) Ki-67*, which contains the LRD, can enhance the phase separation propensity of HP1α and chromatin. Based on these observations, we would expect that more Ki-67 would lead to enhanced phase separation of heterochromatin components and hence larger chromocenters (through fusion), and loss of Ki-67 would lead to less phase separation and therefore smaller chromocenters (due to less fusion). However, predictions about this relationship should be approached with a lot of caution and in the manuscript we avoid making this link as we still have a lot of work to do to understand the molecular interactions and mechanisms that drive this process. For example, one aspect that is very important but beyond the scope of this study is the effect on chromatin compaction. We know that HP1α can compact DNA and chromatin, but we do not yet know how the presence of Ki-67 affects compaction, which in turn would affect chromocenter size. In addition, as discussed above, it may be important to consider other players, including RNA, HP1β and HP1γ. We plan to pursue these detailed mechanistic questions in a future study.

4. The authors use 1,6-hexanediol to draw conclusions about phase separation, although it is quite clear now that 1,6-hexanediol is toxic, dissolves nuclear pores (PMID 12509452), induces chromatin condensation (PMID 33536240), affects kinases and phosphatases (PMID 33814344), and perturbs hydrophobic interactions rather than dissolving condensates, which could also be formed via electrostatic interactions that are not affected by 1,6-hexanediol (PMID 36526633). I acknowledge that the authors have put a word of caution when interpreting their experiments with 1,6-hexanediol, but I would strongly advice to not make any conclusions about phase separation based on these experiments.

We recognize the limitations of this reagent, as this reviewer documents, and appreciate the inclusion of these references. In the resubmitted version, we have attempted to describe this result judiciously and with the appropriate level of caution. However, we feel that this result remains relevant to the story, as the relocalization of Ki-67 occurs rapidly away from constitutive heterochromatin and into nucleoli upon hexanediol treatment. We have tried to avoid arguing that this observation conclusively demonstrates that the localization of Ki-67 within constitutive heterochromatin requires the process of phase separation. Other reviewers found these results to be interesting, but did request more in relation to our interpretation of this result, which we now provide in the resubmitted version. Nevertheless, as other reviewers raised issues with this experiment, we feel that it is reasonable to relegate this information to supplemental data.

In the resubmitted version, we also include new live-cell imaging data fusion and fission (i.e. liquid-like behavior) of RFP-tagged endogenous Ki-67 in neurons (**new Figure 2**). This provides more direct evidence for liquid-like behavior of Ki-67 enriched compartments.

5. The authors suggest a model in which Ki-67 modulates HP1α phase separation and thereby affects chromocenter number and size. In their 1,6-hexanediol experiments, where Ki-67 leaves from the chromocenters, they actually have a tool to directly test this. Surprisingly, they did not

quantify chromocenter sizes in these experiments. The prediction from the authors' model would be that the sizes change if I am not mistaken. Is this the case? If not, this speaks against a direct scaffolding role of Ki-67 in heterochromatin condensates.

As the reviewer points out above, hexanediol is toxic to cells, and this is certainly the case with neurons. The treatment distorts the nuclei of neurons, shrinking them and changing the arrangement of chromocenters in a manner that would preclude reliable quantitation of their size or number.

6. The in vitro work is interesting, showing that Ki-67 can phase-separate when different molecules are added, including DNA, RNA, nucleosome arrays, HP1α/pHP1α. However, I am wondering if these experiments really recapitulate the situation in cells, where Ki-67 localizes to the periphery of DNA/nucleosome-dense chromocenters (see first column in Fig. 2A, where Ki-67 forms rings around Hoechst-stained foci, most pronounced in Anapc7 -/- cells but also in control cells. Line profiles could help to better visualize this). In the in vitro experiments, no Ki-67 ring structures are observed upon addition of DNA, HP1α and nucleosome arrays, see for example Fig. 3B-E, Fig. 4D). From my perspective, it is crucial to explain this difference, as my naive interpretation would be that these components form condensates in vitro but not in cells (hence, lack of colocalization in foci in cells but instead Ki-67 rings around Hoechst foci). To clarify the relative localization of the individual components in the in vitro assays, it would help to also label DNA, RNA, nucleosomes arrays in addition to Ki-67, and acquire multi-color images.

As mentioned above, this effect is only really present in Anapc7 mutant neurons, where Ki-67 is overabundant. We favor that this more likely represents the consequence of supraphysiologic amounts of Ki-67 or, more possibly, even an artifact of formalin fixation. We have chosen high magnification images more judiciously in the resubmitted version (e.g. **Figure S4a-c, Figure S8**), while also including the larger fields (from which the zoomed in fields were drawn) in order to avoid giving the impression that Ki-67 surrounds constitutive heterochromatin rather than residing within it. For our in vitro work, **Figure S16** presents multi-color imaging of nucleosome arrays (12xNA), Ki-67* and HP1α.

7. I am surprised that previous work on Ki-67 and heterochromatin regulation is not discussed/cited, including PMID 26949251 and 37082862. Rather, the authors write in the Abstract that the role of Ki-67 in regulating heterochromatin during interphase is poorly understood. I am not sure if this accurately reflects the current state of the art.

We appreciate the reviewer's knowledge of the literature and regret these omissions. In the resubmitted version we discuss these studies in more detail (e.g. see new version of the discussion section).

Minor points:

- RNA-seq: It would be interesting to know if the levels of major satellite transcripts are altered.

We tested this directly and have included this information in the resubmitted version (**Figure S3e**). Loss of Ki-67 had no effect on the abundance of transcripts related to the major satellite repeat.

- I would not refer to a fragment of a protein as an "idealized" version of the protein (line 192).

We have changed the word “idealized” to “truncated”.

- For the in vitro condensates, it does not seem to be entirely clear which component plays the role of driver/scaffold and which component that of a client. Nucleosome arrays can form condensates on their own if the solubility in the buffer is low enough (see for example PMID 31543265), so condensates made with nucleosome arrays and any protein could arise from the latter binding to the arrays and reducing their solubility, as opposed the protein itself undergoing phase separation. The effect of histone PTMs could then be to reduce the solubility of the arrays or to modulate the affinity of the binder that makes it more/less soluble. Maybe the authors want to take this into account when discussing their results.

We agree that the properties of chromatin are very important here and the addition of nucleosome arrays increases the propensity for phase separation for both Ki-67* and HP1α, with and without phosphorylation. Our observations suggest that the addition of chromatin provides robustness to the system and tolerance to different histone PTMs. We discuss this in more detail in the revised manuscript (see new version of the Discussion section).

Reviewer #3 (Remarks to the Author):

This work presents new findings spanning animal, neuron, sequencing, and biochemical studies on the role of Ki-67 in heterochromatin. First, new mice models are created that remove a ubiquitin ligase resulting in accumulation of Ki67 (ANAPC7-/-, mimicking genetic disease with heterochromatin defects) and, for comparison, Ki67 deletion. These mice show interesting phenotypic and mutant viability differences and neurons from these mice are analyzed showing disrupted heterochromatin. Heterochromatin marks were not different in these cells, suggesting a different reason for the change in organization, as suggested by previous research regarding the effect of Ki67 on chromatin directly. Ki67 localization is altered by 1,6 hexanediol, going to nucleolus, implying some change in weak interactions that this solvent can alter. In vitro biochemical assays show that a minimal Ki67 protein phase separates with heterochromatin components – nucleosome arrays, DNA, RNA, and HP1alpha. Ki67* phosphorylation alters this phase separation while nucleosome modifications make small changes to the behavior. The work is exciting due in large part to the animal and cellular data as well as the fundamental link made to the biochemical experiments – this link could be strengthened further. The lack of genomic data accounting for the phenotypes is demonstrated but a better hypothesis could be put forward (and tested). Additionally, a big gap between the biochemical and cellular data leave some questions, and the biochemical data are extensive but the connection to the cellular features is not so clear and the final sections could be shortened to strengthen the work.

Experiments testing the connections made using mutagenesis in vitro and then in cells would help show the proposed features in vitro really explain the behavior in mouse/cells.

We appreciate the thoughtful comments and we have done our best to address them. In particular, to strengthen the link between *in vivo* and *in vitro* experiments, we have added new data and sections. For example, new **Figure 2** suggests that Ki-67 nuclear bodies in live neurons display properties consistent with phase separation, while new **Figure S8** demonstrates colocalization of endogenous Ki-67 and HP1α. Furthermore, new **Figure 3** demonstrates that the LRD domain is sufficient for localization into chromocenters. The LRD domain has a well documented link to HP1 proteins, which prompted us to to focus our in vitro work on on Ki-67*/HP1α interactions and phase separation. To further explore the interactions between the two proteins, we also present NMR data, binding assays and coarse-grained simulations, which collectively suggest the existence of complex electrostatic networks in Ki-67*/(p)HP1α condensates. While this precluded the use of mutagenesis to further test hypotheses in vitro and in cells, we plan to continue this work and characterize the interactions from the Ki-67* point of view in a future manuscript (NMR assignments for this construct will take some time but we are working on segmental labeling the protein to enable those). In addition, we have streamlined the section on post-translational modifications and moved some figures to the supplementary information to present a more unified message.

Major

Does it change the view of the field if the chromatin state is drastically changed but the markers are not? This should be expanded upon in the text.

In neurons of mice with loss of APC7 (and increased Ki-67) and mice with loss of Ki-67, the effects on chromatin state are rather modest. We observed no significant changes in the abundance or distribution of any of the histone modifications we examined. This indicates that Ki-67 is required for the spatial organization of heterochromatin in cells but that this process is not dependent on heterochromatin marks such as H3K9me3. Previous literature presents conflicting observations regarding the connection between heterochromatin Ki-67 and H3K9me3, where some studies observe that Ki-67 presence correlates with H3K9me3 (PMID: 26949251), while other studies have seen that reduction of Ki-67 levels can cause the redistribution of H3K9me3 without changing its levels (PMID: 36245428). The effects are likely cell type specific. We have expanded our discussion of these observations in the new version of the discussion section.

It seems like a big jump to go from “not detect substantial changes in transcriptome” to “phase separation of nuclear components”. I think the connection should be made much more clear. I don’t think the biochemical experiments are really explaining the lack of difference.

In the revised version of the manuscript, we have tried to establish a stronger link between the observations *in vivo* and *in vitro*. For example, we now show that Ki-67 enriched compartments in neurons exhibit properties consistent with phase separation (i.e. fusion and fission in new

Figure 2). We also show data that strengthens the previously reported molecular connection between HP1α and Ki-67 using NMR data, binding assays and simulations. While it is still challenging to explain the lack of significant effects on PTMs or transcription, our results are not inconsistent with previous literature (e.g. PMID: 36245428) and are important to report as they describe a context that has not been explored before, namely the effects of aberrant Ki-67 in the developing brain. There is undoubtedly much more work to do to understand the mechanism behind these observations and the reviewers have given us many ideas regarding hypotheses to explore but they are beyond the scope of this manuscript. In the revised version of the manuscript, we strive to be more clear about our observations and to avoid overinterpretation.

Also, could the changes in the cells be checked earlier for example immediately after fertilization or with in vitro fertilization on zygotes rather than in neurons?

This question is beyond the scope of the manuscript, both technically and conceptually.

I understand that expressing a 3000+ residue protein is probably not so feasible in bacteria, but the authors could try in bacteria and in insect or mammalian cells to do so. It is not clear why the FHA domain is not included and surprising that it doesn't make a difference. The justification for the exclusion of domains should be better. Perhaps adding each one and showing they don't do anything in the assays?

To perform the biochemical and biophysical experiments in this manuscript, including the newly included NMR data and binding assays, we needed a construct and a recombinant cell production system with robust expression and yields. Therefore, we chose *E. coli* and constructs of 500 amino acids or less. To decide on the final construct, we performed localization studies of various Ki-67 constructs in N2a cells, where the LR domain demonstrated robust localization to chromocenters (while the FHA domain did not, see new **Figure 3**). In addition, based on the study described in Yamazaki et al., Nat. Cell. Biol. 2022 (PMID: 35513709), we added two copies of the R12 repeat to represent the properties of the extensive repeat region of Ki-67, including phosphorylation sites. To make our reasoning more clear, we have included a new main figure (**Figure 3**) and a more extensive explanation in the main text.

Quantification of the images of biochemical assays could help improve insight from this work.

For our in vitro condensate assays, we use turbidity assays as a quantitative measure of condensate formation and fluorescence-based images as a qualitative based measure of condensate formation and protein localization.These two independent approaches agree well in terms of the trends that we observe. Due to the large number of conditions we imaged, we did not image with quantification in mind and, instead, we focused on reporting representative images.

How is 1,6 hexanediol working in cells? The figure showing the difference should be more clear and have quantification of the difference in panel A and not just the nucleolar localization if

possible. Does it do anything in the biochemical assays? Recent work on hexanediol says it doesn't work on charged condensates (https://pubmed.ncbi.nlm.nih.gov/40281357/ from Fawzi and coworkers), so what is it doing here? It sounds interesting and useful to show this, but the mechanism may not be due to Ki67 directly but rather to other cellular components and this should be commented on and explored in vitro.

Panel **f** of this figure (now **Figure S4**) does quantitate the overlap between Ki-67 and chromocenters, a complementary method of demonstrating the relocalization of Ki-67 away from heterochromatin upon hexanediol treatment. We agree with the reviewer that the hexanediol experiments are not easy to interpret and based on the other reviewers' feedback, we have relegated them to the supplementary information, with cautious discussion in the main text. Instead, we emphasize new **Figure 2**, which shows that Ki-67 rich nuclear bodies can undergo fusion and fission in live neurons.

The real strength of the biochemical assays is showing the condensation and the co-condensation. The meager effects of most of the modifications could be addressed much more briefly and placed in SI in my opinion. This would streamline the manuscript considerably. The lack of DNA binding domain and other domains from Ki67* (see comment above) is concerning and could be addressed. Cellular studies with domain deletions could also be performed – the assay on Ki67* in cells is good but co-localization with Ki67 endogenous should be demonstrated.

In the resubmitted version, we include mCherry-tagged constructs expressing the FHA, N-terminus and charged patch domains of Ki-67 (new **Figure 3**). None of these are localized to constitutive heterochromatin. Expression of the FHA DNA-binding domain results in fluorescence throughout the cell including the cytoplasm, indicating that this region of Ki-67 is insufficient to bring Ki-67 to heterochromatin. The N-terminus and charged patch, in contrast, localize to nucleoli. These observations lead us to surmise that the Ki-67* fragment is the most reasonable combination of domains to examine the function of Ki-67 in minimalist systems. To address the reviewer's comment regarding the PTMs (new **Figure 8**), we have streamlined this section to give a more unified message and moved some data to the supplementary information.

The bleach regions appear different in that some drops show apparent bleaching of the region surrounding. Therefore, is the normalization done to a region in the same drop that is outside the bleach region? Or is it normalized to a distinct droplet? If the latter, then there is no way the bleach region will recover above the unbleached region in same droplet. So larger droplets (higher concentrations) could be performed to remove such complications.

We thank the reviewer for pointing this out. In the new **Figure 6**, we have provided new images for some FRAP experiments, e.g. pHP1α/Ki-67 combination in panel **a**, where we have much less bleaching of the surrounding area. Regarding normalization, we divided the bleached data by the pre-bleach intensity of the same droplet, and then multiplied by a reference droplet

adjustment factor, which takes into consideration any changes in fluorescence intensity of another droplet that was not bleached (e.g. to correct for photobleaching due to imaging or any changes in the sample as a whole).

As described above, mutants that disrupt functions/localizations in cells could be compared in vitro to see if they disrupt phase separation. This would strengthen the claim that Ki67 is biologically relevant partner through phase separation. For example, it is not surprising that changing the charge by phosphorylation alters the phase separation of, or interactions with, something that is highly positively charged. Could variants altering the charge or modification sites in cells be performed to show that the changes in test tubes are found in cells?

As Reviewer 1 also had a similar concern/suggestion, we spent a significant amount of time on this issue. As demonstrated by our NMR and binding data, we have shown that Ki-67* contacts the canonical HAC/PXVXL binding site on HP1α, yet our attempts to mutate the HAC/PXVXL-binding sites on Ki-67* did not reduce binding. Our simulations data, on the other hand, suggests that the charge patterning on the Ki-67* construct results in a complex electrostatic interaction network between Ki-67* and HP1α. As these interactions are quite extensive, we reasoned that extensive mutations may be required to disrupt the binding network and did not pursue those in cells. Instead, we will be following up on these observations in a future manuscript, as we have similar results with other HP1α binding partners. To address this important point in the current manuscript, we have added a new results section and figures (**Figure 7, Figure S12**) where we present and discuss the binding assays, the NMR interaction data, and the simulations.

The statement about “in vivo data support the conclusion … phase separation” could be improved by commenting in parallel on the in vitro work in the same sentence / paragraph. That is the strength here.

We have removed this statement and revised the discussion section where we interweave the discussion of *in vivo* and *in vitro* data more comprehensively.

Minor

Abstract:

“defective proteomsomal degradation” – of what?

We have added the clarification “proteasomal degradation of mitotic proteins”

The hypothesis that “Ki67 cooperates with HP1alpha …” articulated in the introduction should be explicitly mentioned here

To summarize our hypothesis in the abstract we have included the following statement: “These observations suggest that Ki-67 may regulate heterochromatin through a phase separation mechanism involving additional heterochromatin components.”

“that appears robust to” vague and weak, rephrase

The statement now reads: “Furthermore, we demonstrate that an engineered Ki-67 construct enhances the propensity of HP1α and nucleosome arrays to form condensates *in vitro*, a process that is mediated through a complex network of electrostatic interactions and is robust to relevant histone post-translational modifications.”

“suggest that Ki67 may influence” weak – perhaps just “may influence” or “suggest”

The statement now reads: “These observations suggest that Ki-67 may regulate heterochromatin through a phase separation mechanism involving additional heterochromatin components.” We prefer to keep the more cautious language as there is still a lot of work to be done to elucidate the mechanism of this process.

Introduction:

“at smaller scales” confusing

We have clarified this as: “at smaller length scales”.

“and/or epigenetic state” not clear

We have changed this to “dysregulation of chromatin state”.

“multiplicity of neuronal functions” give some examples and maybe “myriad” or “many” is correct word

This expression is no longer used in the introduction.

“unsurprising” used in two sentences in a row!

We have fixed this issue.

“Ki67 accumulates within” mention how this was demonstrated?

Since this is a statement in the introduction, we want to keep the focus on the result rather than the method. We have included the relevant reference at the end of the sentence, which shows that this conclusion was obtained through imaging experiments.

“Compared to its role in mitosis” rephrase to make more clear

Statement now reads: “Compared to its role in mitosis, which is well documented, how Ki-67 contributes to the formation and function of heterochromatin especially in the disease-relevant context of differentiating neurons remains poorly understood.”

“which is implicated to play a role” à “which plays a role” “which is thought to play a role”

We have changed this statement to “which is thought to play a role”

Results

Explicitly state that the human condition is recessive(?)

The introduction now clearly states: “For instance, the *ANAPC7* neurodevelopmental syndrome (Ferguson-Bonni syndrome, OMIM 619699) is an inherited recessive disorder of cognition mechanistically linked to dysfunction of the heterochromatin compartment.”

That the Sox2 promoter Cre results in global knockout should be demonstrated or at least a reference for this method as seems that it is not the standard way to generate a null.

We respectfully disagree that this is a non-standard method of generating null alleles from floxed alleles (e.g. see PMID: 14516668). Sox2-cre is an extremely useful line for generating these because it is expressed in oocytes and the cre protein persists after fertilization, meaning that the floxed region (of Mki67 in this case) gets excised at the one cell stage when the floxed allele is brought in via the sire. However, the next generation will be heterozygous for a null allele (Mki67 in this case) with the other copy being wild-type, but without the need to harbor the cre in this first generation. This strategy accelerates the production of useful animals and simplifies the subsequent breeding. Of course we verified that Ki67 is null by RNAseq and immunofluorescence.

“a membrane free organelle that forms” delete this and say “HC may be a distinct nuclear compartment formed through phase separation”

Done.

The domain structure in Fig3A should be shown in figure 2 perhaps and the features that are common to phase separation should be shown aligned with the domain structure

Since we have new figures, the relevant figure is new **Figure 3**, where we show the localization of different domains. We have included the domain structure cartoon as panel **a** in this figure.

“its charge of +33 at pH 7.2” this is predicted charge and doesn’t account for pKa changes probably. add “predicted”

Done.

Ki-67* is also called R12,2-LRD which is confusing to use both, or am I not correct (are these the same?)

Yes, they are the same. In new **Figure 3**, we clarify that R12,2-LRD is the same as Ki-67* (see panel **b**). We felt that it is important to show that here, as we separately test the LRD alone and the R12 repeat alone. Everywhere else in the manuscript, we use Ki-67*.

The text could mention if the pHP1alpha is pS/pT/pY etc and how many sites approx

We have clarified this in the methods section: “Mass spectrometry analysis has indicated that this protocol results in the predominant phosphorylation of S11, S12, S13, and S14 on the HP1α N-terminus, with relatively minimal phosphorylation elsewhere in the sequence.”

Response to the reviewer comments

We appreciate the reviewers' feedback in response to our resubmitted manuscript as these experts all raised important technical and conceptual criticisms in the previous round of review that we worked hard to address. We are relieved to hear that Reviewers 1 and 3 had no significant reservations about our resubmitted version and were supportive of its publication. Reviewer 2, however, continues to harbor some reservations about our central claims, which we address below.

Reviewer #1:
In this revised manuscript, Adasi (sic) et al have spend quite some efforts in addressing the referee's comments. I appreciate they took into account several suggestions and clarified points that could have been confusing in the previous version. I appreciate that some requests (although still standing as important) have proven technically challenging and cannot be addresses in full. Overall, I am happy with the changes and I am in favour of accepting the manuscript in this present version.

Only one point that should be modified is that the reference "Stamatiou, K. et al. Multi-omic analyses reveal a differential contribution of chromatin-associated PP1 holoenzymes to mitotic exit and G1 re-establishment. bioRxiv, 2024.2004.2029.591626 (2024). https://doi.org/10.1101/2024.04.29.591626. Now it is published and should be quoted as DOI: 10.1016/j.devcel.2026.02.016

We thank the reviewer for their invaluable contributions throughout this process, and for bringing this specific issue to our attention. We have substituted the appropriate reference in the newest version.

Reviewer #2:
The authors have revised their manuscript. Unfortunately, they did not address most of my points. While this paper presents some interesting data on Ki-67, for example the effect of Ki-67 depletion on the viability and development of mice, and the lack of any effect on the transcriptome and the epigenetic state, I am not convinced that the central model of the paper is sufficiently supported, namely the model that Ki-67 organizes heterochromatin via phase-separation.

We appreciate the reviewers' concerns and after reexamining this reviewer's initial feedback, and our response to it, we acknowledge that we could have addressed those concerns more adequately. We understand the nature of the issues raised, and we hope that the manuscript changes we have made will improve upon these sticking points.

We have framed our model in the context of heterochromatin phase separation as we observed fusion and fission of Ki-67 nuclear bodies in neurons, Ki-67 has been reported to form condensates as part of its role in mitosis, and it is a documented binding partner of HP1α, which can also form condensates in the presence of chromatin and DNA. In addition, the two proteins show robust phase separation *in vitro* which is not always the case for HP1α and its binding partners (we have investigated a number of binding partners and hope to post those results on BioRxiv shortly). We acknowledge that supporting the *in vitro* work with inequivocal proof that the proteins function through a phase separation mechanism in cells is technically very difficult but altogether we believe that our observations are consistent with a model where modulation of phase separation of heterochromatin components such as HP1α/pHP1α can impact the size and number of neuronal chromocenters.

Nevertheless, the reviewer's comments prompted us to take a closer look at our data and compare our observations with published literature that supports alternative models for heteronuclear proteins. For example, Pantier *et al., Nat. Comm.* 2024 (PMID: 38719804) argued against a phase separation mechanism for the heterochromatic protein MeCP2 based on analysis of the expression levels of the protein in mouse firboblasts. Part of their argument was based on the following observations: 1) as MeCP2 expression level increased, the concentration of the protein increased both inside the chormocenters and the nucleoplasm, while the ratio of chromocenter concentration/nucleoplasm concentration remained relatively constant; 2) the number and size of chromocenters did not change substantially. These observations suggest that the chromocenters don't have the ability to buffer the concentration of MeCP2 and that increased concentration of MeCP2 inside the chromocenters is just a function of the increased concentration of the protein inside the nucleus. In our case, we can compare these parameters for our control group of neurons (expressing endogeneous levels of Ki-67) and our ANAPC7-/- neurons (expressing pathological amounts of Ki-67). This comparison reveals that: 1) In the ANAPC7-/- neurons, where there is more Ki-67, the ratio of chromocenter Ki-67 abundance to nucleoplasm abundance is significantly higher compared to the controls (see figure below); 2) The size of the chromocenters increases (as reported in main text Fig. 1c). This suggests that as the concentration of Ki-67 in the nucleus increases, the chromocenters expand to accommodate the increased amount of protein, a property which is consistent with a phase separation model.

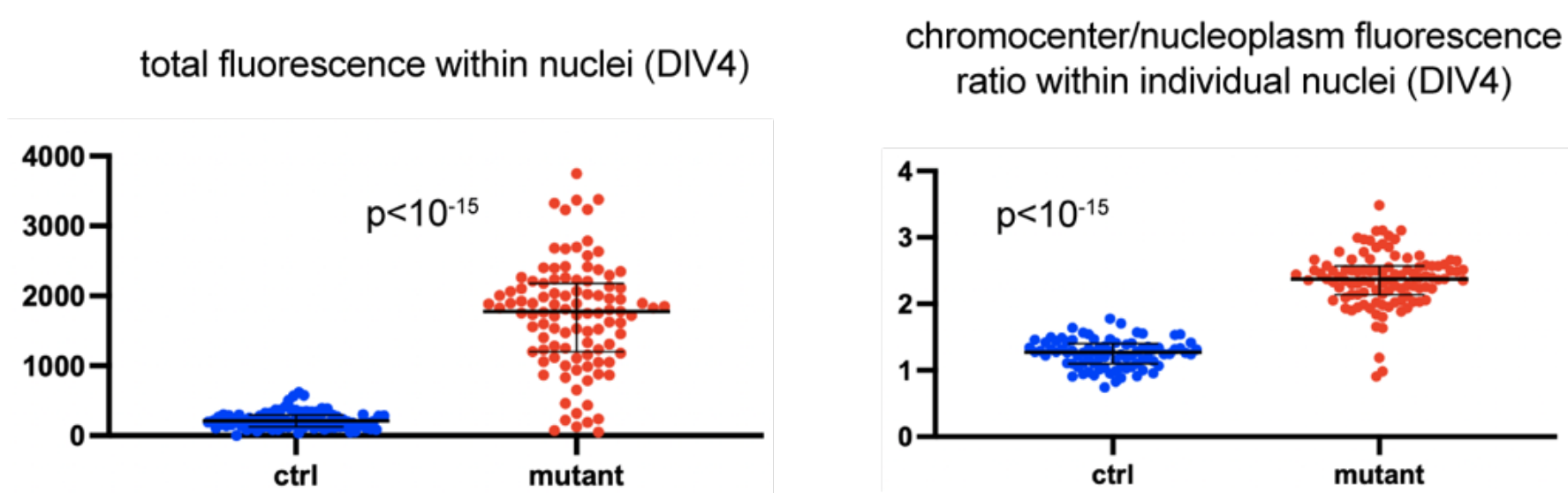

Response figure. Analysis of the distribution of Ki-67 fluorescence between the chromocenters and the nucleoplasm of DIV4 neurons from control mice (endogeneous amounts of Ki-67) and the $ANAPC7^{-/-}$ mice (overabundance of Ki-67).

However, we acknowledge that the heterogeneity in our neuronal cultures possibly masks some of these effects (see discussion below) and that additional/alternative mechanisms may also be at play. Therefore, we have expanded the discussion in our manuscript where we have added the following paragraph where we more clearly state why we suggest a phase separation mechanism and where we still have more work to do.

"Taken together, our genetic mouse models, cellular experiments and *in vitro* biophysical work suggest that Ki-67 plays a role in heterochromatin organization in developing neurons, and that this function may be mediated through a phase separation process that involves interactions between the Ki-67 LRD and HP1α. This phase separation process is likely distinct from the liquid coat formed by phosphorylated Ki-67 at the end of mitosis, which instead relies on its N-terminal charged patch[39]. While the role of phase separation in heterochromatin formation and organization has been actively debated in the literature[29,75-79], our model is supported by the observed changes in chromocenter number and size as a function of Ki-67 depletion or

overabundance, the high degree of co-localization between Ki-67 and H3K9me3 and HP1α in cells, the ability of Ki-67 nuclear bodies to undergo fusion and fission in live cell imaging of neurons, and the robust phase separation observed between Ki-67* and various heterochromatin components *in vitro*. The increase in chromocenter size as a function of Ki-67 concentration (comparing control vs Ki-67 enriched *Anapc7*$^{-/-}$ neurons), in particular, supports a "concentration buffering" mechanism consistent with phase separation, rather than the "size buffering mechanism" proposed for the heterochromatin protein MeCP2 where the chromocenter size and number did not change as the expression levels of MeCP2 were modulated.[77] Nevertheless, we acknowledge that alternative models may also be able to explain our observations and that more work is necessary to unequivocally support the Ki-67 heterochromatin phase separation mechanism in mouse cells (e.g. FRAP experiments which we were unable to perform due to the low sensitivity of RFP-tagged Ki-67 in live neurons) and human neurons, which differ substantially in heterochromatin organization and localization.[77] We also cannot rule out the involvement of other heterochromatin components, including proteins and RNA that have been shown to modulate phase separation[51,80,81]. Finally, while our *in vitro* work focused on a minimal construct of Ki-67 rather than the full protein, it is possible that other Ki-67 domains also contribute to its heterochromatin functions, even if they are not essential for localization to these compartments. It will also be important to further investigate the exact nature of Ki-67 and HP1α interactions, which exploit the binding interface of HP1α but do not appear to rely on a classical HAC/PXVXL-binding motif in the LRD."

- I do not see any impressive colocalization between Ki-67 and chromocenters, which would be required for such a phase separation model (see for example Fig. S4a - Hoechst and Ki-67 do not overlap). There is a colocalization in Fig. S8, when cells are fixed with methanol:acetone, but it is clear that the nuclear organization is heavily perturbed under these conditions, as the authors state in the rebuttal letter, so that this colocalization is not representative of a living cell.

- I do believe that some Ki-67 binds to heterochromatin, which has also been documented in the previous literature (see references mentioned by me and the other reviewers in the first round of review). However, for condensate formation via phase separation, partial colocalization is not enough, because the concentration jumps where the dense phase forms, leading to a very prominent colocalization, not a partial colocalization, and a pronounced phase boundary between the coexisting phases. Partial colocalization and mixed localization at heterochromatin and other sites (nucleoli) is much more consistent with a model in which Ki-67 does not phase-separate but bind with some moderate affinity to heterochromatin sequences (and to sequences in nucleoli).

We address these related comments together below.

We respectfully disagree with the idea that Ki-67 only partially overlaps with chromocenters, but acknowledge that we needed to modify some of the figures to clarify this observation, which we continue to believe is compelling and well-supported by both our data and previously published data.

In the resubmitted version of Figure S4a referenced above, we did not show the overlap between the Ki-67 and Hoechst signals, just the overlap between Ki-67 and nucleolin. Ki-67 and nucleolin indeed only overlap to a limited extent. In the latest version we have included merged images of Ki-67 with Hoechst (see new Figures S4 and S5). However, the observation that Ki-67 overlaps with Hoechst in neurons is not new, as this represented a key observation in the Ferguson *et al., Mol. Cell* 2022 (PMID: 34942119) paper. Some excerpts from Figure 7 from this paper are shown below. As far as immunofluorescence images of neurons go, these show a large degree of overlap between signals, with both displaying a very specific distribution in the nucleus.

[editorial note: third party material redacted]

Above: Image of the cultured cerebellar granule neurons from Cdh1 mutant mice, which are also impaired in the degradation of Ki-67. Ki-67 and H3K27me3 exhibit non-overlaping distribution in nuclei, whereas Ki-67 overlaps extensively with Hoechst. Figure published in Ferguson *et al., Mol. Cell* 2022 (PMID: 34942119).

Additionally, in our current manuscript, we quantitate the colocalization between Ki-67 and Hoechst in Figure S4f (below) in cultured cerebellar granule neurons. The Pearson R values

averaged 0.6 for control and APC7 mutant neurons under baseline untreated conditions (no hexanediol), which reflects a high degree of correlation between these signals.

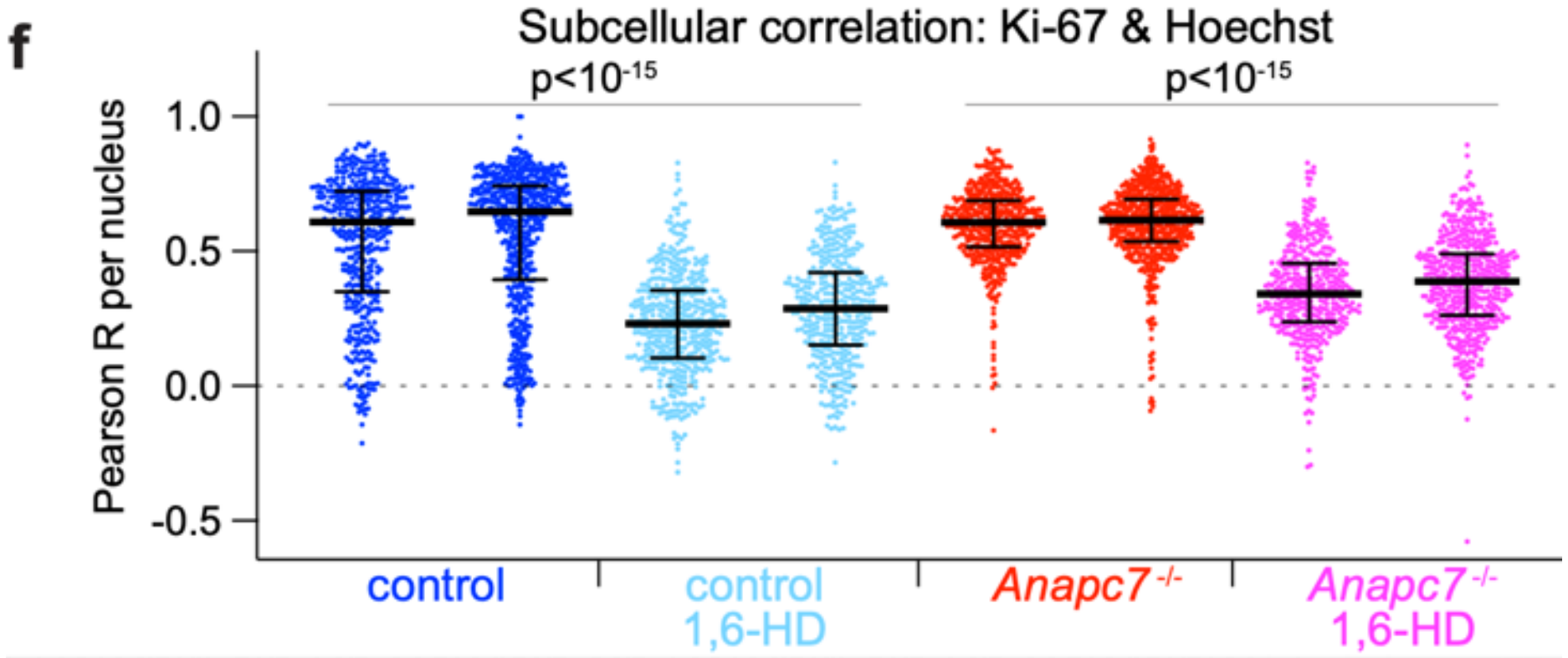

Above: Reproduction of Fig. S4f from this manuscript where we quantitate the colocalization between Ki-67 and Hoechst.

Finally, in other figures we detect Ki-67 without immunofluorescence but instead using RFP-tagged endogenous Ki-67. In our new version of Figure S5a below, there is nearly 1:1 overlap with Hoechst in the newly included merged images of Ki-67-RFP and Hoechst on the right. There is no rim of Ki-67 in these images, just as there is no rim of Ki-67 in the heterochromatic structures in the live cell imaging in Figure 2. This rimming effect appears to be seen only when Ki-67 is detected using immunofluorescence. This leads us to believe that it is actually an artifact of antibody staining, potentially as a result of reduced access to the interior of large and highly compacted constitutive heterochromatin structures by the primary and/or secondary antibodies or the complex between these. While we wish to have been able to record FRAP data in our Ki-67-RFP cells, the fluorescence intensity of our Ki67-RFP signal was not sufficient for FRAP imaging experiments. Nevertheless, we observe dynamic behaviors such as fission and fusion in live-cell imaging (Figure 2), which are consistent with the formation of dynamic condensates.

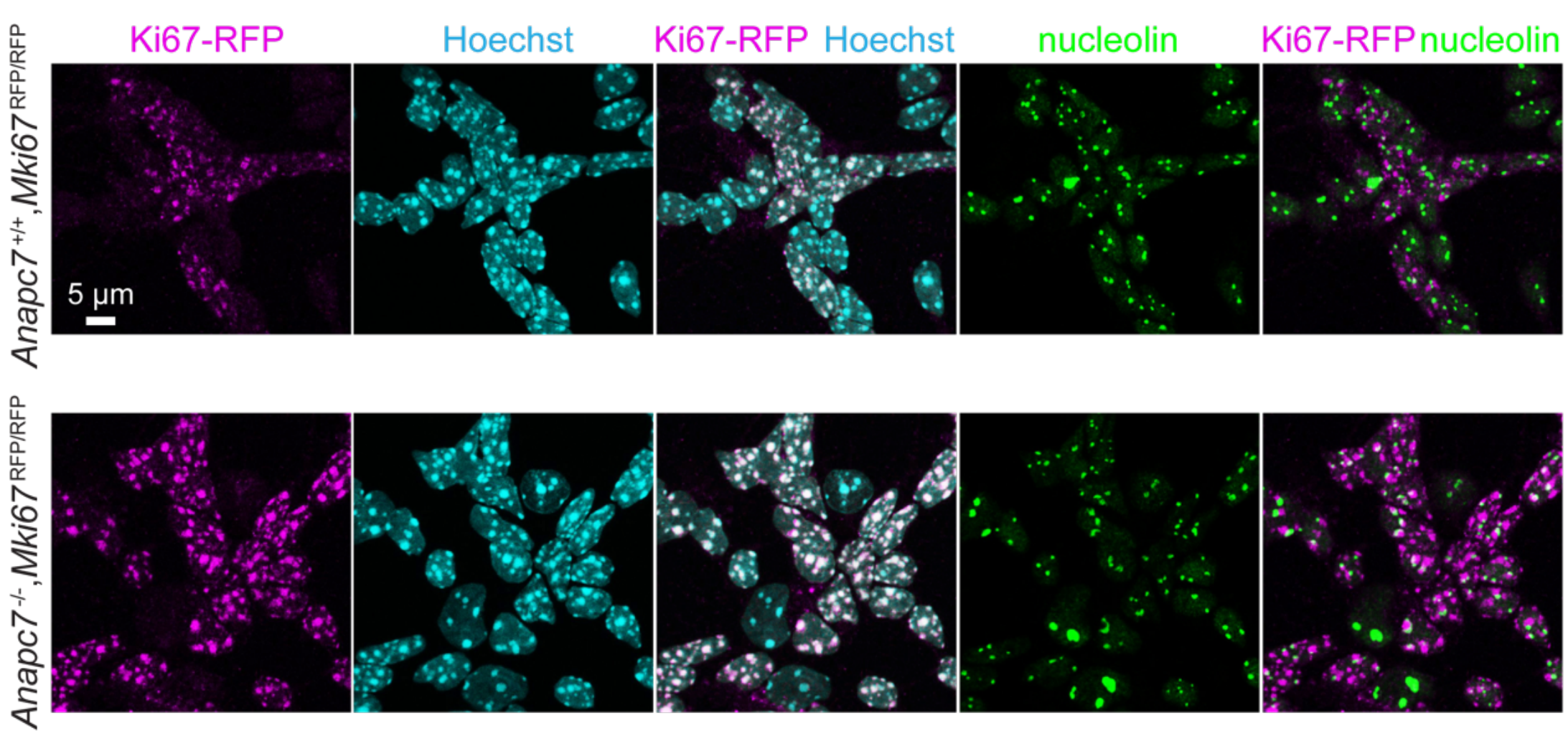

Above: Reproduction of newly revised Figure S5a, which now includes an overlay between Ki67-RFP and Hoechst.

- I don't see statements like "Perturbing Ki-67 in differentiating neurons disrupts heterochromatin organization" supported, just because there is a moderate change in chromocenter numbers, within the standard deviation of chromocenter numbers observed in unperturbed cells. Without Ki-67, chromocenters look very similar to those in wildtype cells (Fig. 1a), the epigenetic state and the transcription at heterochromatin does not change. To me, it remains unclear if Ki-67 has a major impact on chromocenter organization and heterochromatin function. I am also missing any serious attempt to explain the mouse phenotypes in terms of viability and development by the moderate changes seen at heterochromatin.

The difference between the genotypes is indeed subtle. But the nature of primary neuronal cultures is that they are heterogeneous in terms of their developmental stage, which probably accounts for the variability we observe. Nevertheless, we find reproducible changes in heterochromatin organization in terms of both the number and size of chromocenters. Reassuringly, the direction of changes is consistent (i.e. changing in opposing ways) when Ki-67 is lost (Ki-67 mutant) versus overabundant (APC7 mutant). The subtle effects are in some ways expected – if a mutation severely disrupted heterochromatin formation, it would likely be incompatible with neuronal survival due to the sensitivity of neurons to perturbations in chromatin organization. This consideration is directly relevant to the observed variability in organismal viability. A key limitation of our study is that the time points at which we assay chromocenter organization using genomics and imaging occur after the critical developmental window that determines survival in Ki-67-deficient mice. As a result, our analyses are necessarily biased toward neurons derived from mice that have passed this window. This selection effect may obscure stronger heterochromatic phenotypes that may occur earlier in development in embryos that fail to survive. Consistent with this idea, several studies (Singleton *et al.*, *Neurobiol. Dis.* 2012 PMID: 22433082, Meshorer *et al.*, *Dev. Cell* 2006 PMID: 16399082, Alvarado *et al.*, *Biol. Open* 2025, Aoto *et al.*, *Dev. Biol.* 2006 PMID: 16950240) have shown that chromocenter organization undergoes dynamic redistribution during neuronal differentiation and development, suggesting that proper spatial organization of heterochromatin is developmentally regulated and potentially critical at specific stages. It is therefore plausible that Ki-67 plays a more consequential role during this early window, when chromocenter reorganization is actively occurring, and that defects at this stage could underlie the reduced viability observed in a subset of animals. By the time neurons are analyzed in our system, compensatory or redundant mechanisms may have partially restored heterochromatin organization, resulting in the relatively mild phenotypes we detect.

We have avoided speculating about the reduction in viability of mice with global Ki-67 loss. The wide phenotypic spectrum makes this a somewhat perplexing phenotype. There appears to be a window during embryonic development when mice are susceptible to Ki-67 deficiency. If they make it through that period, some even live a normal lifespan without obvious significant phenotypes. However, most that are born are obviously affected and die prematurely. The mice are on a congenic background, so this is unlikely to result from modifier alleles from different strain backgrounds. Variable phenotypes like this often result from defects in processes that are both stochastic and under redundant control (e.g. impairment of the Hsp90 chaperone in *Drosophila* as discussed by Rutherford & Lindquist *Nature* 1998 PMID: 9845070 and Queitsch *et al.*, *Nature* 2002 PMID: 12050657, or the impairment of *C. elegans* intestinal differential as discussed in Raj *et al., Nature* 2010 PMID: 20164922). Maintenance of the integrity of constitutive heterochromatin fits this bill, as constitutive heterchromatin is required for genome stability, but Ki-67 is just one of many proteins that influences this process. Therefore, when Ki-67 is lost, supplemental mechanisms may occasionally allow heterochromatin to function more or less normally. Ki-67 is also required to prevent chromosome entanglement, so when lost it is possible that there is mis-segregation of genetic material. If this occurs at an early stage of development, then non-viability

would result. We have done some preliminary experiments (e.g. analyze markers of DNA damage, etc) to look for these consequences of Ki-67 loss, but these have not been illuminating so far. Given the uncertainty, and the fact that this phenotype lies outside of the central focus of our study, we prefer to avoid speculating at this stage and instead pursue this interesting direction in follow up studies.

- The authors insist on interpreting experiments with hexanediol and on drawing conclusions about cells based on in vitro experiments. I am not convinced that this can be done here. The in vitro system is highly simplified and behaves differently from cells (HP1/Ki-67/chromatin perfectly co-localize, while in cells they don't - this is not a detail, because co-localization is at the core of a phase separation mechanism). Hexanediol is known to have plenty of side effects and to be toxic. I have no doubt that a large disordered protein like Ki-67 phase-separates in vitro, and I am also not surprised that it binds chromatin arrays, as it is a chromatin-binding protein. However, I don't see that these data can be used to justify the model that Ki-67 organizes heterochromatin in cells through phase-separation, especially due to the mild phenotypes seen upon depletion in cells (most chromocenters are still there) and the poor colocalization between Ki-67 and chromocenters in cells.

We have attempted to interpret hexanediol experiments with caution and tried to do a better job laying out the caveats. Other reviewers found these experiments to be informative, so we are reluctant to eliminate them from the manuscript. Instead, we tried to bring focus to Figure 2 of the manuscript where we show that Ki-67 nuclear bodies can undergo fusion and fission, which is the key result in live cells that points to a phase separation mechanism. Therefore, while we still kept the hexanediol experiments, we moved them to the supporting information and do not view them as key supporting evidence for the phase separation mechanism we propose.

Regarding the second point that Ki-67 (or rather our minimal Ki-67* construct) undergoes phase separation *in vitro* is not surprising, we respectfully disagree. Our experience studying several HP1α binding partners suggests that not all binding partners have these capabilities (manuscript in preparation). In addition, this work provides several important observations: 1) Ki-67* significantly lowers the threshold for pHP1α phase separation, so that both proteins can do so at endogeneous concentrations and physiological salt conditions. This is important as pHP1α/HP1α on their own only undergo phase separation under non-physiological conditions *in vitro*. Therefore, this argues for a model where this potential function of HP1α is regulated by its binding partners, a topic that we explore further in our other manuscript in preparation. 2) The experiments with nucleosome arrays on the other hand show that this process is enhanced in the presence of chromatin and is largely independent of relevant PTMs, setting the stage for more detailed investigations of protein-protein and protein-chromatin interactions in these environments. While we agree that the *in vitro* experiments are not sufficient on their own to support a phase separation mechanism in cells, when placed in the context of our other cellular observations (fusion and fission of Ki-67 domains, co-localization with chromocenters, and changes in chromocenter size and number as a function of Ki-67 abundance as discussed above), they provide valuable mechanistic insights into the interactions that may drive this process.

Reviewer #3:
The authors have addressed my comments thoroughly in their replies and updated manuscript.

We thank the reviewer for their suggestions, which significantly helped us improve the manuscript.

Reviewer #2 (Remarks to the Author):

The authors have revised their manuscript and now acknowledge more clearly the limitations and open questions. I think this improves the paper. I have two remaining points below that the authors may want to consider, but I do not want to delay the process given that the other reviewers appear satisfied.

1. For the colocalization analysis in Fig. S4f, it would be helpful to also look at the colocalization between HP1alpha and Ki-67 and between HP1alpha and Hoechst. With such an analysis, one could put the other numbers (Pearson R values for Ki-67/nucleolin etc) into context. At the end, a large part of the paper and the resulting model revolve around Ki-67 phase-separating with HP1alpha, and I would find it therefore important to quantify this in cells. In the best case, this would be via immunostaining against HP1alpha, otherwise via co-expression of HP1alpha tagged with GFP or some other fluorescent protein.

Since this was also brought up by one of the other reviewers earlier in the review process, we invested a great deal of time and effort into trying to obtain HP1alpha images of the granule neuronal cultures. We tested several commercial antibodies to HP1α with limited success, attempting many different fixation and detergent conditions. The CST reagent worked to a degree in immunofluorescence, but detection required methanol/acetone fixation, which distorts the nuclear architecture of primary neurons. While we provide some images of HP1α immunofluorescence in these cultures in Supplementary Fig. 8 to demonstrate co-localization with Ki-67, the imaging quality is not sufficient for proper quantification. Since these are primary neuronal cultures, co-expression of HP1alpha tagged with GFP would be challenging.

2. The observation that Ki-67-positive particles undergo fission is cited in support of a phase separation model (Discussion, page 17). However, classical phase separation theory does not predict fission: condensates are expected to grow over time, be it through Brownian coalescence or Ostwald ripening, toward the energetically favorable state of reduced interfacial area. Fission can occur in phase-separating systems, but it requires invoking mechanisms beyond the classical phase separation theory itself, such as active processes that mechanically fragment condensates, potentially an actively imposed change in condensate composition, etc. Since phase separation is an inherently passive process, fission would need to be explained by extensions or modifications of the phase separation model, not by that model alone. But obviously, other models could also be extended to explain fission, so I would not cite this observation in support of phase separation.

We thank the reviewer for pointing this out and we no longer cite the process of fission in support of our phase separation model.
